# Mechanism of antibody-specific deglycosylation and immune evasion by *Streptococcal* IgG-specific endoglycosidases

**DOI:** 10.1038/s41467-023-37215-3

**Published:** 2023-03-27

**Authors:** Beatriz Trastoy, Jonathan J. Du, Javier O. Cifuente, Lorena Rudolph, Mikel García-Alija, Erik H. Klontz, Daniel Deredge, Nazneen Sultana, Chau G. Huynh, Maria W. Flowers, Chao Li, Diego E. Sastre, Lai-Xi Wang, Francisco Corzana, Alvaro Mallagaray, Eric J. Sundberg, Marcelo E. Guerin

**Affiliations:** 1 Structural Glycobiology Laboratory, Biocruces Health Research Institute, Barakaldo, Bizkaia 48903 Spain; 2 Structural Glycobiology Laboratory, Center for Cooperative Research in Biosciences (CIC bioGUNE), Basque Research and Technology Alliance (BRTA), Bizkaia Technology Park, Building 801A, 48160 Derio, Spain; 3 Ikerbasque, Basque Foundation for Science, 48009 Bilbao, Spain; 4 Department of Biochemistry, Emory University School of Medicine, Atlanta, GA 30322 USA; 5 University of Lübeck, Center of Structural and Cell Biology in Medicine (CSCM), Institute of Chemistry and Metabolomics, Ratzeburger Allee 160, 23562 Lübeck, Germany; 6 Department of Microbiology and Immunology, University of Maryland School of Medicine, Baltimore, MD 21201 USA; 7 Institute of Human Virology, University of Maryland School of Medicine, Baltimore, MD 21201 USA; 8 Department of Pharmaceutical Sciences, University of Maryland School of Pharmacy, Baltimore, MD 21201 USA; 9 Department of Chemistry and Biochemistry, University of Maryland, College Park, MD 20742 USA; 10 Departamento Química and Centro de Investigación en Síntesis Química, Universidad de La Rioja, 26006 Rioja, Spain

**Keywords:** Glycobiology, Hydrolases, Bacterial immune evasion, Structural biology

## Abstract

Bacterial pathogens have evolved intricate mechanisms to evade the human immune system, including the production of immunomodulatory enzymes. *Streptococcus pyogenes* serotypes secrete two multi-modular endo-β-*N*-acetylglucosaminidases, EndoS and EndoS2, that specifically deglycosylate the conserved *N*-glycan at Asn297 on IgG Fc, disabling antibody-mediated effector functions. Amongst thousands of known carbohydrate-active enzymes, EndoS and EndoS2 represent just a handful of enzymes that are specific to the protein portion of the glycoprotein substrate, not just the glycan component. Here, we present the cryoEM structure of EndoS in complex with the IgG1 Fc fragment. In combination with small-angle X-ray scattering, alanine scanning mutagenesis, hydrolytic activity measurements, enzyme kinetics, nuclear magnetic resonance and molecular dynamics analyses, we establish the mechanisms of recognition and specific deglycosylation of IgG antibodies by EndoS and EndoS2. Our results provide a rational basis from which to engineer novel enzymes with antibody and glycan selectivity for clinical and biotechnological applications.

## Abstract (web summary)

Bacterial pathogens have evolved intricate mechanisms to evade the human immune system, including the production of immunomodulatory enzymes. Here, the authors establish the mechanisms of recognition and specific deglycosylation of IgG antibodies by the multi-modular enzymes EndoS and EndoS2

## Introduction

**Fig. 1:**
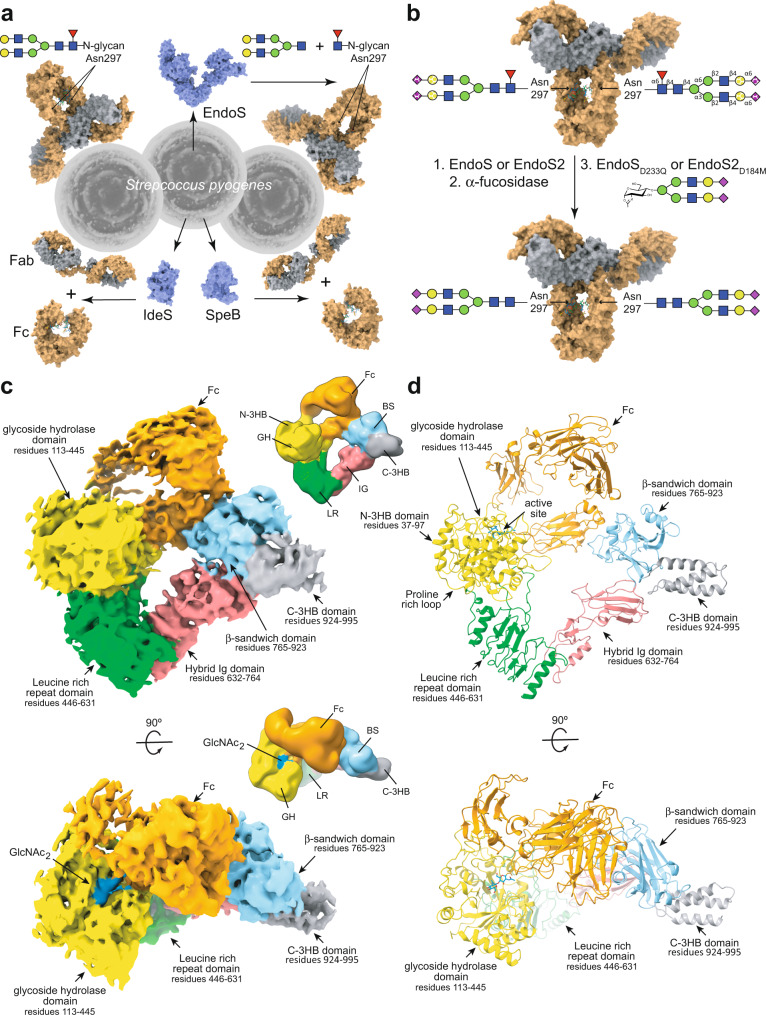
Overall structure of EndoS in complex with the Fc region. **a** EndoS, SpeB and IdeS are secreted enzymes from *S. pyogenes* that can act on IgG antibodies. SpeB and IdeS are proteases that hydrolyze the flexible hinge region between residues 220 to 248 of the IgG antibody^96–98^. EndoS is an ENGase that hydrolyze the β−1,4 linkage between the first two GlcNAcs of the CT *N*-glycans on the Fc region of IgG antibodies. The three enzymes inactivate IgG antibodies against *S. pyogenes*. **b** EndoS and EndoS2 can be used to remodel the *N*-glycan on therapeutic IgG antibodies using a chemoenzymatic approach. First, wild-type EndoS and/or EndoS2 hydrolyze a variety of glycoforms present in the Fc region according to their *N*-glycan specificity. Next, other enzymes can be used to hydrolyze specific carbohydrate moieties, e.g. fucose which absence improves the binding to FcγRIIIa and the antibody-dependent cellular cytotoxicity (ADCC) properties of the antibody. Last, EndoS_D233Q_ and EndoS2_D184M_ glycosynthase mutants facilitate the transfer of a glycan-oxazoline donor with a defined glycoform to the Fc region of IgG antibodies. The dotted shapes represent that carbohydrate may or may not be present, indicating the high heterogeneity of the *N*-glycan which impact the functionality, immunogenicity and pharmacokinetic of the antibody. **c** CryoEM maps of the EndoS_E235A_-Fc complex, including a schematic representation of the EndoS domains and the Fc region in the complex structure. **d** Structural model showing the overall fold and the secondary structure organization of the EndoS-Fc complex.

*Streptococcus pyogenes* (group A *Streptococcus*; GAS) is a Gram-positive pathogenic bacterium that causes life-threatening conditions and post-infection immune-related diseases, in addition to mild skin and upper respiratory tract infections^1^. In order to evade the immune system and facilitate the colonization of the host, GAS employs a large variety of mechanisms, most notably the secretion of enzymes that selectively inactivate key molecules of the immune system, such as immunoglobulin G (IgG) antibodies (Fig. 1a)^2^. EndoS^3^ and EndoS2^4^ are endo-β-*N*-acetylglucosaminidases (ENGases) produced by different GAS serotypes that hydrolyze the Asn297-linked *N*-glycan on the fragment crystallizable (Fc) region of IgG antibodies. EndoS is secreted by several GAS serotypes, including M1, while EndoS2 is exclusively secreted by the M49 GAS serotype^4^. These ENGases are the most prominent members of a rare class of carbohydrate-active enzymes that are specific to the protein component of the glycoprotein substrate and not just the glycan component; no mechanism of protein specificity by this class of enzymes has yet been described. The Asn297-linked *N*-glycan is required for IgG binding to Fc γ receptors (FcγRs) and complement C1q, which trigger antibody-mediated effector functions^5–8^. The activity of these enzymes thereby abolishes the effector functions mediated by the Fc region of IgG antibodies, increasing the survival and virulence of the bacteria^9^. Accordingly, IgG-specific ENGases have been shown to ameliorate autoimmune disease in diverse animal models^10–13^. In addition, EndoS and EndoS2 are powerful tools for the chemoenzymatic synthesis of antibodies with homogenous glycoforms^14–17^ and drug conjugates^18,19^. The Fc region *N*-glycans of IgG antibodies in serum are composed of >33 distinct glycoforms^20^ and their unique chemical structures modulate the effector functions by altering Fc binding to FcγRs, as well as the stability and half-life of the antibody^21^. Antibody-based therapeutics, one of the most prominent and expanding class of drugs used for the treatment of diverse human disorders, including cancer, autoimmunity, and infectious diseases^22^, are produced with heterogeneous glycoforms that impact their safety and efficacy^21^. EndoS and EndoS2 are particularly useful for Fc glycan remodeling due to the strict specificity of both enzymes for intact IgG antibodies and the discovery of their respective glycosynthase mutants that are capable of transferring a homogenous *N*-glycan to IgG antibodies using a chemoenzymatic approach^23,24^ (Fig. 1b). The generation of antibodies with defined glycoforms is critical not only to understand the role of each glycoform on antibody function but also for the production of antibodies with defined therapeutic, diagnostic and pharmacokinetic properties^25^.

EndoS and EndoS2 share 37% sequence identity and, although they are both IgG-specific, exhibit distinct *N*-glycan substrate specificities. EndoS processes exclusively complex type (CT) *N*-glycans on IgG antibodies, while EndoS2 is active on CT, high mannose (HM) type and hybrid (Hy) type glycans on IgG. EndoS is a 108 kDa multi-modular protein composed of six domains which adopt a “V”-shaped structure, from the N- to the C-terminus: (i) an N-terminal three-helix bundle domain (N-3HB; residues 37–97); (ii) a glycosidase hydrolase domain (GH; residues 98–445); (iii) a leucine-rich repeat domain (LRR; residues 446–631); (iv) a hybrid Ig domain (hIg; residues 632–764); (v) a β-sandwich domain (residues 765–923), and (vi) a C-terminal three-helix bundle domain (C-3HB; residues 924–995)^26,27^. EndoS2 is a 95 kDa multi-modular protein that shares a similar overall architecture with EndoS but lacks the two 3HB domains on its N- and C-termini^28^. We previously described the structural determinants that govern the *N*-glycan specificities of EndoS^26,27^ and EndoS2^28^. However, the mechanism by which these enzymes specifically process the *N*-glycan on the Fc region of IgG antibodies but no other glycoproteins has not yet been described. Here, we present the cryoEM structure of EndoS in complex with the IgG1 Fc region. In combination with small-angle X-ray scattering (SAXS), alanine scanning mutagenesis, hydrolytic activity measurements, nuclear magnetic resonance (NMR) and molecular dynamics (MD) analyses, we describe the molecular mechanisms of recognition and specificity by EndoS and EndoS2 for IgG antibodies. Our findings will enable novel strategies for the chemoenzymatic synthesis of monoclonal antibodies with improved therapeutic properties, as well as the engineering of enzymes to treat diseases of the adaptive immune system.

## Results

### The architecture of the EndoS-IgG1 Fc region complex

To elucidate the molecular mechanism of antibody recognition by EndoS, we determined the single-particle cryoEM structure of the catalytically inactive mutant EndoS_E233A_ in complex with the Fc region of an IgG1 monoclonal antibody (EndoS_E235A_-Fc) at 4.5 Å resolution (Fig. 1c,d; Supplementary Table 1; Supplementary Figs. 1, 2, 3 and 4; Methods; PDB code 8A64). Although the purification of the native enzyme-substrate complex by size exclusion chromatography (SEC) was heterogeneous, we were able to purify the cross-linked EndoS_E235A_-Fc using the Grafix method^29^ (Supplementary Fig. 1). The SDS-PAGE of the fractions obtained by stabilization of the EndoS_E235A_-Fc complex with mild glutaraldehyde conditions mainly shows the formation of a band corresponding to the molecular weight (MW) of the EndoS_E235A_-Fc 1:1 complex but also other bands at higher MW that could correspond with the EndoS_E235A_-Fc 2:1 (250 kDa) (Supplementary Fig. 1a). We isolated the fractions of the EndoS_E235A_-Fc 1:1 complex and further purified the sample by SEC (Supplementary Fig. 2; Methods). In the EndoS_E235A_-Fc complex structure, the Fc region is located at the cleft of the “V”-shaped structure of EndoS, between the GH and the β-sandwich domains. The Fc region is a homodimer of the C-terminal domains of the antibody heavy chain. Each protomer compromises Cγ2 and Cγ3 domains, which interact with the equivalent domains of the other protomer via disulfide bonds in the hinge region and non-covalent interactions between the Cγ3 domains. The *N*-glycan is linked to each Asn297 of both Cγ2 domains. One of the Fc protomers (FcP1) interacts with the GH and the β-sandwich domains of EndoS through an extensive contact area^30^ of ca. 874 Å^2^, while weak electron density of the cryoEM map can be ascribed to the second Fc protomer (FcP2) which does not make substantial interactions with EndoS and the N-3HB domain. However, multibody refinement and motion analysis showed that FcP2 transiently interacts with residues 403–413 of the GH domain of EndoS and the top of the N-3HB domain (Supplementary Video 1, 2 and 3). Structural comparison of the EndoS-Fc complex and unliganded EndoS showed no marked conformational changes (PDB code 6EN3; r.m.s.d. of 2.5 Å from 880 residues; Supplementary Fig. 5). The dimensions of EndoS in the EndoS_E235A_-Fc 3D reconstruction complex (123.1 Å × 75.1 Å × 60.5 Å) are similar to those found in the X-ray crystal structure of EndoS (129.2 Å × 80.3 Å × 60.8 Å), indicating that the V shape of EndoS is not appreciably distorted in the active complex. This is also supported by our SAXS data (Supplementary Table 2; Supplementary Fig. 6; Supplementary Note 1). Calculation of the relative displacement angle between the GH-LRR and hIg-β-sandwich-C-3HB domains axes of the V shape of EndoS showed that the cryoEM structure of EndoS_E235A_-Fc complex displays a hinge angle of 61.5° while the unliganded crystal structures of EndoS reported in the literature^26,27^ display a slightly higher angles of 62.4°, 62.9° and 64.2° (Supplementary Fig. 5a–d). Altogether, EndoS in complex with the Fc region exhibits a narrower cleft between the GH and β-sandwich domains than does unliganded EndoS (Supplementary Fig. 5a) due to the presence of the substrate. Although the overall structures of the unbound and bound EndoS are generally conserved, we observed variations in the relative position of the β-sandwich and C-3HB domains, which in the cryoEM complex structure are displaced towards the cleft of the V shape of EndoS (Supplementary Fig. 5e). In addition, the overall structure of FcP1 did not change after binding to EndoS (PDB code 1H3X; r.m.s.d. of 1.6 Å from 345 residues). The main structural changes occur on the C´ β-strand, the C´E loop (residues 295-300) that bears the *N*-glycan, and the BC loop (residues 264-272) within the Cγ2 domain of FcP1 (Fig. 1c,d and Supplementary Fig. 6).

### The EndoS GH domain interacts with the *N*-glycan and the Cγ2 domain of the Fc region

**Fig. 2:**
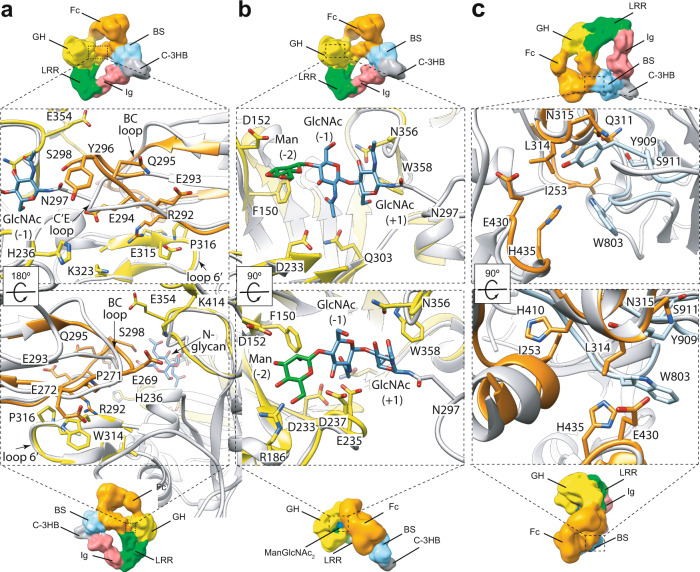
The Fc binding site of EndoS. In order to have insights on the possible location of the side chain residues that could be involved in the enzyme-substrate interactions, we locally compared/superimposed the high-resolution EndoS (PDB code 6EN3; yellow) and Fc region (PDB code 2DTS; orange) crystal structures with our EndoS_E235A_-Fc cryoEM structure (gray; Methods section). Therefore, we visualize potential side chain interactions between residues that are within interaction distance based on flexible fitting of the superimposed crystal structures. Two views of the interaction interface between the GH domain of EndoS (yellow, PDB code 6EN3) and FcP1 of the Fc region (orange, PDB code 2DTS) (**a**), the *N*-glycan (blue and green) of the Fc region and the active site of EndoS (yellow, PDB code 4NUY) (**b**) and the Cγ2-Cγ3 joint region of the Fc (orange, PDB code 2DTS) and the β-sandwich domain of EndoS (blue, PDB code 4NUY) (**c**).

It is worth noting that the resolution of the EndoS_E235A_-Fc complex, in which all the side-chains were removed, limits the definition of the interaction between the EndoS and the Fc at the atomic level of detail. In order to have insights on the possible location of the side chain residues that could be involved in the enzyme-substrate interactions, we locally compared/superimposed the high-resolution EndoS (PDB code 6EN3) and Fc region (PDB code 2DTS) crystal structures with our EndoS_E235A_-Fc cryoEM structure (Fig. 2; Supplementary Fig. 5; Methods section). Therefore, we will discuss throughout the text potential side chain interactions between residues that are within interaction distance based on flexible fitting of the superimposed crystal structures.

We have previously shown that the CT *N*-glycan product is accommodated in the binding site of the GH domain of EndoS formed by several loops whose conformations govern the strict substrate specificity of the enzyme^27^ (PDB code 6EN3). The EndoS GH domain adopts a conserved (β/α)_8_-barrel with a defined *N*-glycan binding site delineated by the N-3HB domain and the connecting loops β1–β2 (loop 1; residues 120–145), β2–α4 (loop 2; residues 151–158), β3–α5 (loop 3; residues 185–206), β4–α6 (loop 4; residues 235–247), β5–α7 (loop 5; residues 281–289), β6–α8 (loop 6; residues 304–306), β9–α10 (loop 7; residues 347–380), β10–α11 (loop 8; residues 403–413), and α11–α12 (loop 9; residues 420–434). The cryoEM EndoS_E235A_-Fc structure shows that loop 6´ (residues 312-324) of the GH domain interacts with the C´ β-strand and the BC loop of the Cγ2 of FcP1 (Fig. 2). This loop adopts a β-hairpin architecture, which is unique amongst GH18 enzyme structures, and creates an opening towards the active site of the EndoS GH domain^26^. In contrast, EndoS2 exhibits a shorter loop at this position comprising different amino acid residues^28^ (Supplementary Fig. 7). In addition, both loops 4 and 7 of the EndoS GH domain also interact with C´E loop of the Fc region. These interactions between FcP1 and the GH domain of EndoS cause a distortion of the C´ β-strand and conformational changes in the C´E loop. This leads to the conformational change of the C´E loop carrying the *N*-glycan and the side chain rotamer of N297 that promotes the displacement of the *N*-glycan from the center of the Fc region towards the active site of the enzyme (Fig. 2a,b). The CT *N*-glycan at position N297 of the Fc region can be composed of fucosylated G0, G1 or G2 glycoforms^31^. Careful inspection of the electron density map in the active site allowed us to model the first two GlcNAc residues and the central mannose core saccharide (Supplementary Fig. 8). The trisaccharide interacts with residues of loops 2 and 7 of EndoS in our cryoEM EndoS_E235A_-Fc structure.

### The EndoS β-sandwich domain confers IgG specificity through a protein-protein interaction with the Fc region

The β-sandwich domain of EndoS is composed of eight β-strands connected by short loops and arranged in two opposing antiparallel β-sheets, including β1-β2-β7-β4-β5, and β6-β3-β8 (Fig. 2c). Both EndoS and EndoS2 β-sandwich domains were previously classified and suggested to function as carbohydrate-binding modules (CBM) from family 32 based on structural homology^26–28,32^ and identified as essential for enzymatic activity^26,28,33^. However, the cryoEM EndoS_E235A_-Fc structure clearly reveals that the loops of the β-sandwich domain bind to FcP1 through protein-protein interactions. Moreover, N297 that bears the *N*-glycan points towards the GH domain and in the opposite direction of the β-sandwich domain. The β-sandwich domain interacts exclusively with the Cγ2-Cγ3 joint of FcP1. Specifically, the EndoS residue W803, previously identified as a key residue for EndoS hydrolytic activity and binding to IgG1 Fc^26^, and Y909 of EndoS are located in this interaction area between Cγ2 and Cγ3 domain. In addition, W803 together with S800, Y909, S910 and S911 were proposed to form a putative *N*-glycan binding pocket^28^ but the EndoS_E235A_-Fc cryoEM structure suggests that the main function of W803 and Y909 is to mediate protein-protein interactions between the enzyme and the Fc region of the substrate. Structural comparison of the EndoS_E235A_-Fc complex with that of EndoS2 supports a common binding mode to Fc for both enzymes. EndoS2 also bears aromatic residues, W712 and Y820, at equivalent positions to W803 and Y909 in EndoS, respectively, which are indispensable for substrate binding and catalysis^28^ (Supplementary Fig. 7). Thus, the β-sandwich domains of EndoS and EndoS2, previously proposed to be CBMs, act instead to mediate a protein-protein interface with its glycoprotein substrate in which no glycan participates.

### EndoS-Fc protein-protein interactions are critical for glycoside hydrolase activity

**Fig. 3:**
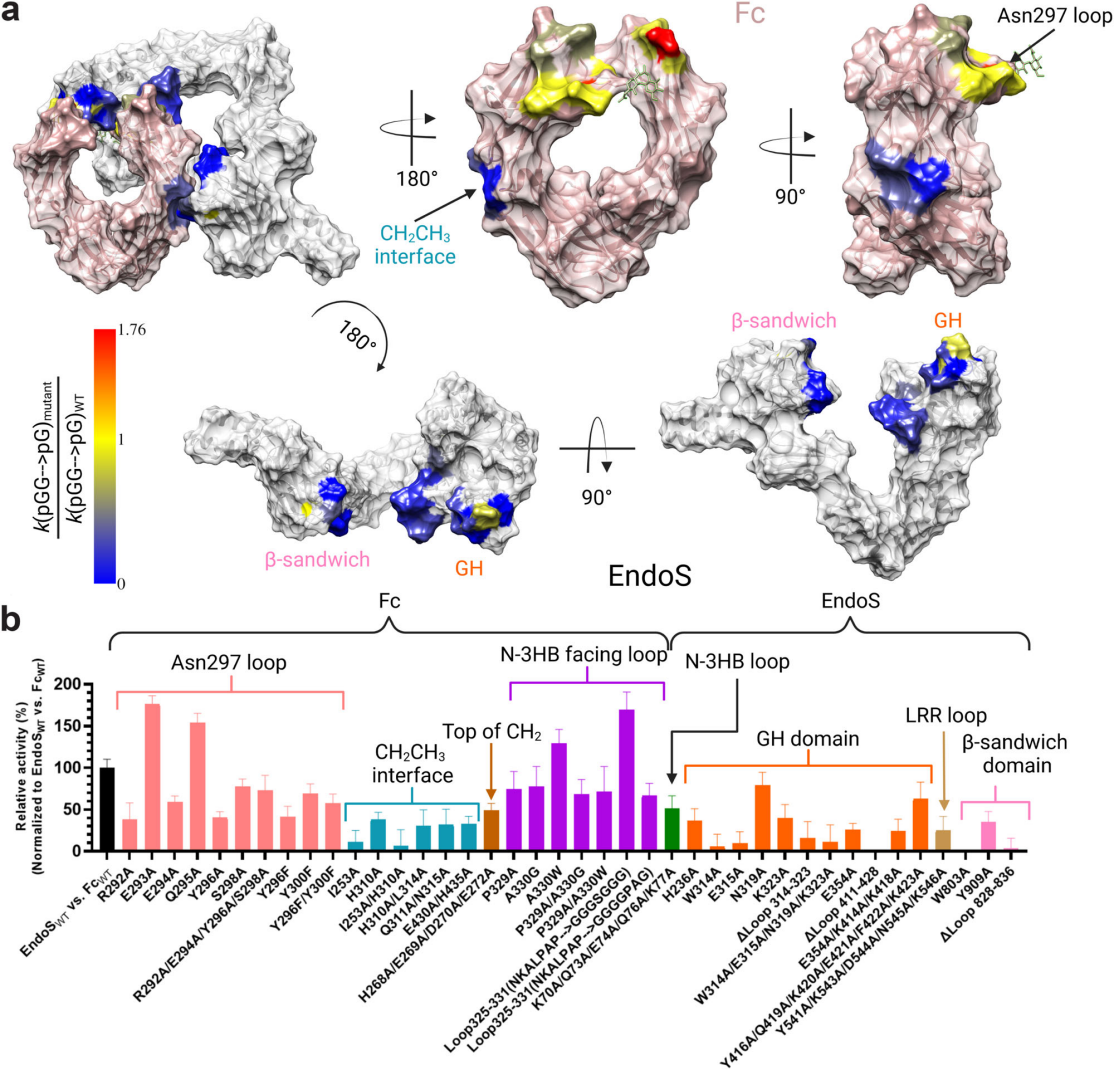
Alanine scanning mutagenesis of the EndoS-Fc interface. **a** Structure of the EndoS (gray) complex with Fc (pink) in which the regions with alanine mutants are color coded based on the relative rates of removal of the *N*-glycans from Fc alanine mutants by EndoS and vice versa. The rates were measured via LC-MS kinetic analyses. Red indicates the largest increase in hydrolytic activity while blue indicates no hydrolytic activity. **b** Rates of removal of the first glycan of Fc and Fc alanine mutants by EndoS alanine mutants and EndoS respectively, normalized to EndoS vs. Fc. The kinetic rates were derived using three independent experiments. Data is presented as mean values + /- SD. All rates were statistically significant (multiple comparison test, one-way ANOVA, Tukey method, p < 0.0001 with 95% CI).

To further investigate the molecular determinants of EndoS-Fc complex formation, we performed single and multiple alanine mutations in both EndoS and Fc, as well as loop deletions in EndoS to determine the key residues affecting hydrolytic activity in the EndoS-Fc interface. The residues in EndoS and Fc were selected based on the superposition of the high-resolution crystal structures of EndoS (PDB code 6EN3) and Fc region (PDB code 2DTS) with the EndoS_E235A_-Fc cryoEM structure. We determined the rates by which the EndoS mutants hydrolyzed glycans from Fc and by which EndoS hydrolyzed glycans from Fc mutants by tracking the proportions of glycosylated versus deglycosylated Fc over time by intact mass spectrometry (Fig. 3; Supplementary Figs. 9 and 10). Specifically, in the Fc region we mutated residues of the C´E loop of the Cγ2 domain (residues R292, E293, E294, Q295, Y296 and S298), the Cγ2-Cγ3 joint region (residues I253, H310, Q311, L314, N315, E430 and H435), the BC loop of Cγ2 domain (H268, E269, D270, E272), and the FG loop of the Cγ2 domain (residues 325-331) that faces the N-3HB domain of EndoS to alanine. For positions in this loop that are encoded by alanine residues in the Fc region, we made mutations to glycine and tryptophan in order to determine the effect of removing or extending the side chains at those positions, respectively. In EndoS, we mutated residues in the N-3HB loop (residues K70, Q73, E74, Q76 and K77), the GH domain (residues H236, W314, E315, N319, K323, E354, K414, Y416, K418, Q319, K420, E421, F422, K423 and loops 6' and 9), the LRR loop (residues Y541, K543, D544, N545 and K546) and the β-sandwich domain (residues W803, Y909 and loop 828-836) to alanine and assessed their hydrolytic activities. As shown in Fig. 3, mutations in the C´E loop exhibited pleiotropic effects on hydrolytic activity; mutations in residues E293A and Q295A that did not interact with the enzyme, resulted in faster deglycosylation by EndoS, while the remaining mutations reduced the rate of glycan hydrolysis. Similar results were obtained for mutations in the N-3HB facing loop on the Fc, with some variants (e.g., A330W and NKALPAP→GGGSGGG) resulting in faster glycan removal while the remainder reduced the rate of *N*-glycan hydrolysis. Mutations in the Cγ2-Cγ3 joint residues that interact with the β-sandwich domain of EndoS significantly reduced the rate of glycan hydrolysis. In EndoS, mutations in the N-3HB, GH, LRR and β-sandwich domains all significantly reduced the rate of glycan hydrolysis from Fc, with the removal of loop 9 in the GH domain and the W803A mutation in the β-sandwich domain completely abolished EndoS activity.

The results of our mutational analysis indicate that the most important region driving EndoS-Fc complex formation and *N*-glycan hydrolysis involves residues on the Cγ2-Cγ3 joint region of the Fc which interact with the β-sandwich domain of EndoS. The most extreme examples are (i) the EndoS W803A mutation and (ii) the Fc I253A/H310A double mutation, which completely abolish hydrolytic activity (Fig. 3). The variable activities observed in the Asn297-loop and N-3HB facing loops in Fc and in the N-3HB in EndoS suggest that conformations adopted by these loops play an important role in recognition of Fc by EndoS. Mutations in the EndoS GH domain significantly reduced enzymatic activity suggesting that these mutations adversely affect the ability of the *N*-glycan to enter the active site. The reduction of the hydrolytic activity observed for mutations in the LRR loop of EndoS suggests that these mutations may adversely affect the conformation of this loop which stabilizes the conformation of the GH domain loop 6' to effectively recognize the glycan loop of the antibody as shown by the EndoS_E235A_-Fc cryoEM structure^27^.

**Fig. 4:**
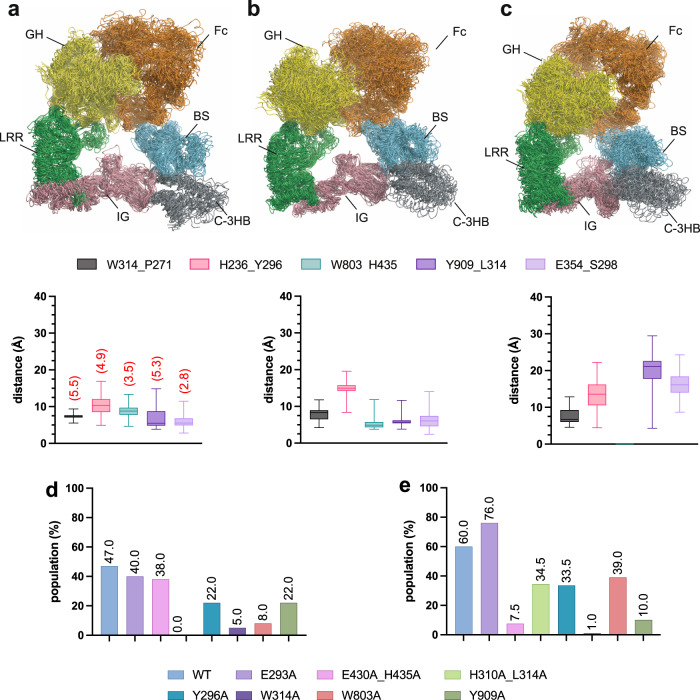
Molecular dynamics (MD) simulations studies. Overlay of 25 frames evenly spaced along the 1 µs MD trajectory for (**a**) EndoS-Fc, (**b**) EndoS-Fc_E293A_ (**c**) EndoS_W803A-Fc_, along with the average distance between representative residues. The EndoS protein is shown as blue ribbons and the antibody as gray and orange ribbons. Box-and-whisker plots, showing the minimum, maximum, median, 1st quartile, and 3rd quartile bars, as well as the interquartile range, based on *n* = 250 evenly distributed values obtained from the MD trajectories. The numbers in red and in parentheses are the experimental values found in the cryo-EM structure. For aromatic residues and Pro, the center of the aromatic ring and the center of the ring are considered, respectively, for these calculations. For L314, S298 and E354 residues, the Cδ, the OH group, and the oxygens of the carboxylate are considered, respectively, for the calculations (cut-off distance: 5.5 Å –π-π stating and CH-π – and 3.5 Å –hydrogen bond–). Errors are given as SD. The numbers in red and in parentheses are the experimental values found in the cryo-EM structure. The dashed lines represent the distance values found in the cryo-EM structure of EndoS-Fc. **d** Population of the CH-π interaction between Y909 (EndoS) and L314 (Fc) derived from the MD simulations. **e** Population of salt-bridge K323 (EndoS) and E294 (Fc) derived from the MD simulations. The side chain nitrogen of Lys and the carbon atom of the guanidino group of Arg are considered for these calculations. (cut-off distance: 4.5 Å).

In order to complement our structural and biochemical experimental data, we further evaluated the conformational behavior of the IgG1 Fc region by molecular dynamics (MD) simulations. We modeled the structure of wildtype and mutant EndoS-Fc complex structures, including key mutations in the Fc region (E293A, Y296A, E430A-H435A, and H310A-L314A) or in EndoS (W314A, W803A, and Y909A) corresponding to select mutations in the hydrolytic activity experiments described above. MD simulations performed for the EndoS-Fc complex show that the predicted CH-π interactions between the protein and the antibody, along with the predicted π-π stacking interactions involving W803 of EndoS and H435 of Fc, are sparsely populated in solution, with populations <3% in all cases. The predicted hydrogen bond between the side chains of E354 and S298 and the salt-bridge between E315 of the protein and R292 of Fc are also very low populated (≈2%). A similar scenario was observed for the other complexes modeled in this work (Fig. 4b-c and Supplementary Figs. 12 and 13). Notably, for EndoS-Fc complex, the predicted CH-π interaction between Y909 and L314 and the salt-bridge between K323 of EndoS and E294 of Fc (Fig. 2), are formed in 47% and 60% of the total trajectory time, respectively (Fig. 4a,d,e and Supplementary Figs. 12 and 13). Of note, the number and population of stabilizing interactions observed for the complex with the FcP1 E293A mutation is higher than in the wild-type complex (Fig. 4b,d,e and Supplementary Figs. 12 and 13). In fact, the predicted salt-bridge (K323-E294) is occupied ca. 76% for this mutant in solution. Additionally, the predicted salt-bridge (E315-R292) and the π-π stacking interactions involving W803 of EndoS and H435 of Fc, which are not populated in the wild-type complex, have an occupancy close to 82% and 70%, respectively in this mutant. Thus, this result derived from the MD simulations could explain/support, at least in part, the high activity of this mutant. The absence of these predicted interactions in the rest of the complexes can lead, in some cases, to a change in their 3D structure. This change is evident when the wildtype is compared with, for example, EndoS-Fc_H310A/L314A_, in which part of the antibody does not interact with the protein (Supplementary Fig. 11). On the other hand, slight differences were observed between the different complexes in the r.m.s.d. values of several loops and domains of the complexes (Supplementary Fig. 14 and 15).

### EndoS and EndoS2 deglycosylate the two glycans of the IgG Fc homodimer sequentially

**Fig. 5:**
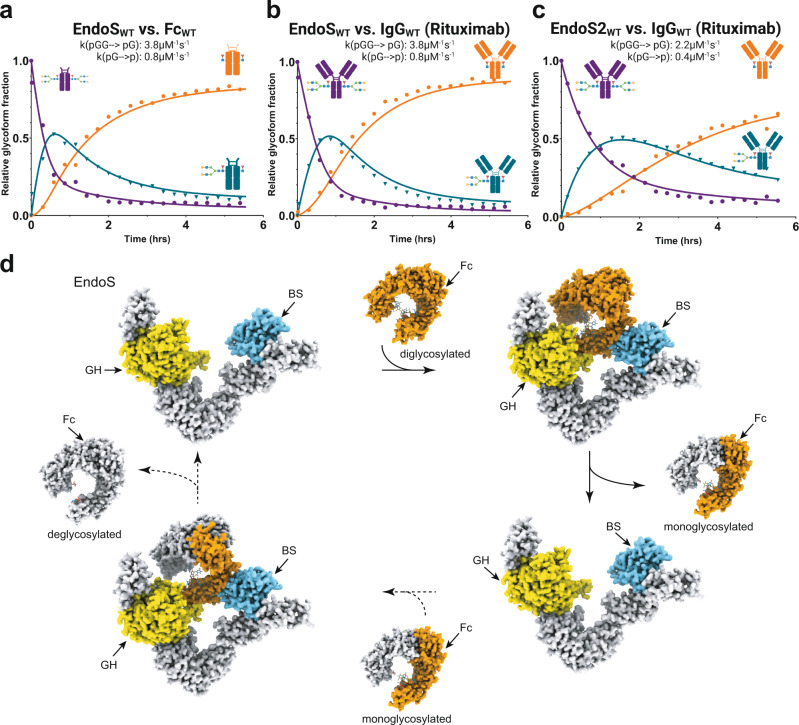
Molecular mechanism of antibody recognition by EndoS and EndoS2. Kinetic modeling of deglycosylation of (**a**) Fc_WT_ and (**b**) IgG_WT_ by EndoS_WT_ and (**c**) IgG_WT_ by EndoS2_WT_. LC-MS kinetic analysis of deglycosylation of Fc_WT_ and IgG_WT_ by EndoS_WT_ showing the hydrolysis over time of the two *N*-glycans linked to Asn297. The deglycosylation process occurs sequentially as seen by the initial increase then decrease in monoglycosylated population (dark green) over time while the diglycosylated (purple) and deglycosylated (orange) species increase over time. **d** Schematic representation of Fc *N*-glycan processing by EndoS. In a first step, EndoS binds the diglycosylated Fc and hydrolyzes one of the *N*-glycan of the Fc which causes the release of monoglycosylated Fc. In a second step, EndoS engages the monoglycosylated Fc and hydrolyzes the remaining *N*-glycan of the Fc to produced completely deglycosylated Fc.

Our EndoS_E235A_-Fc cryoEM structure revealed a 1:1 EndoS:Fc complex in which EndoS engages a single Fc protomer through a protein-protein interaction formed by the EndoS β-sandwich domain and the Fc Cγ2-Cγ3 joint, while the EndoS GH domain engages the Asn297-linked glycan on the same Fc protomer. However, EndoS hydrolyzes the glycans from both Fc protomers and no evidence of partially deglycosylated IgG has been reported to date. In order to define the mechanism of full deglycosylation of IgG by EndoS, we analyzed the deglycosylation reaction over time. We mixed wild-type EndoS with Fc in a 1:5000 enzyme:substrate ratio and analyzed changes in intact Fc masses by LC-MS approximately every 5 min (Fig. 5a). As expected, the di-glycosylated Fc species decreased while the fully deglycosylated species increased over time. As the reaction proceeded, however, we observed that a mono-glycosylated species initially increased to ~50 percent of all Fc masses, prior to decreasing along with di-glycosylated Fc to near zero. From these data, we calculated a rate of hydrolysis for the transition from di-glycosylated Fc to mono-glycosylated Fc of 3.8 μM^−1^s^−1^ and for the transition from mono-glycosylated Fc to fully deglycosylated Fc of 0.8 μM^−1^s^−1^. Thus, the hydrolysis of the first glycan was ~5-fold faster than the hydrolysis of the second glycan. The different EndoS reaction rates found in the first and second reaction steps could be caused by a substrate concentration effect. In the first step, the Fc has two points of glycosylation that can be hydrolyzed while in the second step the Fc has only one remaining point of glycosylation to be removed by EndoS. However, it has been demonstrated that the dynamic quaternary structure of IgG1-Fc is significantly altered by the removal of the *N*-glycans based on SAXS experiments and molecular dynamics simulations^34,35^. Therefore, other structural factors (e.g. asymmetry in the mono-glycosylated Fc conformation) might contribute to interpreting differences in the reaction rates. We conducted an analogous experiment with EndoS and Rituximab, a full-length IgG1 antibody and observed identical kinetic parameters for hydrolysis of the first and second glycans (Fig. 5b). This data suggest that the Fab arms of the antibody have no contribution to the interaction with EndoS. We also conducted an analogous experiment with EndoS2 and Rituximab and found similar kinetic behavior (Fig. 5c); although each of the reaction rates was slower, hydrolysis of the first glycan was also 5-fold faster than that of the second glycan, as for EndoS. These data indicate that EndoS- and EndoS2-mediated hydrolysis of the two glycans on Fc or IgG occurs sequentially, concomitant with dissociation of the enzyme-substrate complex after hydrolysis of the first glycan before re-associating to hydrolyze the second glycan.

### The sterically constrained glycan binding site of EndoS2 prevents deglycosylation of potential non-IgG glycoprotein substrates

To shed more light on the interaction of EndoS and EndoS2 with their natural ligands, we employed NMR chemical shift perturbations (CSPs)^36^. Generally speaking, the method consisted of following changes in chemical shifts from signals in NMR spectra during ligand titrations and using them to determine the location of binding sites, affinity of ligands and/or conformational equilibria. We used CSPs for the quantification of the overall thermodynamic and kinetic binding parameters of EndoS2 to IgG1 Fc and its reaction products. We extracted CSPs from methyl-TROSY NMR spectra^37^ and fitted quantum mechanically simulated spectra using the TITAN (TITration ANalysis) software package^38^. This approach offers unique access to binding thermodynamic and kinetic parameters^37,39^, providing a link to the conformational changes observed by crystallography, H/D exchange MS^28^, cryo-EM and MD, and assisting our mechanistic understanding of EndoS2 enzymatic activity (Supplementary Note 2: Supplementary Figs. 15 to 33; Methods).

**Fig. 6:**
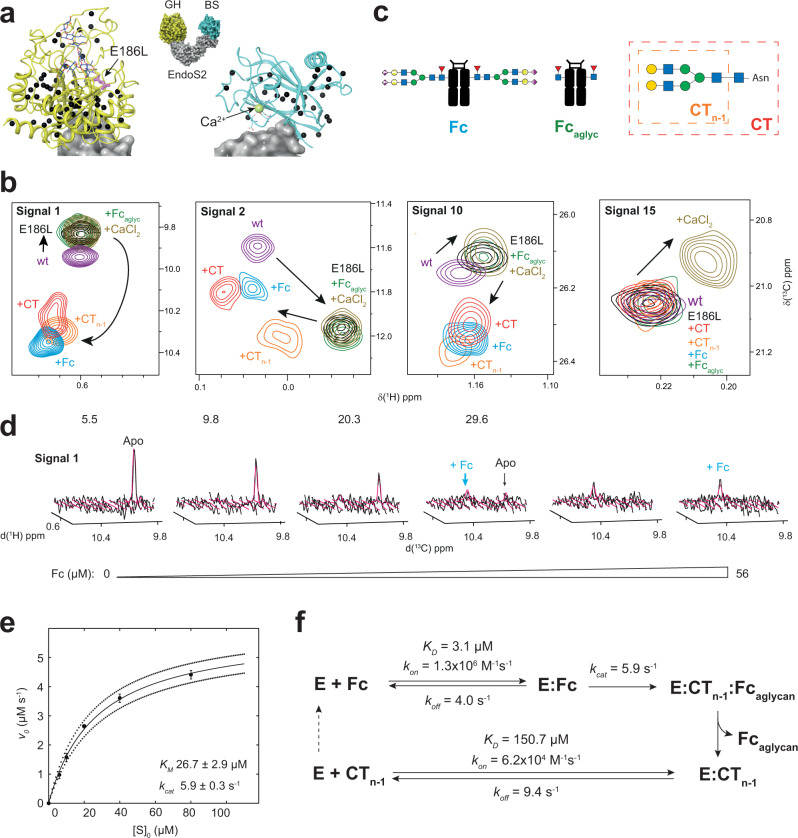
Binding affinity and enzyme kinetics experiments studied by NMR. **a** Crystal structure of EndoS2 bound to CT *N*-glycan and Ca^2+^ (PDB code 6MDS) showing the location of the [^13^C,^1^H_3_]-methyl labeled probes as black spheres. Glycoside hydrolase (GH) and β-sandwich (BS) domains are colored in yellow and pale blue, respectively. Mutated amino acid E186L, CT glycan and Ca^2+^ ion are depicted in pink, dark blue and pale green. **b** Superimposition of selected regions of methyl-TROSY spectra showing CSPs upon mutagenesis or at saturation concentrations of different ligands. Color code corresponds to: EndoS2 (violet), EndoS2_E186L_ in the absence (black) and in the presence of CT (red), CT_n-1_ (orange), IgG1 Fc (blue) and Ca^2+^ (yellow). Addition of Fc_aglyc_ (green) induced to CSPs, suggesting very weak protein-protein interactions. Arrows indicate the extent of CSPs observed between the different protein states. **c** Ligands used for NMR titration experiments. IgG1 Fc region (Fc), complex-type carbohydrate (CT) and the products of glycan hydrolysis Fc_aglyc_ and CT_n−1_, respectively. Color code like in Fig. 1. **d** Quantum mechanics-based 2D lineshape fitting of methyl-TROSY spectra of a representative signal for the titration of EndoS2_E186L_ with Fc. Experimental and fitted spectra are shown as black and pink surfaces, respectively. Inserts correspond to 0, 5.5, 9.8, 20.3, 29.6 and 56 µM Fc concentrations. **e** [S]_0_-dependence of the initial enzyme cycling velocity. The solid line represents the best-fit to Michaelis Menten Eq. 4, and dotted lines correspond to one standard deviation. Data are presented as mean, and errors correspond to standard deviation. **f** Schematic mechanism of Fc *N*-glycan processing by EndoS2 as determined from lineshape analysis. The affinity of EndoS2 (here E) for Fc is two orders of magnitude higher as compared to the reaction product CT_n−1_, promoting substrate depletion. On-rates are responsible for increased affinity towards Fc as compared to CT_n−1_, suggesting differences in the initial enzyme-ligand encounter event. Values correspond to (**f**) and Table 1. All experiments were acquired at 600 MHz and 298 K.

While we were unable to express EndoS in minimal media in quantities required for NMR experiments, we were able to express and purify isotopically [*U*-^15^N,^2^H] MIL^proS^V^proS^ [^13^C,^1^H_3_]-methyl labeled^39–41^ EndoS2 and catalytically inactive EndoS2_E186L_ in order to perform these experiments. This labeling scheme produced methyl-TROSY spectra with excellent signal-to-noise (Supplementary Fig. 16). In addition, selected methyl probes were well distributed around the binding pockets, allowing for the extraction of CSPs upon ligand titration (Fig. 6a,b). We measured the binding kinetics of EndoS2_E186L_ to its CT glycan substrate, the IgG1 Fc region, the reaction products Fc aglycan and CT_n-1_, and Ca^2+^ ions (Fig. 6c). We also studied the interaction of catalytically inactive EndoBT-3987_D312A/E314L_, a GH18 family ENGase that hydrolyzes HM glycans from myriad glycoproteins but is not specific for IgG antibodies or any other protein (Supplementary Fig. 17)^42,43^. Quantitative line shape analysis of methyl-TROSY spectra showed that EndoS2_D186L_ binds Rituximab-Fc with a dissociation constant of 3.1 µM (Table 1, entry 1), similar to the previously reported *K*_*D*_ of ∼9 μM for the binding of EndoS2_D186L_ to IgG1-CT obtained by surface plasmon resonance^28^. A representative example of line shape fitting can be seen in Fig. 6d. We measured the binding affinity of EndoBT-3987_D312A/E314L_ to its HM *N*-glycan substrate to be *K*_*D*_ = 1.7 µM (Table 1, entry 2). We measured the binding affinities of EndoS2_D186L_ for soluble CT glycan and the reaction product CT_n-1_ to be 106.9 µM and 150.7 µM, respectively, two orders of magnitude weaker than the EndoS2_E186L_-Fc and EndoBT-3987_D312A/E314L_ affinities. We measured on-rate constants for Fc, CT and CT_n-1_ to be 1.3 × 10^6 ^M^−1^s^−1^, 1.1 × 10^5 ^M^−1^s^−1^ and 6.2 × 10^4 ^M^−1^s^−1^, respectively, all much slower than expected for a diffusion-controlled process, indicating that these interactions involve solvent reorientation and possibly conformational changes. Dissociation rate constants *k*_*off*_ were similar for these three ligands, and go down into the range of a few Hz (Table 1, entries 3,4). The faster on-rate constant observed for the interaction of EndoS2_D186L_ with Fc, similar to the on-rate constant of EndoBT_D312A/E314_ for just the *N*-glycan, suggests that a protein-protein interaction favors a “correct” orientation of the EndoS2 GH domain relative to the glycan during the initial EndoS2-Fc fragment encounter event. The energetic contribution of such interactions can be approximated as the change in the difference of the standard free energy $\triangle \triangle {G}_{{Fc}-{CT}}^{^\circ }=\triangle {G}_{{FC}}^{^\circ }-\triangle {G}_{{CT}}^{^\circ }=$ -8.7 kJ, considering $\triangle {G}^{^\circ }={RT}{{\mathrm{ln}}}({K}_{D})$. We found that EndoS2-mediated hydrolysis of Fc *N*-glycans abrogated recognition of Fc by EndoS2_D186L_ at otherwise saturating Fc concentrations, highlighting the central role of the carbohydrate for the recognition of IgG by EndoS2 and preventing re-binding of reaction products (Table 1, entry 5). We also extracted the catalytic rate, *k*_*cat*_, and the Michaelis-Menten constant, *K*_M_, from the catalytic cleavage of CT *N*-glycans from the Fc region by EndoS2 by measuring the concentration of enzymatically released glycans by ^1^H NMR spectra (Supplementary Note 2; Methods). Under the conditions used in this study, we observed a catalytic rate *k*_*cat*_ of 5.9 s^−1^ and a Michaelis-Menten constant *K*_M_ of 26.7 µM (Fig. 6e). Combined with line shape analysis, these results provide a first comprehensive picture of the catalytic mechanics of the enzymatic processing of IgG1 Fc *N*-glycans by EndoS2 (Fig. 6f).

**Table 1:** Binding affinity of EndoS2 for IgG antibodies and *N*-glycans

N°	Enzyme	Ligand	*K*_*D*_ (µM)	*k*_*off*_ (s^−1^)	*k*_*on*_ (M^−1^s^−1^)
1	EndoS2_E186L_	Fc	3.1 ± 0.6	4.0 ± 1.5	1.3 × 10^6^ ± 5.5 × 10^5^
2	EndoBT-3987_D312A/E314L_	HM	1.7 ± 0.9	5.5 ± 1.4	3.2 × 10^6^ ± 1.9 × 10^5^
3	EndoS2_E186L_	CT	106.9 ± 6.7	11.4 ± 1.5	1.1 × 10^5^ ± 1.6 × 10^4^
4	EndoS2_E186L_	CT_n−1_	150.7 ± 18.0	9.4 ± 3.7	6.2 × 10^4^ ± 2.6 × 10^4^
5	EndoS2_E186L_	Fc_aglyc_	–	–	–
6	EndoS2_E186L_	CaCl_2_	3415 ± 480	–	–
7	EndoS2	CaCl_2_	4000 ± 100	–	–

Dissociation constants *K*_*D*_, on- and off-rate constants *k*_*on*_ and *k*_*off*_ for Rituximab-Fc, CT, CT_n-1_ HM and Ca^2+^ binding to EndoS2_E186L_, EndoS2 and EndoBT-3987_D312A/E314L_ from fitting a two-state binding model to methyl-TROSY titration data using TITAN algorithm.

## Discussion

EndoS and EndoS2 are the most prominent members of a rare subset of carbohydrate-active enzymes that are specific not only to the glycan type but also to the protein component of the glycoprotein from which they hydrolyze glycans. To date, no molecular mechanism has been described to explain how these enzymes are protein-specific. Our data now provide the mechanism by which EndoS and EndoS2 hydrolyze glycans from IgG antibodies in a highly specific manner. This mechanism for protein-specific deglycosylation by EndoS and EndoS2 relies on four principles. These enzymes: (i) avoid all other potential non-IgG glycoprotein substrates; (ii) form a protein-protein interaction with a non-enzymatic domain in order to create an anchor point on the IgG substrate; (iii) leverage their unique geometry to reach from the anchor point to orient the active site of the GH domain directly adjacent to the Asn297-linked glycan that they hydrolyze; and (iv) hydrolyze the two glycans on the IgG Fc region homodimer sequentially by a catch-and-release process.

The first mechanistic principle, reduced distraction by potential non-IgG substrates, is accomplished by EndoS and EndoS2 constructing conformationally constrained glycan binding sites. This distinctive morphological property of their active sites results in the bound glycan needing to adopt a conformation in which the branches of a biantennary glycan are nearly parallel to one another^26–28^. Although such a glycan conformation is commonly rendered schematically, it is exceedingly rare in nature due to the high degree of conformational flexibility inherent to *N*-linked glycans. Even when bound in the glycan binding sites of endoglycosidases that are not protein-specific, *N*-linked glycans adopt diverse structures that are decidedly unlike the parallel-branched glycans in EndoS and EndoS2. This is the case for the *N*-linked glycans that are the substrates for EndoBT-3987^42,43^, EndoF_3_^44^, as well as many others (Supplementary Fig. 35). The adoption of the constrained glycan conformation in EndoS and EndoS2, as well as the flexible glycan conformation in EndoBT-3987, can be observed in the relative on-rates of substrates binding to catalytically inactive variants of these endoglycosidases. Indeed, our NMR data indicate that the on-rates for glycans alone are two orders of magnitude slower for EndoS and EndoS2 than they are for EndoBT-3987. However, these enzymes have all evolved to have comparable affinities and binding kinetics to their biologically relevant carbohydrates—linked to IgG for EndoS and EndoS2, while free in solution or exposed on the surface of any protein for EndoBT-3987 – a property clearly related to the distinct structural features of the active sites of these endoglycosidases and the relative entropic costs of their glycan substrates adopting their bound conformations.

The second mechanistic principle, the establishment of an enzyme-substrate protein-protein interaction, is fulfilled by the β-sandwich domain of EndoS and EndoS2. While these domains have high structural similarity to CBMs, they are not, in fact, bona fide CBMs in that they do not bind carbohydrates. Instead, these β-sandwich domains recognize a strictly protein-based molecular surface on the IgG Fc region at the joint between its Cγ2 and Cγ3 domains. This Fc surface is a common binding spot for myriad proteins, including Protein A^45^ and Protein G^46^ from bacteria, as well as the human neonatal Fc receptor^47^ (FcRn). Structural comparison of Protein A, Protein G and FcRn with EndoS structures in complex with IgG1 Fc (Supplementary Fig. 36) revealed/suggested that residues I253, H310, Q311 and H435 of the Fc region are involved in the interaction with EndoS, FcRn and Protein A, while Protein G interacts also with I253 of the Fc region, and the main interactions occur on the Cγ2 domain. Moreover, a hydrophobic residue (W803 in EndoS, F111 in Protein A, W43 in Protein G and W131 in FcRn) possibly interacts with I253 of IgG1 Fc (Supplementary Fig. 36). Protein A, Protein G and FcRn display a pH-dependent interaction with the Fc region of antibodies, binding preferentially to the Fc region at pH 8^48^, 4–5^49^ and 6^50^, respectively. IgG histidine residues located at the junction between the Cγ2 and Cγ3 domains (H310 and H433) have been shown to contribute to this pH-dependent binding^50–53^. Although the conserved interaction region between these proteins and EndoS could indicate that the binding between the β-sandwich domain and the Fc region could be also influenced by the pH, it has been shown that EndoS digests the *N*-glycan of IgGs at pH 5 and 8^54^. Moreover, the structural similarity of Cγ2 and Cγ3 joint region on the Fc region amongst all IgG subtypes of both human and mouse antibodies (Supplementary Fig. 37) explains why these glycoproteins comprise all the known substrates for EndoS and EndoS2. However, EndoS shows subtle differences in activity against each human and murine IgG subtypes, IgG1 is EndoS preferred substrate for human^26^ and IgG1 and IgG2b for mouse^55^. Our structural comparison analysis of human and murine IgG subtypes (Supplementary Fig. 37) suggests that although all IgG subtypes share a conserved Cγ2-Cγ3 joint region, the C´E loop bearing the *N*-glycan shows small conformational differences between each IgG subtype that could affect the placement of the *N*-glycan into the active site of the enzyme, leading to the observed decreased activity of EndoS against other IgG subtypes other than human IgG1 and murine IgG1 and IgG2b^26,55^. The β-sandwich and Cγ2-Cγ3 joint protein-protein interaction is absolutely required for enzymatic activity, as mutations on either side of this interface can completely abrogate hydrolysis of Asn297-linked Fc glycans by EndoS^26^ and EndoS2^28^. Moreover, the protein-protein interaction between the GH domain and the C´E loop and C´ β-strand of the Fc region fine-tunes the binding of the enzyme to the substrate, given that the alanine mutations on this area moderately decreased the activity of the enzyme. Accordingly, for EndoS and EndoS2, a protein-protein interaction drives hydrolytic activity of these endoglycosidases, a property not previously observed in carbohydrate-active enzymes.

The third mechanistic principle, orientation of the enzyme active site adjacent to the substrate glycan, is a result of the unique V-shaped molecular architecture of EndoS and EndoS2 in which the β-sandwich and GH domains are situated on either tip of the V, interspersed by an Ig-like domain and a leucine-rich repeat. As a consequence of anchoring the enzyme on the substrate via the β-sandwich domain/Fc region protein-protein interface described above and the architectures of these multi-domain enzymes, the active sites of EndoS and EndoS2 become ideally positioned for engagement with and hydrolysis of the Fc glycan. The predominant conformation of the IgG Fc region is one in which the Asn297-linked glycans are pointed inward towards the center of the homodimer, although these antibodies are known to exhibit many conformations, including those in which these glycans are more exposed to solvent^56,57^. Our cryoEM structure shows that the Fc loop on which the Asn297-linked glycan adopts just such a conformation; it is not exposed to solvent in the complex, though, but rather bound in the EndoS active site. Thus, when anchored by the enzyme-substrate protein-protein interface, the distinctive molecular architecture positions the GH domain to stabilize an otherwise infrequently populated conformation amongst the entire ensemble of Fc conformations to increase the sampling of its glycan, resulting in efficient glycan hydrolysis. It must be noted that the appendage of one or more non-enzymatic domains to GH domains is not uncommon in carbohydrate-active enzymes and is thought to guide the enzymatic domains towards their targets, as in the case of CBMs, and/or to provide sufficient spacing from the bacterial outer membrane for hydrolyzing glycans in the extracellular environment. However, the structural arrangement of the enzymatic and non-enzymatic domains of EndoS and EndoS2 is unique amongst known carbohydrate-active enzymes, as is the requirement for two of these connected domains to be absolutely necessary for activity.

The fourth mechanistic principle, sequential deglycosylation of the two Asn297-linked glycans on the IgG homodimer, is due to the biologically relevant state being a 1:1 complex of EndoS:Fc, as we observed in our cryoEM structure. When anchored by their protein-protein interfaces, the GH domains of EndoS and EndoS2 can reach only to the Asn297-linked glycan on the same Fc protomer that their β-sandwich domains engage. In order to hydrolyze the glycan on the other Fc protomer, the enzyme must unbind the substrate entirely, and rebind it through a protein-protein interaction on the opposite Fc protomer in order to properly reposition its GH domain. The release of the Fc product (mono- or de-glycosylated) might be favored by destabilization of the C´ strand and the C´E loop^58^ or by changes in the dynamic quaternary structure of IgG1-Fc after removal of the *N*-glycans^34,35^. Indeed, by intact mass spectrometry, we observed the accumulation of a mono-glycosylated antibody species, followed by its diminution. If the biologically relevant state were instead a 2:1 complex of EndoS:Fc, no such mono-glycosylated antibody species would exist, even transiently, as two EndoS enzymes would attack the antibody from each side simultaneously. Also, in our NMR experiments we observed that re-binding of the reaction product, a deglycosylated antibody, was highly disfavored. Despite this, a 2:1 complex formed between the enzyme and a di-glycosylated substrate can exist at high concentrations, as we observed as minor populations by both SAXS and cryoEM analysis (Supplementary Fig. 38).

With the molecular mechanism of IgG-specific deglycosylation established, opportunities for engineering EndoS-based enzymes to treat diseases of the adaptive immune system abound. It is now possible to rationally conceptualize engineered EndoS enzymes that selectively deglycosylate certain subsets of IgG antibodies, such as auto-antibodies, in order to defeat their effector functions and treat autoimmunity without wholesale IgG disablement that would render the host globally immunosuppressed and vulnerable to opportunistic infections.

## Methods

### Expression and purification of EndoS and EndoS2 wildtype and EndoS and EndoS2 mutants

EndoS and EndoS2 wild-type and EndoS and EndoS2 mutants were purified as previously described with the following modifications^26–28^. Single-point mutations were developed by PCR-based site-directed mutagenesis, and full sequences were confirmed by GeneWiz (Supplementary Table 3) (https://www.genewiz.com). EndoS wildtype and mutants were produced in *Escherichia coli* BL21(DE3) cells (Novagen, cat. No.: 69450) grown in LB medium supplemented with 100 μg mL^−1^ of ampicillin. Cultures were grown at 37 °C to an OD_600_ of 0.6–0.8, at which point the temperature was lowered to 22 °C for 1 h. Induction was triggered with 1 mM isopropyl β-D-1-thio-galactopyranoside (IPTG) at 22 °C overnight. Cells were harvested by centrifugation and lysed by sonication using 50 mM Tris-HCl pH 7.5, 500 mM NaCl, 10% glycerol. CPD fusion proteins were purified by Ni^2+^-immobilized metal-affinity chromatography followed by overnight treatment with 1 mM phytic acid at 4 °C and elution the next day with the lysis buffer. The proteins used for hydrolytic activity were further purified by size exclusion chromatography (Superdex 200 Increase 10/300 GL) in PBS as running buffer. EndoS_E235A_ was further purified by anion exchange chromatography using as binding buffer 50 mM Tris-HCl pH 8.5 and elution buffer 50 mM Tris-HCl pH 8.5, 500 mM NaCl. The protein was eluted using a gradient of 5% of elution buffer for 2 min. The fractions were pooled and aliquoted to flash freeze at -80 °C. For EM sample preparation, EndoS_E235A_ was thawed, and concentrated protein was purified by size exclusion chromatography (Superdex 200 Increase 10/300 GL) in PBS as running buffer.

### Purification of Fc-Rituximab

Rituximab (RITUXAN, Genentech) was kindly provided courtesy of the University of Maryland Greenebaum Comprehensive Cancer Center. A solution of 20 mg mL^−1^ of Rituximab was dialyzed against 20 mM sodium phosphate, 10 mM EDTA pH 7.0. A mixture of 0.5 mL of the Rituximab solution and 0.5 mL of the digestion buffer (20 mM sodium phosphate, 10 mM EDTA, 20 mM cysteine-HCl pH 7.0), was added to the 0.5 mL of the 50% immobilized papain slurry (Thermo Scientific) previously equilibrated with the digestion buffer. The reaction was incubated overnight in a shaker water bath at 37 °C at high speed. The reaction was stopped by adding 1.5 mL of 10 mM Tris-HCl pH 7.5 to the digest before centrifugation. After removing the supernatant, which contains the IgG fragments, the resin was washed with 1.5 mL of 10 mM Tris-HCl pH 7.5. The digest and wash fractions were combined and the Fc region was separated from the Fab fragments using an immobilized Protein A column (Thermo Scientific) and 100 mM glycine pH 3.0, as elution buffer. The eluted Fc was further purified by size exclusion chromatography using PBS to separate the Fc fragments from undigested IgG.

### Preparation and purification of the cross-linked EndoS_E235A_-Fc-Rituximab complex

The cross-linked EndoS_E235A_-Fc-Rituximab complex was prepared using a standard GraFix method^29,59^. Gradients for GraFix were prepared by mixing GraFix buffer 1 (PBS, pH 7.4, 5% v/v glycerol) and GraFix buffer 2 (PBS, pH 7.4, 20% v/v glycerol, 0.2 % glutaraldehyde) using a gradient mixer (Gradient Master ip, BioComp Instruments), following the parameters determined by the manufacturer for the glycerol content. A mixture of 100 μL of EndoS_E235A_ (12 μM) and 100 μL of Fc-Rituximab (60 μM) in PBS was incubated at room temperature for 10 min. The 200 μL solution mixture was loaded onto the top of the GraFix gradients and ultracentrifugation was carried out at 323,333 × *g* for 25 h at 4 °C (SW60 rotor, Beckmann). After ultracentrifugation, the gradients of each tube were fractionated using a Piston Gradient Fractionator^TM^ Base Unit (Sciences service) and a FC 203 Fraction Collector (Gilson) to pump the gradient out from bottom to top at room temperature. Each fraction of 250 μL was immediately quenched by 50 μL of 1 M Tris-HCl pH 8.0, to stop further crosslinking. The presence of the cross-linked EndoS_E235A_-Fc-Rituximab complex was confirmed by SDS-PAGE. Pooled fractions were flash frozen and stored at −80 °C.

### Negative-stained electron microscopy

Initial inspection of the GraFix EndoS_E235A_-Fc-Rituximab complex by negative stain was performed. Specifically, an aliquot from a single GraFix tube was thawed and samples of 50 μL were injected into an analytical size exclusion chromatography column (Shodex KW403-4F) using PBS pH 7.4, as running buffer. The single peak corresponding to the complex MW was collected in 50 μL fractions for subsequent standard negative stain grid preparation using continuous glow discharged carbon electron microscopy grids CF300-Ni (EMS, USA). Briefly, drops of 8 μL of diluted complex sample were placed on the grid, incubated for 90 s and then blotted to remove the excess with filter paper (Whatman®). The grids were washed by placing them onto a drop of PBS, pH 7.4 for 30 s and bottled dry. The complex was stained by transferring the grid to a drop of 1% w/v uranyl formate for 45 s, then dried with a filter paper and stored at room temperature. Samples were imaged at ×50,000 magnification and using an in-house JEOL 2200, 200 kV FEG equipped with an UltraScan 4000 CCD camera (Gatan, USA). Images were used to analyze particles by single particle analysis using RELION.

### Cryo-EM specimen preparation and screening

Cross-linked EndoS_E235L_-Fc-Rituximab complexes from four GraFix tubes were thawed, pooled, and concentrated using Pierce Protein Concentrators (PES 10 K MWCO 0.5 mL) to obtain a final volume of 60 μL. The sample was injected into an analytical size exclusion chromatography column (Shodex KW403-4F) using PBS pH 7.4, as running buffer. The complex peak was collected in collected in 50 μL fractions with to use directly for vitrification. Briefly, 4 μl of the fractions were applied onto copper Quantifoil R 1.2/1.3 grids previously glow discharged. Grids were plunge-frozen into liquid ethane using a Vitrobot MkII (FEI) at 10 °C and 80 HR% humidity in the chamber for each concentration condition by triplicate. One of the triplicates for each condition was used for cryoEM screening performed in-house using a JEOL 2200, 200 kV FEG equipped with an UltraScan 4000 CCD camera (Gatan, USA).

### CryoEM data collection

Cryo-EM data collection was carried out on a 300 kV Titan Krios microscope equipped with a K2 Summit direct electron-counting camera and a GIF Quantum energy filter (Gatan) in ESRF CM01 beamline at Grenoble, France^60^. Micrographs were recorded with EPU software (FEI) at a nominal magnification of ×130,000, with a pixel size of 1.052 Å. A total of 5546 movies of the cross-linked EndoS_E235A_-Fc-Rituximab grid sample were acquired for 9 s in counting mode at a flux of 7.3 electrons per pixel s^–1^, giving a total exposure of 59.37 electrons per Å^2^ and fractioned into 60 frames. A defocus range from −1.3 μm to −2.5 μm was used.

### CryoEM single-particle data processing

EndoS_E235_-Fc complex data set were processed using the general protocol described in the following lines. Data processing was initiated using the RELION 3.1.2^61^. The 60 movie frames of each movie were aligned using MotionCor2 (5 × 5 patches and Bfactor 150). The CTF of the aligned image was assessed in the non-dose weighted image using CtfFind 4.1 with parameters amplitude contrast 10% and FFT box size of 512 pixels. Image CTF was visually inspected, and with poor estimated maximum resolution or strong astigmatism from CtfFind calculations were discarded. We maintain 5354 micrographs from the original 5552 micrographs recorded. RELION reference-free automatic particle picking was performed considering a particle diameter between 95 Å and 110 Å, imposing a minimum inter-particle distance of 90 Å and a low picking threshold (0.05), leading to an initial number of particles (797,351). Particles were extracted using a square box of 180 pixels side. Particles were subjected to several rounds and extensive 2D classification selecting the best classes. An initial first 3D auto-refinement was performed using the map obtained from the negative stain images. This initial reconstruction was used to re-extract centered particles. Afterward, several steps of 3D classification were performed to discarding classes until models reached resolutions of 7 Å and visual inspection revealed a well-defined structure. The selected particles (485,424) were used to reconstruct a model using RELION 3D auto-refinement protocol, which led to a map resolving to 5.9 Å. At this point, the model was used to perform one round of RELION CTF refinement and particle polishing. The polished particles were used by RELION 3D auto-refinement to generate a map at 5.7 Å. Further 3D classification led to a set of selected particles (402,902) for a final RELION refinement reaching 5.3 Å resolution. Polished and CTF refined particles from RELION were imported into cryoSPARC v3.2.0^62^ and a final 2D classification of 200 classes was performed and the best-defined classes selected (Supplementary Fig. 3b) containing 372,643 particles. Final 3D reconstructions were performed in cryoSPARC using the homogeneous refinement and applying dynamic masking, using the RELION reconstructions low-pass at 30 Å as starting model. Finally, a final step of Non-Uniform refinement for the reconstruction^63^, on the corresponding symmetry, was performed applying a calculated global mask from the reconstruction. The reconstruction obtained from the non-uniform cryoSPARC protocol is reported (Supplementary Fig. 3g). Reported auto sharp-maps were obtained from the cryoSPARC non-uniform refinements (Supplementary Fig. 2c). Local resolution was measured in cryoSPARC LocRes program implementation^64^, using a threshold for local FSC = 0.5 for local resolution assessment (Supplementary Fig. 2c,d). For quality graphs as FSC plots, angular distribution and precision see (Supplementary Fig. 2e,f). For other statistics please see Supplementary Table 1.

### Model building and refinement

A model of the EndoS X-ray crystal structure (PDB code 6EN3) and the Fc region of IgG1-Fc (1H3X), was initially rigid-body fitted inside the cryoEM reconstruction using UCSF-Chimera^65^ followed by a rigid-body refinement of the individual chains using Phenix real space refinement^66,67^. Afterwards, a second rigid body refinement of the models using Phenix real space refinement^66,67^ was performed by defining 8 groups: EndoS individual domains and Fc individual protomers. However, map density was unequivocally observed for loop C´E loop and trisaccharide *N*-glycan attached to the N297. Remodeling based on the interpretation of this density due to the movement from loops in the interface between EndoS and IgG1-Fc, as well as the placement of carbohydrate in the density at the active site was performed iteratively and manually in Coot^68^ using the cryoSPARC sharp maps, following with rounds of Phenix real space refinement using local minimization, ADPs, and initial model as reference model to aid in keeping the geometry observed in the crystal structures. Side chains of the model residues were removed at this stage due to the resolution of the model as it is^69^. The quality of models was checked during refinements using Molprobity and validate-PDB server^70^. For model statistics and quality details please see Supplementary Table 1.

### EndoS_E235A_-Fc map principal component analysis and motion movies

A two-body multibody refinement of cryoEM images was performed as a continuation of the final RELION 3D auto-refinement^71^. The density corresponding to catalytically inactive EndoS was defined as body 1 and the Fc region as body 2. Masks were created for the top and bottom halves of the consensus reconstruction using the volume eraser tool in UCSF-Chimera^65^ and RELION3^61^ and used for a multibody refinement in RELION3. Principal component analysis was performed on the orientations of both bodies, and movies for the first three principal components (PCs) were written out as a series of volumes in MRC format^72^ describing the relative motion of the two bodies as described by these PCs (Supplementary Videos 1–3).

### Superposition of EndoS and Fc region crystal structures with the EndoS_E235A_-Fc cryoEM structure

In order to have insights on the possible location of the side chain residues that could be involved in the enzyme-substrate interactions, we locally compared/superimposed the high-resolution EndoS (PDB code 6EN3) and Fc region (PDB code 2DTS) crystal structures with our EndoS_E235A_-Fc cryoEM structure using the integrated sequence-structure analysis (MatchMaker) with UCSF Chimera^73^.

### SEC-SAXS experiments

A 50 μM solution of EndoS_E235A_ or EndoS2_E186L_ were incubated with 250 μM solution of Fc-Rituximab in 50 mM Tris-HCl pH 7.5, 100 mM NaCl, 2% *v/v* glycerol for 10 min at room temperature to form the EndoS(2)-Fc-Rituximab complexes. Small-Angle X-ray Scattering coupled with Size Exclusion Chromatography (SEC-SAXS) data for purified EndoS_E235A_, EndoS2_E186L_, Fc-Rituximab and the complex mixtures of EndoS_E235A_-Fc-Rituximab and EndoS2_E186L_-Fc-Rituximab in 50 mM Tris-HCl pH 7.5, 100 mM NaCl, 2% *v/v* glycerol were collected on the B21 beamline of the Diamond Light Source, UK. 50 µL of the protein samples or the complex mixtures were injected into a Shodex KW403-4F column and eluted at a flow rate of 150 μL min^−1^. Data were collected using a Pilatus2M detector (Dectris, CH) at a sample-detector distance of 3,914 mm and a wavelength of λ = 1 Å. The range of momentum transfer of 0.1 < s < 5 nm^−1^ was covered (*s* = 4πsinθ/λ, where θ is the scattering angle). Data were processed and merged using standard procedures by the program package ScÅter^74^ and PRIMUS^75^. The maximum dimensions (*D*_*max*_), the interatomic distance distribution functions (*P(r)*), and the radii of gyration (*Rg*) were computed using GNOM^76^. The molecular mass was determined using ScÅter^74^. The ab initio multiphase reconstruction of the SAXS data of the EndoS_E235A-_Fc-Rituximab and the EndoS2_E186L_-Rituximab complexes were generated using the MONSA algorithm from ATSAS^77^. The results and statistics are summarized in Supplementary Table 2.

### Cloning, expression and purification of EndoS mutants for alanine scan experiments

Primers to introduce single or multiple mutations or deletions into the EndoS-CPD construct were designed using the NebBaseChanger (https://nebasechanger.neb.com/) and PCR was carried out using the NEB Q5 High Fidelity polymerase and manufacturer’s instructions (Supplementary Table 3). For some of the alanine scan mutants, gene strings were ordered from Twist Bioscience and restriction cloned into the EndoS-CPD vector (Supplementary Table 3). All sequences were confirmed by Sanger sequencing at Genewiz (https://www.genewiz.com). For expression, the plasmids were transformed into *Escherichia coli* BL21 (DE3) cells and grown in 100 mL of LB medium supplemented by 100 µg mL^−1^ ampicillin at 37 °C. When the culture reached an OD of 0.6–0.8, the culture was induced with 0.5 mM IPTG and the culture was allowed to grow overnight at 22 °C. The cells were harvested by centrifugation at 3000 × *g* for 20 min and resuspended in 3 mL of PBS pH 7.4. The cells were lysed with BugBuster according to manufacturer’s instructions and the lysate clarified by spinning at 18,000 × *g* for 45 min. The clarified supernatant was incubated with NiNTA resin for 3 h before being washed with 10 column volumes (CV) of PBS pH 7.4. The EndoS was then eluted with 100 µM phytic acid in PBS pH 7.4. Protein purity was assessed by SDS-PAGE. All proteins were flash-frozen and stored at -80°C until ready for use.

### Cloning expression and purification of Fc mutants for alanine scan experiments

The sequence for the heavy chain of Rituximab was ordered from Thermo Fisher Scientific in the pcDNA3.4-TOPO vector for expression in HEK293 cells. The Fc plasmid was subcloned out of this plasmid via PCR. Primers to introduce single or multiple mutations or deletions into the Fc plasmid were designed using the NebBaseChanger (https://nebasechanger.neb.com/) and PCR was carried out using the NEB Q5 High Fidelity polymerase and manufacturer’s instructions (Supplementary Table 3). All sequences were confirmed by Sanger sequencing at Genewiz (https://www.genewiz.com). The Fc mutants were expressed in either HEK293T (ATTC, cat. No.: CRL-3216) or Expi293 (Thermo Fisher Scientific, cat. No.: A14527) cells. For HEK293T cells, the plasmids were transfected using polyethyleneimine as a transfection agent. After transfection, cells were cultured for 96 h in Free-style F17 medium supplemented with GlutaMAX and Geneticin (Thermo Fisher Scientific). For Expi293 cells, the plasmids were transfected as per manufacturers protocol (MAN0007814, Thermo Fisher Scientific) with the addition of Penicillin/Streptamycin mix 24 h after transfection. The cells were cultured for 96 h before harvesting. The Fc mutants were purified using Protein A chromatography with PBS pH 7.4 being used as the binding buffer and 100 mM sodium citrate buffer pH 3.0 as the elution buffer. The fractions were neutralized with 1 M Tris-HCl pH 9.3. SDS-PAGE was used to assess protein purity. The proteins were concentrated, flash-frozen and stored at -80°C until ready for use.

### Hydrolytic activity assays

For the EndoS-Fc alanine scan, LC-MS kinetic analysis was used to determine the reaction rate of deglycosylation by EndoS against Fc mutants and by EndoS mutants against Fc WT. 30 µL reactions were set up containing 5 µM of the Fc variant and 1–20 nM of the EndoS mutants in PBS pH 7.4. The reactions were analyzed by LC-MS using an Agilent 1290 Infinity II LC System equipped with a 50 mm PLRP-S column from Agilent with 1000 Å pore size. The LC system is attached to either an Agilent 6545XT quadrupole-time of flight (Q-TOF) or Agilent 6560 Ion Mobility (IM) Q-TOF mass spectrometer (Agilent, Santa Clara, CA). The reactions were setup and placed in the LC-MS and the reactions were sampled approximately every 15 min till they reached completion. All reactions were performed in triplicate. Relative amounts of the substrate and hydrolysis products were quantified after deconvolution of the raw data and identification of the corresponding peaks using BioConfirm (Agilent, Santa Clara, CA).

To obtain the rate of deglycosylation of Fc by the EndoS variants or Fc variants by EndoS, the relative proportions of the diglycosylated, monoglycosylated and deglycosylated products were imported into Kintek Global Kinetic Explorer^78^. This software fits the data to a model via the use of nonlinear regression analysis, where parameters are searched iteratively to determine a set of parameters which yields a minimum ${{{{\rm{\chi }}}}}^{2}$value. The following model was used for all the fitted reactions:

$$E+pGG \mathop{\rightleftharpoons }_{k-1}^{k1} EpGG \mathop{\rightleftharpoons }_{k-2}^{k2} EpG+G$$

$$E+pG \mathop{\rightleftharpoons }_{k-3}^{k3} EpG \mathop{\rightleftharpoons }_{k-4}^{k4} Ep+G$$

$$Ep \mathop{\rightleftharpoons }_{k-5}^{k5} E+p$$

Each of the independent experiments for a given set of mutations were imported into the Kintek Global Kinetic Explorer software into one experiment. All replicates for an experiment were fitted globally within the software. The rates were fitted in a stepwise manner starting from k1, and once the value which yielded a minimum ${\chi }^{2}$ was determined the value was locked. All values except for k2 and k4 were locked during the fitting process. Statistical significance was determined using a multiple comparisons test (Tukey method) in GraphPad (Graphpad Software, La Jolla, CA).

### Molecular dynamics (MD) simulations

The EndoS_E235A_-Fc complex obtained by cryoEM was used as starting coordinates for all MD simulations. The different mutants were generated starting from this structure and mutating the corresponding residues with PyMOL 2.5. The coordinates of the carbohydrate shown in Fig. 4 of the manuscript were generated using the GLYCAM WEB (https://glycam.org). This sugar was attached to the side chain of Asn297 of all simulated complexes. MD simulations were performed with AMBER 20 package^79^, implemented with ff14SB^80^, and GLYCAM 06j-1 force fields^81^. The LEaP module of AMBER20 was used to generate the topology and coordinate files for the MD simulations, which were carried out using the CUDA version of the PMEMD module of the AMBER simulation package. Each complex was immersed in a water box with a 10 Å buffer of TIP3P water molecules^82^ and the system was neutralized by adding explicit counter ions (Na^+^). A two-stage geometry optimization approach was performed with the PMEMD module. The first stage minimizes only the positions of solvent molecules and ions, using a 50 kcal mol^−1^ Å^−2^ harmonic potential, and the second stage is an unrestrained minimization of all the atoms in the simulation cell. In both stages, 2500 steps of steepest descent minimization were followed by 2500 steps of conjugate gradient minimization. The systems were then heated by incrementing the temperature from 0 to 300 K under a constant pressure of 1 atm and periodic boundary conditions for 2 ns. Harmonic restraints of 10 kcal mol^−1^ were applied to the solute, and the Andersen temperature coupling scheme^83^ was used to control and equalize the temperature. The time step was kept at 1 fs during the heating stages. The SHAKE algorithm was applied to constrain all bonds involving hydrogen atoms^84^. Long-range electrostatic effects were modeled using the particle-mesh-Ewald method^85^. A real-space cut-off of 8.0 Å was applied to electrostatic and Lennard-Jones interactions. Each system was equilibrated for 2 ns with a 2-fs time step at a constant volume and temperature of 300 K. Production trajectories were then run for additional 1 µs under the same simulation conditions. Three independent runs were performed for the wildtype, EndoS-FcE293A and EndoSW803A-Fc complexes.

### Protein expression and purification for NMR experiments

[*U*-^15^N,^2^H], ε-[^13^C,^1^H_3_]-Met, δ1-[^13^C^1^H_3_]- Ile, δ2-[^13^C,^1^H^3^]-Leu, γ2-[^13^C^1^H_3_]-Val-labeled (MILV) EndoS2, EndoS2_E186L_, EndoBT-3987 and EndoBT-3987_D312A/E314L_ were expressed following an adapted version from previously reported protocol^86^. Briefly, *E. coli* BL21(DE3) cells containing the gene of interest were grown in 20 mL of LB Lennox medium (Roth) until an optical density of 600 nm (OD_600_) > 1.5 was reached. Ampicillin (100 µg/mL) was used as selecting agent through the expression. Unless otherwise stated, bacteria were grown at 37 °C under shaking (220 rpm). Cells for inoculation of 10 mL M9^+^/D_2_O minimal medium with a starting OD_600_ of 0.1 were harvested by centrifugation, and excess of TB medium was removed. In all M9^+^/D_2_O minimal media, 3 g L^−1^ of ^15^N-ammonium chloride (Deutero) and 3 g L^−1^ of deuterated ^12^C-glucose (1,2,3,4,5,6,6-d_7_, Deutero) were used as the principal nitrogen and carbon sources, respectively. The next morning 2 mL of the starter culture were spin-down, supernatant was removed, and cells were transferred into 20 mL of freshly prepared M9^+^/D_2_O minimal medium. When an OD_600_ of 0.4 was reached, the culture volume was increased to 90 ml and cells were grown until an OD_600_ of 0.6–0.8 was reached. At this point, the temperature of the incubator was reduced to 16 °C and 10 mL of M9^+^/D_2_O minimal medium containing the desired labeled precursors and amino acids were added^86^. Amounts of isotopically labeled precursors for selective methyl labeling are given in Supplementary Table 4. Protein expression was induced with the addition of 1 mM or 0.5 mM isopropyl β-D-1-thio-galactopyranoside (IPTG) for EndoS2 or EndoBT constructs, respectively. Cells were harvested when the maximal cell density was reached and stored at -20 °C. Labeled protein was purified^28,42^ and stored at 4 °C.

### Sample preparation and NMR spectroscopy

All NMR experiments were acquired on a 600 MHz Avance III spectrometer equipped with TCI cryogenic probe at 298 K, unless otherwise stated. NMR spectra were processed with TopSpin 4.0.6 (Bruker) or with NMRPipe^87^ prior to analysis. ^1^H chemical shifts were referenced to the DSS-d_6_ peak, and ^13^C and ^15^N signals were referenced indirectly.

### Enzyme kinetics by NMR spectroscopy

Rituximab-Fc was concentrated using 10 kDa MWCO Amicon Ultra-4 filters (Merck) and the buffer was exchanged against NMR kinetics buffer, which contained 20 mM Tris-d_11_ (Eurisotop) pH 7.50, 50 mM NaCl, 0.1 mM 2,2-Dimethyl-2-silapentane-5-sulfonate-d_6_ (DSS-d_6_, Sigma-Aldrich) and 0.02% NaN_3_ in 8% D_2_O (Eurisotop, 99.96%). Samples were prepared per duplicate at 3, 5, 10, 20 and 40 µM Rituximab-Fc. All samples contained a final 160 µl volume in 3 mm NMR tubes.

For each kinetics experiment, the sample was introduced in the magnet, locked, matched, tuned, shimmed and the 90° pulse was calibrated. The temperature was allowed to equilibrate for 5 min and one perfect CPMG was acquired as reference (for details on the pulse sequence see Supplementary Fig. 18). The sample was removed from the magnet, 1 nM EndoS2_wt_ (2 µl) was added directly into the NMR tube, mixed 3x by inversion and the tube was immediately inserted into the magnet. After locking and shimming, a series of perfect CPMG spectra were recorded for a maximum of 24 h. From enzyme addition to the beginning of the first acquisition a max. of 3 min 20 s were measured, although <3 min were normally required. Perfect CPMGs were acquired with an echo time *τ* of 0.3 ms and a total *T*_*2*_ relaxation time of 39.6 ms, *T*_*1*_ relaxation delay (D1) of 1 s, 32 transients, 4, 32 or 64 dummy scans, an FID size of 32k and 12 ppm sweep width, with a total experimental time of 2 min. After completion of the reaction, a perfect CPMGs was acquired with a *T*_*1*_ relaxation delay (D1) of 20 s to correct for insufficient *T*_*1*_ relaxation.

Reaction time-courses were analyzed from the corrected integrals (*I*_*cor*_) obtained from the composite signal between 2.08-2.11 ppm, which corresponds to *N*-acetylglucosamine and *N*-acetylneuraminic acid moieties of cleaved *N*-glycans (Supplementary Fig. 19a). *I*_*cor*_ were calculated according to Eq. 1.

1 $${I}_{{cor}}=({I}_{n}-{I}_{{ref}})C$$

where *I*_*n*_ corresponds to the integral of the *n*^*th*^ experiment after the addition of enzyme, *I*_*ref*_ indicates the integral of the reference experiment (previous to enzyme addition) and *C* denotes a correction factor for insufficient *T*_*1*_ relaxation, calculated as the ratio between the integrals of the experiment acquired with D1 = 20 s and the last spectrum recorded with D1 = 1 s. *I*_*cor*_ were plotted against time to generate reaction time-courses (Supplementary Fig. 19b), which were then fitted to a single exponential decay Eq. 2. Initial enzyme cycling velocity ${v}_{0}$ was approximated as the first derivative of Eq. 2 evaluated at time = 0 (Eq. 3).

2 $${I}_{{cor}}(t)=1-{e}^{(-t\bullet a)}$$

3 $${v}_{0}={\left.\frac{d(1-{e}^{-t\bullet a})}{{dt}}\right|}_{t=0}=a$$

Initial enzyme cycling velocities ${v}_{0}$ were plotted against the initial concentration of complex glycan covalently attached to Fc or [S]_0_, which corresponds to two times the Fc concentration. Fitting to the Michaelis-Menten equation Eq. 4 afforded the substrate concentration-dependence of enzyme activation (*K*_M_) and the maximum velocity (*V*_*max*_). The maximum enzyme catalytic rate (*k*_*cat*_) was calculated as *V*_*max*_/[Enzyme]_0_. Fittings were performed using in-house *Matlab R2019b* script, and errors are given as one standard deviation.

4 $${v}_{0}=\frac{{V}_{\max }{[S]}_{0}}{{K}_{M}+{[S]}_{0}}$$

where

5 $${K}_{M}=\frac{{k}_{{off}}+{k}_{{cat}}}{{k}_{{on}}}$$

### Synthesis and purification of Rituximab-Fc aglycan and CT_n-1_ glycan

Rituximab-Fc aglycan was obtained by pooling the products of the enzymatic reactions of Rituximab-Fc with EndoS2 used for enzyme kinetics. Rituximab-Fc aglycan was separated from EndoS2 and free glycans by size exclusion chromatography (HiLoad 16/600 Superdex 200 pg, GE), using 20 mM Tris-HCl buffer pH 7.5, 50 mM NaCl as buffer. Fractions showing desired protein in UV (280 nm) were pooled, concentrated and the buffer was exchanged against NMR titration buffer as explained above. The final stock solution contained a concentration of 323 µM Rituximab-Fc aglycan.

The CT^27,88^ and HM^42,89,90^ glycan were synthesized following standard protocols. Glycan CT_n-1_ was obtained by digestion of CT with EndoS2 as explained in the previous chapter (enzyme kinetics by NMR spectroscopy). After reaction completion samples were lyophilized, dissolved in H_2_O and the reaction product CT_n-1_ was isolated by HPLC (Jasco Extrema) using a semipreparative VP 250/21 Nucleosil 100-7 C18 column (Macherey-Nagel, cat. No.: 715352) applying as solvents H_2_O and MeOH (both with 0.01% TFA) with a flow of 10 ml/min and the following gradients: 20 min 0% MeOH followed by 70 min 0% to 20% MeOH. Fractions of 10 ml were collected, and the desired sugar eluted in fractions 10 to 12, which were pulled together and lyophilized.

### Assignment of ^1^H,^13^C NMR signals from CT, CT_n-1_ and HM carbohydrates

The identity and purity of CT, CT_n-1_ and HM carbohydrates was confirmed by NMR prior titrations. For this end, all NMR signals from ^1^H,^13^C-HSQC spectra from samples were assigned. Samples contained 0.25 mM to 2.0 mM carbohydrate concentrations in 50 mM natrium phosphate buffer pH 7.30, 50 mM NaCl, 100 µM TSP-d4, 0.02% NaN_3_ in D_2_O in 3 mm NMR tubes at 160 µl total sample volume. Data was apodized with a QSINE window function, FIDs were zero-filled and forward linear-predicted in the indirect dimension prior Fourier-transformation (128 LP coefficients), affording a 4096 × 4096 data matrix. Spectra were manually phased and peak peaking was performed using CCPNMR Analysis 2.4.2 software suit^86^. Supplementary Fig. 20 shows a cartoon representation and the chemical structure of the assigned carbohydrates. Experimental details used for the assignment and ^1^H/^13^C chemical shifts are listed in Supplementary Table 5. Assignments are shown in Supplementary Fig. 21 and Supplementary Table 6. Assignments were in excellent agreement with previously reported frequencies^91,92^. All experiments for the assignment of carbohydrates were acquired at 310 K.

### Titration of selectively MILV methyl-labeled enzymes with carbohydrates, Rituximab-Fc, Rituximab-Fc aglycan and CaCl_2_

MILV methyl-labeled proteins were transferred into NMR titration buffer using 2 mL Zeba^TM^ Spin Desalting Columns (Thermo Fischer Scientific), which contained 20 mM Tris-d_11_ pH* 7.50, 50 mM NaCl, 0.1 mM DSS-d_6_ and 0.02% NaN_3_ in D_2_O. Protein samples were prepared at 45 – 128 µM protein concentration at 160 µl final volume in 3 mm NMR tubes. Protein concentrations were determined after buffer exchange by UV absorbance at 280 nm with ε = 104.17 M^−1^cm^−1^ for EndoS2 and EndoS2_E186L_, and with ε = 62.23 M^−1^cm^−1^ for EndoBT-3987 and EndoBT-3987_D312A/E314L_.

CT, CT_n-1_, HM and CaCl_2_ used for titrations were dissolved in D_2_O and lyophilized twice. Final stock solutions were prepared at high concentrations in NMR titration buffer, and the pH* was carefully readjusted to 7.50. Rituximab-Fc was buffer exchanged against NMR titration buffer using 2 mL Zeba^TM^ Spin Desalting Columns and concentrated to a final 380 µM concentration stock solution.

^1^H,^13^C HMQC spectra (methyl TROSY)^37^ were acquired with 107 ms acquisition time and a spectral window of 4 ppm in the direct dimension. In the indirect dimension, the spectral window was set to 22 or 24 ppm with 512 or 256 increments. The relaxation delay was set to 1.5 s and 4 to 16 transients were acquired. Data was apodized with a QSINE window function, FIDs were zero-filled and forward linear-predicted (64 LP coefficients) prior Fourier-transformation, affording a 2048 × 2048 data matrix with a spectral resolution of 1.17 Hz and 1.62 Hz in the direct and indirect dimensions, respectively.

Lineshape analysis of cross peaks of ^1^H,^13^C HMQC spectra of MILV-labeled samples with the program TITAN v1.6.−12-g9041^38^ was performed using *Matlab R2019b* for titrations shown in Table 1. Spectra were processed in NMRPipe^87^ prior to analysis. A representative example of the shell scripts used is compiled in Supplementary Table 7. We selected a two states binding model as the simplest model appropriately describing the experimental results. For each titration series the fitting algorithm was as follows: first, linewidths and chemical shift were fitted using the spectrum from the *apo* form. Next, linewidths and chemical shift of the fully bound state were fitted using the spectrum of the protein at highest ligand concentration. Linewidths, off-rate constant *k*_*off*_ and dissociation constant *K*_*D*_ were finally fitted from spectra of all titration points and using previously determined linewidths and chemical shifts as starting values. Parameter uncertainties were obtained by bootstrap error analysis with 100 iterations.

Titrations of CaCl_2_ showed fast-exchange in the NMR time-scale between the unbound and bound forms. Therefore, signal position were extracted with CCPNMR Analysis v2.4.2 software package^93^ and used to obtain Euclidean chemical shift perturbances (CSPs) measured as Euclidian distances Δν_Eucl_ according to Eq. 6:

6 $${\triangle v}_{{Eucl}}=\sqrt{{\triangle v}_{H}^{2}+{\triangle v}_{C}^{2}}$$

with ${\triangle v}_{H}$ and ${\triangle v}_{C}$ being the CSPs in the respective dimension in Hz. In a simple two states model, observed CSPs $\triangle {\nu }_{{obs}}$ at a given total ligand concentration *L*_*t*_ are linked to the dissociation constant *K*_D_ via the law of mass action^36^ (Eq. 7).

7 $$\triangle {\nu }_{{obs}}=\frac{\left({P}_{t}+{L}_{t}+{K}_{D}\right)-\sqrt{{\left({P}_{t}+{L}_{t}+{K}_{D}\right)}^{2}-4{P}_{t}{L}_{t}}}{2{P}_{t}}\triangle {\nu }_{\max }$$

where *P*_*t*_ is the total protein concentration, and $\triangle {\nu }_{\max }$ is the maximum CSP at ligand saturation for each signal. Global fittings were performed using in-house *Matlab R2019b* script. Errors were determined from a Monte Carlo approach with 100 iterations^94^, and are given as one standard deviation.

### ^1^H,^15^N HSQC-TROSY spectra and determination of rotational correlation times ${{{{\boldsymbol{\tau }}}}}_{{{{\boldsymbol{c}}}}}$

[*U*−^15^N,^2^H] MILV methyl-labeled proteins were buffer exchanged 2 mL Zeba^TM^ Spin Desalting Columns against the following buffer: 20 mM Tris-d_11_ pH* 7.50, 50 mM NaCl, 0.1 mM DSS-d_6_ and 0.02% NaN_3_ in 8% D_2_O. Two samples containing EndoS2_E186L_ and EndoBT_D312A/E314L_ were prepared at 71.5 µM or 74.4 µM protein concentrations, respectively. Samples were allowed to deuterium-hydrogen back exchange for 5 days at 6 °C, and then measured in 3 mm NMR tubes at a final 160 µl volume. ^1^H,^15^N HSQC-TROSY spectra were acquired with 128 ms acquisition time and a spectral window of 13.3 ppm in the direct dimension. In the indirect dimension, the spectral window was set to 45 ppm with 512 increments. The relaxation delay was set to 1.5 s and 16 (EndoBT-3987_D312A/E314L_) or 48 (EndoS2_E186L_) transients were acquired. Data was apodized with a QSINE window function, FIDs were zero-filled and forward linear-predicted (16 LP coefficients) prior Fourier-transformation, affording a 4096 × 1024 data matrix with a spectral resolution of 1.95 Hz and 2.67 Hz in the direct and indirect dimensions, respectively.

Rotational correlation times ${\tau }_{c}$ of MILV EndoS2_E186L_ and EndoBT_D312A/E314L_ proteins have been estimated from the samples prepared above using ^1^H,^15^N-TRACT experiments^95^. The relaxation delay was set to 2 s. Experiments were measured for 40 transients with 30 increasing delays of up to 1.5 s. Data were integrated from 8−10 ppm, normalized and fitted to an exponential decay model for determination of average ^15^N *R*_α_ and *R*_β._

### Reporting summary

Further information on research design is available in the Nature Portfolio Reporting Summary linked to this article.

## Supplementary information

Supplementary Information

Description of Additional Supplementary Files

Supplementary Movie 1

Supplementary Movie 2

Supplementary Movie 3

Reporting Summary

## Data Availability

The atomic coordinates of EndoS_E235A_-Fc have been deposited in the Protein Data Bank (PDB) under the accession code 8A64, and the cryoEM map are deposited in the Electron Microscopy Data Bank (EMDB) under the accession code EMD−15205. Previously published PDB structures used in this study are available under the accession codes: 6MDS, 4NUY, 2DTS, 1H3X and 6EN3. All other data are available from the corresponding authors upon request. Source data are provided with this paper.

## Source data

Source Data
