# Supplementary material for: Mechanism of antibody-specific deglycosylation and immune evasion by Streptococcal IgG-specific endoglycosidases
Source: Nat Commun. 2023 Mar 27;14:1705. doi: 10.1038/s41467-023-37215-3 (PMC10042849; doi:10.1038/s41467-023-37215-3)
Supplement: Supplementary file 1 — Supplementary Information [file 41467_2023_37215_MOESM1_ESM.pdf]

**SUPPLEMENTARY INFORMATION**

# Mechanism of antibody-specific deglycosylation and immune evasion by *Streptococcal* IgG-specific endoglycosidases

**Trastoy *et al.***

## Supplementary Table 1. CryoEM data collection, refinement and validation statistics

| | **$EndoS_{E235A}$-Fc** |
|---|---|
| **PDB ID** | **8A64** |
| **Data collection** | |
| Microscope | Titan Krios |
| Voltage (kV) | 300 |
| Detector | K2 Summit |
| Data collection software | EPU software (FEI) |
| Energy Filter | GIF Quantum |
| Magnification | 130,000 |
| Electron dose ($e^-/Å^2$) | 59.37 |
| Pixel size (Å) | 1.052 |
| Defocus range (μm) | -1.3 to -2.5 |
| Frames | 60 |
| Movie number | 5546 |
| **Data processing** | |
| Processing software | Relion 3.1.2/cryoSPARC v3.2.0 |
| Number of extracted particles | 339309 |
| EMDB code | 15205 |
| Map resolution (Å) at:<br>FSC=0.143 (unmasked/masked) | 5.2/4.54 |
| Map sharpening B factor (Å) | -330.0 |
| **Refinement** | |
| Software | PHENIX v1.18.2 |
| Models used (PDB code) | 6EN3 & 1H3X |
| Atoms (Non-H) | 6737 |
| Protein residues | 1354 |
| Ligands | 3 |
| Bonds (r.m.s.d.) | |
| Length (Å) | 0.008 |
| Angles (º) | 0.848 |
| Mean B-factor | |
| Protein | 196.14 |
| Ligand | 135.89 |
| MolProbity score | 2.20 |
| Clashscore | 20.41 |
| Rotamer outliers (%) | 0.0 |
| Ramachandran plot | |
| Favored (%) | 94.2 |
| Allowed (%) | 5.8 |
| CC (volume) | 0.76 |
| Mean CC for ligands | 0.59 |
| CC (mask) | 0.78 |

**Supplementary Table 2. SAXS data collection and refinement parameters.**

| Data collection parameters | $EndoS_{E235A}$-Fc | $EndoS2_{E186L}$-Fc | EndoS | EndoS2 | Fc |
|---|---|---|---|---|---|
| **Instrument** | B21 (DLS) | B21 (DLS) | B21 (DLS) | B21 (DLS) | B21 (DLS) |
| **Wavelength (Å)** | 0.99 | 0.99 | 0.99 | 0.99 | 0.99 |
| **S range ($Å^{-1}$)** | 0.002-0.420 | 0.002-0.420 | 0.002-0.420 | 0.002-0.420 | 0.002-0.420 |
| **Exposure time (s per frame)** | 0.56 | 0.56 | 0.56 | 0.56 | 0.56 |
| **Concentration range (μM)** | 55 | 55 | 55 | 55 | 55 |
| **Temperature (K)** | 298 | 298 | 298 | 298 | 298 |
| **Structure parameters** | | | | | |
| ***I*(0) (a.u.)[1] (from *P(r)*)** | 0.004 | 0.007 | 0.009 | 0.005 | 0.003 |
| ***Rg* (Å) (from *P(r)*)** | 53.7 | 52.2 | 43.0 | 38.7 | 26.8 |
| ***I*(0) (a.u.)[1] (from Guinier)** | 0.004 | 0.007 | 0.009 | 0.005 | 0.003 |
| ***Rg* (Å) (from Guinier)** | 53.6 | 52.2 | 42.9 | 38.7 | 26.8 |
| **$D_{max}$ ( Å)** | 182 | 176 | 148 | 125 | 80 |
| **Porod volume estimate ($Å^3$)** | 298375 | 324331 | 185228 | 168964 | 111242 |
| **Dry volume calculated from sequence ($Å^3$)[2]** | 187759 | 170507 | 127075 | 109823 | 60706 |
| **Molecular mass determination[3]** | | | | | |
| **Molecular weight (kDa)** | 211.3 | 234.1 | 111.9 | 87.0 | 52.6 |
| **Calculated MW from sequence (kDa)/ Discrepancy (%):** | | | | | |
| **Monomeric proteins** | - | - | 105.0/6.5 | 90.7/4.1 | 50.1/4.9 |
| **Enzyme:substrate (1:1)** | 155.1/36.7 | 140.8/40 | - | - | - |
| **Enzyme:substrate (2:1)** | 260.1/26.5 | 231.5/1.1 | - | - | - |
| **OLIGOMER:** | | | | | |
| **$\chi^2$ value** | 1.18 | 1.09 | - | - | - |
| **% of enzyme:substrate (1:1)** | 29 | 34 | - | - | - |
| **% of enzyme:substrate (2:1)** | 67 | 49 | - | - | - |
| **$\chi^2$ value MONSA** | 2.4 | 0.9 | 1.8 | 1.9 | 1.6 |
| **Software employed** | | | | | |
| **Primary data reduction** | *SCÅTTER* | *SCÅTTER* | *SCÅTTER* | *SCÅTTER* | *SCÅTTER* |
| **Data processing** | *PRIMUS/ SCÅTTER* | *PRIMUS/ SCÅTTER* | *PRIMUS/ SCÅTTER* | *PRIMUS/SC ÅTTER* | *PRIMUS/S CÅTTER* |

[1] arbitrary unit
[2] Dry volume determined using the server: http://biotools.nubic.northwestern.edu/proteincalc.html
[3] Molecular weight determination by SAXSMoW2

**Supplementary Table 3. Sequence of primers used to generate EndoS, EndoS2 and Fc mutants.**

| EndoS | Primers |
|---|---|
| H236A_F | GGATGTTGAAgcaGATAGTATTCCAAAAGTTGAC |
| H236A_R | ACATCTAAGCCATCAAGG |
| W314A_F | GAAAGGTGGTgcaGAGCCTGTTTC |
| W314A_R | TCTCCTTGTGAACCATAG |
| E315A_F | AGGTGGTTGGgcaCCTGTTTCTAATC |
| E315A_R | TTCTCTCCTTGTGAACCATAG |
| N319A_F | GCCTGTTTCTgcaCGACCTGAAAAAAC |
| N319A_R | TCCCAACCACCTTTCTCTC |
| K323A_F | TCGACCTGAAgcaACAATGGAAGAAC |
| K323A_R | TTAGAAACAGGCTCCCAAC |
| E354A_F | AAATGCTCAAgcaGGGAATCTTTG |
| E354A_R | TCCTCATAGAAAGAAAAACC |
| W803A_F | CTCTTTAGGAgcaGATTCTAAGCAAAGTATTATATTTAAATTG |
| W803A_R | ATATTATCAGTCTCACTGC |
| Y909A_F | AGGAGATAGAgcaAGTTCGCCAGTAGTC |
| Y909A_R | TTAGTATCAAAGACAACTCG |
| **Strings for EndoS alanine scan** | **Fragment** |
| ΔLoop411-428[1] | GAAGTGCCATTCCGCCTGACCTGCGCGCGGAGCTCCTTACATCAACCTTC<br>TCCTCGTTCAAGTGTACGGCTCGCAGGGCGAAAAGGGCGGGTGGAACC<br>GGTGTCAAACCGCCCGGAGAAGACCATGGAAGAGCGTTGGCAGGGCTAC<br>TCGAAATACATCCGCCCGGAGCAGTATATGATCGGCTTCAGCTTTTACGA<br>AGAGAACGCGCAGGAAGGCAACCTGTGGTACGACATCAACTCACGTAAA<br>GATGAAGATAAGGCCAACGGCATCAATACCGATATTACCGGCACTCGCG<br>CGGAGCGCTACGCCCGCTGGCAGCCGAAAACTGGCGGCGTGAAAGGTGG<br>AATCTTTAGCTATGCCATCGATCGCGACGGAGTTGCGATTTTCCATAGCG<br>ACTACTCAGTGTCGAAAGCCCTGAAAACTGTGATGCTGAAGGACAAATC<br>ATACGACCTCATCGACGAAAAGGACTTTCCTGACAAAGCCCTTCGCGAGG<br>CCGTTATGGCTCAAGTGGGGACGCGCAAAGGCGACCTGGAGCGCTTTAA<br>CGGTACGCTGCGCCTGGACAACCCGGCTATCCAGAGCCTTGAGGGACTGA<br>ACAAGTTCAAGAAGCTGGCGCAGCTGGATCTGATCGGTCTTAGCCGTATC<br>ACCAAACTGGATCGCTCAGTCCTGCCTGCCAACATGAAACCGGGGAAGG<br>ACACGCTCGAGACTGTGCTGGAGACATACAAGAAAGACAATAAAGAGGA<br>GCCGGCGACCATTCCGCCGGTGAGTCTGAAAGTCTCGGGGTTGACAGGAC<br>TCAAGGAGCTGGACCTTTCTGGCTTCGATCGCGAGACACTGGCAGGGCTC<br>GACGCGGCGACATTAACTTCATTGGAGAAGGTGGACATCAGCGGGAATA<br>AGCTGGACCTTGCGCCGGGTACGGAGAACCGCCAGATCTTCGACACGAT<br>GCTTAGCACCATTTCCAACCACGTCGGCTCGAACGAGCAGACGGTCAAGT<br>TCGATAAACAGAAGCCTACGGGTCACTATCCCGACACATACGGAAAGAC<br>CTCATTACGTTTGCCGGTTGCCAACGAGAAGGTAGACCTGCAGTCACAAT<br>TGCTGTTCGGCACCGTAACCAACCAGGGTACTCTGATTAACAGTGAGGCC<br>GATTACAAAGCATATCAGAACCACAAGATCGCCGGTCGCAGTTTCGTGGA<br>CTCGAATTACCACTATAACAATTTCAAGGTCAGCTACGAAAATTACACAG<br>TGAAGGTTACAGACAGCACTCTGGGGACTACCACGGATAAGACGCTAGC<br>AGCGCGCAGGCTAGGTGGAGGCTCAGTG |
| K70A/Q73A/E74A/Q76A/K77A[2] | GAAGTGCCATTCCGCCTGACCTGCGCGCCATATGGAAGAAAAGACAGTG<br>CAAGTTCAAAAGGGGCTCCCGTCCATTGACTCATTACACTACCTCTCCGA<br>AAACAGTAAGAAGGAGTTCAAGGAAGAGTTGAGCGCCGCCGGCGCCGCT |

**Supplementary Table 3** (continued).

| | |
|---|---|
| | TCGGCGGCCGTAAAGGAAATTCTTGCGAAGGCCCAACAAGCCGACAAGC<br>AGGCGCAGGAGTTGGCAAAGATGAAGATCCCCGAAAAGATCCCCATGAA<br>GCCACTGCACGGCCCACTGTATGGCGGCTATTTCCGTACCTGGCACGATA<br>AGACCAGTGACCCGACGGAGAAGGATAAGGTCAATAGTATGGGTGAATT<br>ACCGAAAGAGGTTGACCTGGCGTTCATCTTTCATGACTGGACTAAGGACT<br>ACTCGTTATTCTGGAAGGAGCTGGCAACTAAGCACGTTCCGAAACTGAAT<br>AAACAGGGTACGCGCGTTATCCGCACGATCCCGTGGCGCTTTCTGGCAGG<br>TGGCGACAATTCGGGGATCGCGGAAGACACAAGCAAGTATCCCAACACC<br>CCGGAAGGCAACAAAGCTTTAGCGCGCAGGCTAGGTGGAGGCTCAGTG |
| W314A/E315A/N319A/K323A[1] | GAAGTGCCATTCCGCCTGACCTGCGCGCGGAGCTCCTTACATCAACCTTC<br>TCCTCGTTCAAGTGTACGGGTCTCAGGGTGAAAAGGGCGGGGCCGCCCCG<br>GTGAGTGCACGCCCTGAGGCCACTATGGAAGAGCGTTGGCAGGGCTACT<br>CTAAATACATCCGCCCCGAGCAGTATATGATCGGCTTCAGTTTCTACGAA<br>GAGAACGCCCAAGAGGGTAACCTGTGGTACGACATCAACTCGCGTAAAG<br>ATGAAGATAAGGCGAACGGCATCAATACCGATATCACCGGTACCCGCGC<br>AGAGCGCTACGCGCGCTGGCAGCCCAAAACGGGCGGCGTCAAAGGCGGA<br>ATTTTCAGTTATGCCATCGATCGCGACGGAGTTGCGCACCAGCCCAAGAA<br>GTACGCAAAGCAAAAGGAATTCAAAGATGCGACCGACAATATTTTCCAT<br>AGCGACTACAGCGTGTCTAAAGCTCTGAAAACCGTAATGCTTAAGGACA<br>AAAGCTACGACTTAATCGACGAAAAGGACTTTCCTGACAAAGCCCTGCGC<br>GAGGCTGTCATGGCACAAGTAGGCACGCGCAAGGGCGACCTGGAGCGCT<br>TTAACGGTACCTTGCGCCTGGACAACCCCGCCATCCAGTCACTCGAGGGC<br>CTGAACAAGTTCAAGAAGCTGGCACAGCTGGATCTTATCGGTTTGTCACG<br>TATCACCAAATTGGATCGCAGTGTACTTCCAGCGAACATGAAACCCGGTA<br>AGGACACGCTTGAGACTGTGCTGGAGACGTACAAGAAAGACAACAAAGA<br>GGAGCCAGCGACCATTCCGCCGGTGTCCCTGAAAGTGAGCGGCCTCACG<br>GGCCTTAAAGAGTTGGACCTTAGCGGCTTCGATCGCGAGACTCTCGCAGG<br>CTTAGACGCGGCAACCCTGACCAGTTTGGAGAAGGTAGACATCAGCGGA<br>AATAAGCTGGACCTGGCCCCGGGTACTGAGAACCGTCAGATCTTCGACAC<br>GATGTTGTCTACCATTTCGAACCACGTGGGCAGTAACGAGCAGACGGTAA<br>AGTTCGATAAACAGAAGCCTACCGGTCACTATCCGGACACGTACGGAAA<br>GACATCGCTTCGTTTGCCGGTCGCCAACGAGAAGGTCGACCTGCAGTCCC<br>AACTGCTGTTCGGTACGGTAACTAACCAGGGTACATTGATTAACAGCGAG<br>GCGGATTACAAAGCCTATCAGAACCACAAGATCGCGGGCCGCTCTTTCGT<br>GGACAGCAATTACCACTATAACAATTTCAAGGTGAGTTACGAAAATTACA<br>CTGTCAAGGTTACCGACTCGACTCTGGGCACTACGACCGATAAGACGCTA<br>GCAGCGCGCAGGCTAGGTGGAGGCTCAGTG |
| E354A/K414A/K418A[1] | GAAGTGCCATTCCGCCTGACCTGCGCGCGGAGCTCCTTACATCAACTTGC<br>TGTTGGTTCAAGTATACGGAAGCCAGGGTGAAAAGGGCGGCTGGGAACC<br>CGTGAGCAACCGTCCAGAGAAGACGATGGAAGAGCGCTGGCAGGGCTAC<br>TCGAAATACATCCGCCCGGAGCAGTATATGATCGGGTTCTCATTTTACGA<br>AGAGAACGCGCAGGCGGGTAACCTGTGGTACGACATCAACTCGCGTAAA<br>GATGAAGATAAGGCGAACGGCATCAATACAGATATTACGGGCACACGCG<br>CAGAGCGTTACGCGCGTTGGCAGCCCAAAACCGGCGGAGTGAAAGGCGG<br>GATCTTTAGTTATGCCATCGATCGCGACGGAGTCGCGCACCAGCCGGCGA<br>AGTACGCCGCGCAAAAGGAATTCAAAGATGCCACCGACAATATCTTTCAT<br>AGTGACTACTCAGTTTCAAAAGCGCTGAAAACTGTAATGCTTAAGGACAA<br>AAGTTACGACTTGATCGACGAAAAGGACTTTCCGGACAAAGCCCTGCGC<br>GAGGCCGTTATGGCACAAGTGGGTACGCGCAAGGGCGACTTAGAGCGCT<br>TTAACGGTACTCTGCGCCTGGACAACCCCGCAATCCAGTCTCTTGAGGGA<br>CTGAACAAGTTCAAGAAGCTGGCCCAGCTGGATCTCATCGGTTTGTCCCG<br>TATCACGAAACTGGATCGCTCGGTGCTTCCGGCCAACATGAAACCGGGTA<br>AGGACACTCTCGAGACCGTCTTAGAGACATACAAGAAAGACAATAAGGA<br>AGAGCCAGCAACAATTCCGCCGGTGAGTCTGAAAGTAAGCGGCCTGACC<br>GGCTTAAAGGAGTTGGACCTCTCGGGGTTCGATCGTGAGACACTGGCGGG<br>CCTGGACGCGGCCACGCTGACCAGCTTGGAGAAGGTAGACATCAGCGGT<br>AATAAGTTGGACTTAGCCCCGGGGACCGAGAACCGTCAGATCTTCGACAC<br>AATGCTCTCGACCATTTCGAACCACGTGGGCTCGAACGAGCAGACGGTAA<br>AGTTCGATAAACAGAAGCCGACCGGTCACTATCCGGACACTTACGGCAA<br>GACCAGCTTACGTCTGCCCGTTGCGAACGAGAAGGTAGACTTACAGTCTC |

**Supplementary Table 3** (continued).

| | |
|---|---|
| | AACTGCTGTTCGGTACGGTTACGAACCAGGGTACGCTTATTAACTCGGAA<br>GCGGATTACAAAGCCTATCAGAACCACAAGATCGCCGGTCGCAGTTTCGT<br>GGACAGCAATTACCACTATAACAATTTCAAGGTCTCGTACGAAAATTACA<br>CGGTAAAGGTCACGGACAGCACGTTAGGGACGACCACGGATAAGACGCT<br>AGCAGCGCGCAGGCTAGGTGGAGGCTCAGTG |
| Y416A/Q419A/K420A/E421A/F422A/K423A[1] | GAAGTGCCATTCCGCCTGACCTGCGCGCGGAGCTCCTTACATCAACCTGC<br>TGTTAGTCCAAGTGTACGGCAGCCAGGGCGAAAAGGGCGGCTGGGAACC<br>GGTATCCAACCGTCCAGAGAAGACCATGGAAGAGCGTTGGCAGGGCTAC<br>AGTAAATACATCCGCCCGGAGCAGTATATGATCGGGTTCTCGTTTTACGA<br>AGAGAACGCGCAGGAAGGCAACTTGTGGTACGACATCAACAGCCGTAAA<br>GATGAAGATAAGGCGAACGGCATCAATACCGATATTACGGGTACTCGCG<br>CAGAGCGCTACGCGCGCTGGCAGCCGAAAACCGGCGGCGTTAAAGGCGG<br>AATCTTTAGTTATGCCATCGATCGCGACGGCGTCGCCCACCAGCCTAAGA<br>AGGCCGCAAAGGCTGCCGCCGCCGCCGATGCTACCGACAATATCTTTCAT<br>TCCGACTACTCCGTTTCTAAAGCGCTCAAAACGGTGATGCTGAAGGACAA<br>AAGCTACGACTTGATCGACGAAAAGGACTTTCCGGACAAAGCCTTACGTG<br>AGGCGGTAATGGCCCAAGTCGGCACTCGTAAGGGCGACCTCGAGCGCTTT<br>AACGGTACTCTGCGCCTGGACAACCCTGCCATCCAGTCATTGGAAGGCTT<br>AAACAAGTTCAAGAAGTTGGCCCAGTTGGATCTTATCGGGTTGTCCCGTA<br>TCACCAAACTGGATCGCTCGGTACTGCCAGCAAACATGAAACCCGGTAA<br>GGACACTCTGGAGACGGTGCTGGAGACTTACAAGAAAGACAATAAAGAG<br>GAGCCGGCCACCATTCCGCCGGTTAGTCTTAAAGTGTCAGGACTGACCGG<br>ACTCAAGGAGCTGGACCTGAGCGGGTTCGATCGCGAGACGCTTGCCGGC<br>CTGGACGCAGCGACCCTGACATCGCTCGAAAAGGTGGACATCAGCGGTA<br>ATAAGCTCGATCTGGCGCCGGGGACCGAGAACCGCCAGATCTTCGACAC<br>GATGTTATCGACCATTTCTAACCACGTGGGCTCCAACGAGCAGACCGTAA<br>AGTTCGATAAACAGAAGCCGACCGGCCACTATCCTGACACTTACGGTAAG<br>ACGAGCCTTCGTCTCCCGGTAGCCAACGAGAAGGTGGACCTGCAGTCTCA<br>ATTACTGTTCGGCACGGTAACGAACCAGGGCACGTTAATTAACAGCGAG<br>GCGGATTACAAAGCGTATCAGAACCACAAGATCGCAGGCCGCTCATTCGT<br>GGACTCCAATTACCACTATAACAATTTCAAGGTATCCTACGAAAATTACA<br>CGGTAAAGGTCACAGACAGTACCTTAGGCACAACCACGGATAAGACGCT<br>AGCAGCGCGCAGGCTAGGTGGAGGCTCAGTG |
| Y541A/K543A/D544A/N545A/K546A[1] | GAAGTGCCATTCCGCCTGACCTGCGCGCGGAGCTCCTTACATCAACCTGC<br>TTCTTGTTCAAGTGTACGGCAGCCAGGGCGAAAAGGGCGGGTGGGAACC<br>AGTAAGTAACCGCCCGGAGAAGACGATGGAAGAGCGTTGGCAGGGCTAC<br>TCCAAATACATCCGCCCGGAGCAGTATATGATCGGCTTCTCCTTTTACGA<br>AGAGAACGCCCAAGAGGGTAACCTCTGGTACGACATCAACAGCCGTAAA<br>GATGAAGATAAGGCCAACGGGATCAATACGGATATTACCGGCACCCGCG<br>CAGAGCGTTACGCGCGCTGGCAGCCGAAAACTGGCGGTGTCAAAGGCGG<br>AATCTTTAGCTATGCGATCGATCGCGACGGCGTGGCGCACCAGCCAAAGA<br>AGTACGCCAAGCAAAAGGAATTCAAAGATGCCACCGACAATATCTTTCAT<br>AGCGACTACTCCGTGAGTAAAGCTCTGAAAACCGTGATGCTGAAGGACA<br>AAAGTTACGACCTTATCGACGAAAAGGACTTTCCGGACAAAGCCTTACGC<br>GAGGCCGTTATGGCCCAAGTAGGTACGCGTAAGGGCGACCTGGAGCGCT<br>TTAACGGGACCCTGCGCCTTGACAACCCGGCCATCCAGTCGCTTGAGGGG<br>CTGAACAAGTTCAAGAAGCTGGCACAGCTTGATCTGATCGGTCTTAGTCG<br>TATCACCAAACTGGATCGCTCCGTGCTGCCGGCAAACATGAAACCGGGTA<br>AGGACACTCTGGAGACTGTGTTAGAGACGGCCAAGGCCGCCGCAGCGGA<br>AGAGCCCGCCACGATTCCGCCGGTGAGCCTGAAAGTGAGTGGCTTAACA<br>GGCTTGAAGGAGCTGGACTTAAGCGGCTTCGATCGCGAGACGCTGGCGG<br>GGTTGGACGCGGCCACCCTGACTAGTCTTGAGAAGGTCGACATCAGCGGT<br>AATAAGCTGGACCTGGCGCCGGGCACGGAGAACCGTCAGATCTTCGACA<br>CGATGCTGAGTACCATTTCGAACCACGTGGGCTCTAACGAGCAGACCGTC<br>AAGTTCGATAAACAGAAGCCGACCGGACACTATCCGGACACTTACGGTA<br>AGACGTCACTCCGTCTGCCTGTCGCGAACGAGAAGGTAGACCTGCAGTCA<br>CAACTGTTATTCGGAACGGTTACCAACCAGGGCACACTGATTAACTCTGA<br>GGCCGATTACAAAGCGTATCAGAACCACAAGATCGCAGGGCGCTCATTC<br>GTGGACTCTAATTACCACTATAACAATTTCAAGGTCTCGTACGAAAATTA |

**Supplementary Table 3** (continued).

| | |
|---|---|
| | CACGGTGAAGGTGACCGACAGCACCCTGGGCACTACCACCGATAAGACG CTAGCAGCGCGCAGGCTAGGTGGAGGCTCAGTG |
| ΔLoop 314-323[1] | GAAGTGCCATTCCGCCTGACCTGCGCGCGGAGCTCCTTACATCAACCTGC TGCTCGTTCAAGTGTACGGCTCTCAGGGCGAAAAGGGCGGCACCATGGA AGAGCGTTGGCAGGGATACTCCAAATACATCCGCCCGGAGCAGTATATG ATCGGCTTCTCATTTTACGAAGAGAACGCCCAGGAAGGTAACCTGTGGTA CGACATCAACAGCCGTAAAGATGAAGATAAGGCGAACGGCATCAATACC GATATTACAGGGACTCGCGCGGAGCGTTACGCGCGTTGGCAGCCTAAAA CTGGTGGAGTCAAAGGCGGAATCTTTAGCTATGCAATCGATCGCGACGGC GTCGCGCACCAGCCGAAGAAGTACGCGAAGCAAAAGGAATTCAAAGATG CCACCGACAATATCTTTCATTCGGACTACTCAGTGAGCAAAGCGCTGAAA ACGGTGATGCTGAAGGACAAATCATACGACTTGATCGACGAAAAGGACT TTCCGGACAAAGCGTTACGCGAGGCCGTAATGGCACAAGTAGGTACACG CAAAGGGGACTTAGAGCGCTTTAACGGTACCCTGCGCTTAGACAACCCGG CCATCCAGAGCTTGGAAGGCCTCAACAAGTTCAAGAAGCTCGCCCAGCTC GATCTGATCGGACTGTCGCGTATCACCAAACTGGATCGCTCGGTGCTGCC TGCAAACATGAAACCGGGTAAGGACACTCTTGAGACCGTGCTGGAGACA TACAAGAAAGACAATAAGGAAGAGCCCGCAACCATTCCGCCGGTTAGCC TCAAAGTGAGTGGACTGACGGGGTTGAAGGAGCTGGACCTGAGTGGATT CGATCGCGAGACGCTGGCCGGCCTGGACGCGGCGACTCTTACTTCACTGG AGAAGGTGGACATCTCGGGGAATAAGCTGGACCTCGCCCCGGGTACCGA GAACCGCCAGATCTTCGACACCATGTTGAGCACCATTTCTAACCACGTGG GTTCGAACGAGCAGACCGTAAAGTTCGATAAACAGAAGCCGACCGGACA CTATCCGGACACGTACGGTAAGACCAGCCTTCGTCTGCCGGTAGCCAACG AGAAGGTCGACCTGCAGTCTCAACTGCTTTTCGGCACCGTCACGAACCAG GGCACGCTCATTAACTCTGAGGCCGATTACAAAGCGTATCAGAACCACAA GATCGCCGGTCGCTCGTTCGTAGACTCCAATTACCACTATAACAATTTCA AGGTCAGCTACGAAAATTACACGGTAAAGGTCACGGACAGCACTCTTGG GACTACCACGGATAAGACGCTAGCAGCGCGCAGGCTAGGTGGAGGCTCA GTG |
| ΔLoop 828-836[3] | GAAGTGCCATTCCGCCTGACCTGCGCGCACGCTAGCAACCGACAAAGAG GAAACTTACAAAGTCGATTTCTTCAGTCCTGCGGACAAAACCAAGGCGGT GCACACCGCAAAGGTAATCGTAGGCGATGAGAAGACAATGATGGTGAAC CTCGCGGAAGGGGCCACCGTGATTGGCGGCAGCGCCGATCCAGTCAACG CCCGCAAAGTGTTCGACGGCCAGCTTGGGTCCGAAACCGACAACATTAGC CTTGGCTGGGACAGCAAACAGTCCATCATTTTCAAGCTGAAAGAGGACG GTCTGATCAAACACTGGCGCTTCTTTAACGACAAGCCGATCCAAGAGGCG TCACTGCAGATCTTCAACATTAAGGACTACAACCTCGACAACCTGCTTGA GAACCCGAACAAGTTCGACGACGAGAAGTACTGGATCACCGTGGACACA TATTCCGCGCAAGGGGAACGCGCGACCGCCTTTAGCAACACCTTGAACAA CATCACCTCAAAGTACTGGCGCGTGGTGTTCGACACCAAGGGTGACCGTT ACTCGTCACCGGTTGTGCCCGAGCTGCAGATCCTTGGGTACCCCCTGCCG AATGCGGATACAATTATGAAGACCGTTACCACAGCCAAGGAACTGTCGC AGCAGAAGGACAAATTCTCGCAGAAAATGTTAGACGAACTGAAGATCAA GGAAATGGCGCTGGAGACCAGCCTGAATAGCAAAATCTTCGACGTTACG GCGATCAACGCCAACGCGGGTGTGCTGAAGGACTGCATCGAAAAGCGTC AACTCCTGAAGAAGGGATCCGGCGCGCGCAGGCTAGGTGGAGGCTCAGT G |
| **Fc region** | **Primers** |
| R292A_F | GACCAAGCCTgctGAGGAACAGTACAAC |
| R292A_R | TTGGCGTTGTGCACTTCC |
| E293A_F | CAAGCCTAGAgctGAACAGTACAACAGC |
| E293A_R | GTCTTGGCGTTGTGCACT |
| E294A_F | GCCTAGAGAGgctCAGTACAACAGC |
| E294A_R | TTGGTCTTGGCGTTGTGC |
| Q295A_F | TAGAGAGGAAgctTACAACAGCACCTACAGAGTGGTG |
| Q295A_R | GGCTTGGTCTTGGCGTTG |

**Supplementary Table 3** (continued).

| | |
|---|---|
| Y296A_F | AGAGGAACAGgctAACAGCACCTACAGAG |
| Y296A_R | CTAGGCTTGGTCTTGGCG |
| S298A_F | ACAGTACAACgctACCTACAGAGTGGTG |
| S298A_R | TCCTCTCTAGGCTTGGTC |
| S298A/Y296A/E294A/R292A_F | ggctaacgctACCTACAGAGTGGTGTCC |
| S298A/Y296A/E294A/R292A_R | tgagcctcagcAGGCTTGGTCTTGGCGTT |
| Y296F_F | AGAGGAACAGttcAACAGCACCTAC |
| Y296F_R | CTAGGCTTGGTCTTGGCG |
| Y300F_F | CAACAGCACCttcAGAGTGGTGT |
| Y300F_R | TACTGTTCCTCTCTAGGC |
| Y296_Y300F_F | caccttcAGAGTGGTGTCCGTGCTG |
| Y296_Y300F_R | ctgttgaaCTGTTCCTCTCTAGGCTTGG |
| I253A_F | CACCCTGATGgctAGCAGAACCC |
| I253A_R | TCCTTAGGCTTTGGAGGA |
| H310A_F | GACCGTGCTGgctCAGGATTGGCTG |
| H310A_R | AGCACGGACACCACTCTG |
| H310A/L314A_F | ttgggctAACGGCAAAGAGTACAAGTGC |
| H310A/L314A_R | tcctgagcCAGCACGGTCAGCACGGA |
| Q311A/N315A_F | gctggctGGCAAAGAGTACAAGTGCAAGG |
| Q311A/N315A_R | caatcagcGTGCAGCACGGTCAGCAC |
| E430A/H435A_F | cacaacgctTACACCCAGAAAAGCCTGTC |
| E430A/H435A _R | cagggcagcGTGCATCACGCTGCAGCT |
| H268A/E269A/D270A/E272A_F | tcccgctGTGAAGTTCAATTGGTACGTG |
| H268A/E269A/D270A/E272A_R | gcagcagcGGACACATCCACCACCAC |
| P329A_F | CAAGGCCCTGgccGCTCCTATCG |
| P329A_R | TTGGACACCTTGCACTTGTAC |
| A330G_F | GGCCCTGCCTggcCCTATCGAGA |
| A330G_R | TTGTTGGACACCTTGCAC |
| A330W_F | GGCCCTGCCTtggCCTATCGAGA |
| A330W_R | TTGTTGGACACCTTGCAC |
| P329A/A330G_F | CAAGGCCCTGgccggcCCTATCGAGAAAAC |
| P329A/A330G_R | TTGGACACCTTGCACTTG |
| P329A/A330W_F | CAAGGCCCTGgcctggCCTATCGAGAAAAC |
| P329A/A330W_R | TTGGACACCTTGCACTTG |
| Loop325-331(NKALPAP-->GGGSGGG)_F | cggagggggtATCGAGAAAACCATCAGC |
| Loop325-331(NKALPAP-->GGGSGGG)_R | gaaccgccaccGGACACCTTGCACTTGTAC |
| Loop325-331(NKALPAP-->GGGGPAG)_F | ccctgctgggATCGAGAAAACCATCAGC |

**Supplementary Table 3** (continued).

| | |
|---|---|
| Loop325-331(NKALPAP-->GGGGPAG)_R | ccaccgccaccGGACACCTTGCACTTGTAC |

[1] Restriction site for cloning: SacI and NheI
[2] Restriction site for cloning: NdeI and HindIII
[3] Restriction site for cloning: NheI and BamH

**Supplementary Table 4. Precursors and quantities used for the preparation of [*U*-$^{15}N,^{2}H$] MIL$^{proS}$V$^{proS}$ [$^{13}C,^{1}H_3$]-methyl labeled endoglycosidases.** A systematic review on precursors required for optimal methyl group isotope labelling in *E. coli* can be found somewhere else[1].

| Labeled amino acid | Precursor | Quantity (mg/L) |
|---|---|---|
| Met-ε | L-Methionine (6-$^{13}C$)[a] | 250 |
| Ile-δ1 | 2-ketobutyric acid (4-$^{13}C$;3,3-$D_2$) | 60 |
| Leu-δ2, Val-γ2 pro-(*S*) | 2-hydroxy-2-($^{13}C$)methyl-3-oxobutanoate (4-$D_3$)[b] | 120 |

Obtained from [a]Aldrich, [b]NMR-Bio. 2-ketobutyric acid (4-$^{13}C$;3,3-$D_2$) was synthesized in-house.

**Supplementary Table 5. List of NMR experiments and experimental details used for the assignment of CT, CTn-1 and HM *N*-glycans. Experiments were acquired at 310 K.**

| Experiment | Pulse program from Bruker library | NS | TD | SW (ppm) | Carrier F2/F1 (ppm) | Observations |
|---|---|---|---|---|---|---|
| $^{1}H$ | zgesgp | 64 | 32768 | 11.65 | 4.7 | Water suppression with Squa100.1000 shaped pulse, 8 ms length to minimise suppression of anomeric signals[2]. |
| [$^{1}H$,$^{13}C$]-Edited-HSQC | hsqcedetgpsisp2.2 | 32 | 2048x256 | 12.5x146 | 4.7/86 | $J_{CH}$ = 145 Hz |
| [$^{1}H$,$^{1}H$]-NOESY | noesygpphpp | 16 | 2048x256 | 10x10 | 4.7/4.7 | 700 ms mixing time |
| [$^{1}H$,$^{1}H$]-TOCSY | mlevgpph19 | 16 | 2048x256 | 10x10 | 4.7/4.7 | 13 ms or 200 ms mixing times. Distance between nulls = 5.2 ppm |
| [$^{1}H$,$^{13}C$]-HMBC | hmbcgplpndqf | 128 | 2048x240 | 12x92 | 4.7/66 | Long range $J_{CH}$ = 6 Hz |
| [$^{1}H$,$^{13}C$]-HMQC-COSY | h2bcetgpl3 | 328 | 2048x302 | 12x92 | 4.7/66 | $J_{CH}$ min - max = 129 - 190 Hz |

Recovery delay was set to 1.5 – 2.3 s in all experiments.

**Supplementary Table 6. Glycan $^1H/^{13}C$ chemical shifts of CT, $CT_{n-1}$ and HM *N*-glycans (referenced to TSP). Experiments were acquired at 310 K.**

| Residue with residue number | C1/ H1 | C2/ H2 | C3/ H3 | C4/ H4 | C5/ H5 | C6/ H6a;H6b | Acetyl-$CH_3$ | Cα/ Hα | Cβ/ Hβa;Hβb |
|---|---|---|---|---|---|---|---|---|---|
| **CT** | | | | | | | | | |
| Asn | - | - | - | - | - | - | - | 53.8/ 3.98 | 38.0/ 2.94;2.86 |
| GlcNAc **1** | 80.9/ 5.08 | 56.4/ 3.87 | 75.6/ 3.78 | 81.6/ 3.66 | 79.0/ 3.60 | 62.8/ 3.84;3.66 | 24.9/ 2.02 | - | - |
| GlcNAc **2** | 104.0/ 4.66 | 57.8/ 3.79 | 74.7/ 3.80 | 82.2/ 3.74 | 77.3/ 3.63 | 62.8/ 3.89;3.76 | 25.0/ 2.09 | - | - |
| Man **3** | 103.1/ 4.78 | 72.9/ 4.25 | 83.3/ 3.79 | 68.5/ 3.79 | 77.3/ 3.62 | 68.7/ 3.97;3.81 | - | - | - |
| Man **4** | 102.3/ 5.14 | 79.3/ 4.20 | 72.2/ 3.91 | 70.1/ 3.51 | 75.8/ 3.63 | 64.4/ 3.92;3.64 | - | - | - |
| Man **4'** | 99.8/ 4.94 | 79.2/ 4.12 | 72.2/ 3.91 | 70.1/ 3.51 | 75.8/ 3.63 | 64.4/ 3.92;3.64 | - | - | - |
| GlcNAc **5/5'** | 102.3/ 4.60 | 57.6/ 3.75 | 74.9/ 3.75 | 81.5/ 3.74 | 77.5/ 3.59 | 62.8/ 3.99;3.86 | 25.1/ 2.06 | - | - |
| Gal **6/6'** | 105.7/ 4.48 | 73.7/ 3.56 | 75.4/ 3.68 | 71.3/ 3.95 | 76.3/ 3.76 | 63.7/ 3.77;3.77 | - | - | - |
| **$CT_{n-1}$** | | | | | | | | | |
| α-GlcNAc **2** | 93.2/ 5.21 | 56.4/ 3.90 | 72.5/ 3.93 | 82.7/ 3.91 | 77.1/ 3.62 | 62.1/ 3.73;3.73 | 24.6/ 2.06 | - | - |
| β-GlcNAc **2** | 97.7/ 4.72 | 58.9/ 3.71 | 72.5/ 3.93 | 82.7/ 3.91 | 77.1/ 3.62 | 62.8/ 3.79;3.77 | 24.8/ 2.06 | - | - |
| Man **3** | 103.2/ 4.77 | 73.0/ 4.24 | 83.2/ 3.77 | 68.2/ 3.79 | 77.1/ 3.62 | 68.6/ 3.96;3.79 | - | - | - |
| Man **4** | 102.2/ 5.12 | 79.3/ 4.19 | 72.2/ 3.90 | 70.1/ 3.50 | 76.3/ 3.75 | 64.4/ 3.91;3.62 | - | - | - |
| Man **4'** | 99.8/ 4.92 | 79.2/ 4.11 | 72.2/ 3.90 | 70.1/ 3.50 | 75.6/ 3.71 | 64.4/ 3.91;3.62 | - | - | - |
| GlcNAc **5/5'** | 102.2/ 4.58 | 57.6/ 3.74 | 74.9/ 3.72 | 81.4/ 3.72 | 77.4/ 3.57 | 62.8/ 3.98;3.84 | 25.1/ 2.05 | - | - |
| Gal **6/6'** | 105.7/ 4.47 | 73.8/ 3.54 | 75.3/ 3.66 | 71.3/ 3.93 | 78.1/ 3.72 | 63.7/ 3.75;3.75 | - | - | - |
| **HM** | | | | | | | | | |
| Asn | - | - | - | - | - | - | - | 53.8/ 3.99 | 38.0/ 2.93;2.88 |
| GlcNAc **1** | 80.8/ 5.09 | 56.3/ 3.87 | 75.0/ 3.79 | 81.5/ 3.66 | 79.1/ 3.61 | 62.8/ 3.85;3.66 | 24.9/ 2.03 | - | - |
| GlcNAc **2** | 104.0/ 4.63 | 57.8/ 3.79 | 75.0/ 3.79 | 81.9/ 3.74 | 77.2/ 3.61 | 62.8/ 3.89;3.74 | 25.0/ 2.08 | - | - |
| Man **3** | 103.0/ 4.78 | 73.1/ 4.23 | 84.0/ 3.74 | 68.3/ 3.91 | 77.2/ 3.61 | 68.11/ 4.03;3.75 | - | - | - |
| Man **4** | 103.5/ 5.34 | 81.3/ 4.12 | 72.8/ 4.01 | 69.7/ 3.77 | 76.1/ 3.82 | 63.8/ 3.90;3.77 | - | - | - |
| Man **4'** | 102.4/ 4.88 | 81.6/ 3.90 | 81.5/ 3.90 | 67.8/ 3.88 | 74.2/ 3.85 | 68.7/ 3.95;3.80 | - | - | - |
| Man **5** | 103.3/ 5.31 | 81.3/ 4.12 | 72.8/ 4.01 | 69.7/ 3.71 | 76.1/ 3.82 | 63.8/ 3.90;3.77 | - | - | - |
| Man **5'** | 103.3/ 5.41 | 81.3/ 4.12 | 72.9/ 3.96 | 69.7/ 3.71 | 76.1/ 3.82 | 63.8/ 3.90;3.77 | - | - | - |
| Man **5''** | 100.9/ 5.14 | 81.3/ 4.03 | 72.9/ 3.96 | 69.7/ 3.71 | 75.5/ 3.71 | 63.8/ 3.90;3.77 | - | - | - |
| Man **6/6'/6''** | 105.0/ 5.07 | 72.8/ 4.09 | 73.2/ 3.86 | 69.6/ 3.67 | 76.0/ 3.78 | 63.8/ 3.90;3.77 | - | - | - |

**Supplementary Table 7. Example of shell script for NMR data processing with NMRPipe**

```
#!/bin/csh

foreach spec (1 3 5 7 9 11 12 13 14 15)
cd spec/$spec
bruk2pipe -in ./ser \
  -bad 0.0 -ext -aswap -AMX -decim 8330.66666666667 -dspfvs 20
-grpdly 67.9856719970703  \
  -xN               512  -yN               256  \
  -xT               256  -yT               128  \
  -xMODE            DQD  -yMODE    States-TPPI  \
  -xSW         2400.768  -ySW         3320.053  \
  -xOBS         600.230  -yOBS         150.931  \
  -xCAR          0.8578  -yCAR         19.7253  \
  -xLAB              1H  -yLAB             13C  \
  -ndim               2  -aq2D            TPPI  \
  -out ./test.fid -verb -ov

nmrPipe -in test.fid \
| nmrPipe  -fn EM  -lb 1.0 -c 1.0                       \
| nmrPipe  -fn ZF -auto                                 \
| nmrPipe  -fn FT -auto                                 \
| nmrPipe  -fn PS -p0 62 -p1 0 -di -verb                \
| nmrPipe  -fn BASE -nw 5 -nl 0% 2%  98% 100%           \
| nmrPipe  -fn CS -rs 0Hz -sw                           \
| nmrPipe  -fn EXT -x1 -0.3ppm -xn 2.4ppm -sw           \
| nmrPipe  -fn TP                                       \
| nmrPipe  -fn EM  -lb 1.0 -c 1.0                       \
| nmrPipe  -fn ZF -auto                                 \
| nmrPipe  -fn FT -auto                                 \
| nmrPipe  -fn PS -p0 0 -p1 0 -di -verb                 \
| nmrPipe  -fn BASE -nw 2 -nl 0% 2%  98% 100%           \
| nmrPipe  -fn CS -rs 0Hz -sw                           \
   -ov -out ../test-$spec.ft2
rm test.fid
cd ..
cd ..
end
```

**Supplementary Table 8. Dissociation constants $K_D$, on- and off-rate constants $k_{on}$ and $k_{off}$ for CT, Rituximab-Fc, HM and $Ca^{2+}$ binding to $EndoS2_{E186L}$ and $EndoBT_{D312A/E314L}$ in the presence of constant additives from fitting a two-state binding model to methyl-TROSY titration data using TITAN algorithm.**

| N° | Enzyme | Ligand | Additives[1] | $K_D$ (μM) | $k_{off}$ ($s^{-1}$) | $k_{on}$ ($M^{-1}s^{-1}$) |
|---|---|---|---|---|---|---|
| 1 | $EndoS2_{E186L}$ | CT | $CaCl_2$ | 111.2 ± 9.7 | 11.7 ± 1.9 | $1.1x10^5 ± 2.0x10^4$ |
| 2 | | Fc | $CaCl_2$ | 3.8 ± 1.0 | 4.5 ± 1.8 | $1.2x10^6 ± 5.7x10^5$ |
| 3 | | $CaCl_2$ | CT | 4100 ± 300 | - | - |
| 4 | | $CaCl_2$ | Fc | - | - | - |
| 5 | $EndoBT_{D312A/E314L}$ | HM | $CaCl_2$ | 0.4 ± 0.3 | 2.4 ± 1.1 | $5.5x10^6 ± 4.8x10^5$ |
| 6 | $EndoBT_{D312A/E314L}$ | CT | - | >755 (if present) | - | - |

[1]Samples containing additives were prepared at saturating concentrations and diluted a maximum of 10% during the course of the titrations. Final additive concentrations were: 20 mM $CaCl_2$, 830 μM CT, 56 μM Fc or 72 μM Fc aglycan in samples containing $EndoS2_{E186L}$, and 291mM $CaCl_2$ in samples containing $EndoBT_{D312A/E314L}$.

**Supplementary Note 1**

**Structural analysis of EndoS and EndoS2 in complex with the Fc region of Rituximab in solution by small angle X-ray scattering**

To study the overall structure of EndoS and EndoS2 in complex with the Fc region of IgG1 in solution, we first performed in-line size-exclusion chromatography small-angle X-ray scattering (SEC-SAXS) (Supplementary Figs. 1 and 6; Supplementary Table 2). We purified the catalytically inactive EndoS$_{E235A}$ and EndoS2$_{E186L}$ variants and the Fc region of Rituximab, a chimeric IgG1 used to treat B cell lymphoma, by papain digestion. We mixed the EndoS$_{E235A}$ or EndoS2 $_{E186L}$ with the Fc region substrate in a 1:5 ratio and we isolated the enzyme-substrate complexes (EndoS$_{E235A}$-Fc and EndoS2$_{E186L}$-Fc) from the free enzymes and substrate by SEC chromatography and conducted SAXS analyses of the individual proteins and the complexes (Supplementary Fig. 6). Comparison of the $R_g$ shows an increase when EndoS$_{E235A}$-Fc ($R_g$ = 53.6 Å) and EndoS2$_{E186L}$-Fc ($R_g$ = 52.2 Å) complexes are formed compared to the unliganded EndoS$_{E235A}$ ($R_g$ = 42.9 Å) and EndoS2$_{E186L}$ ($R_g$ = 38.7 Å) (Supplementary Table 2). In addition, examination of the *P(r)*-distribution shows that the maximum dimension, *Dmax*, also increases when the enzyme-substrate is formed, from 148 Å to 181 Å in the case of EndoS and from 125 Å to 176 Å in the case of EndoS2. The resulting *P(r)* function profile of both enzyme-substrate complexes exhibit a unimodal distribution of real-spaces distances that is characteristic of a globular protein, while the *P(r)* function profiles of the unbound EndoS and EndoS2 are bimodal consistent with the overall "V" shape of the crystal structures of both enzymes (Supplementary Fig. 6). Thus, the change from a bimodal to a monomodal distribution in the enzyme-substrate complexes strongly suggests that the Fc might bind to the cleft of the EndoS or EndoS2, promoting a more compact, globular state. The experimental determination of the EndoS$_{E235A}$-Fc complex MW[3] (Supplementary Table 2) shows a 36.7% and a 26.5 % discrepancy to the calculated MW of the enzyme-substrate complex with 1:1 and 2:1

stoichiometries, respectively. In the case of EndoS2$_{E186L}$-Fc complex, the experimental determination of the MW from the SAXS data shows a 40 % and 1.1 % discrepancy to the calculated MW of the enzyme-substrate complex with 1:1 and 2:1 stoichiometries (Supplementary Table 2). These results, together with the fact that the $R_g$ values are decreasing across the SEC peak of the enzyme-substrate complexes (Supplementary Fig. 6) indicate that a mixture of complexes with different stoichiometries might be present in that SEC peak. Therefore, we used OLIGOMER[4] to calculate the volume fractions of the complexes with different stoichiometry contributing to the scattering profile. In the case of the mixture EndoS$_{E235A}$-Fc, the results showed that the enzyme-substrate 2:1 and 1:1 complexes contributed 67% and 29% volume fractions, respectively, to the scattering. Similar ratio of volume fractions was found for the EndoS2$_{E186L}$-Fc mixture (Supplementary Table 2).

**Supplementary Note 2**

**Extended NMR results**

To shine more light on the interaction of endo-β-*N*-acetylglucosaminidases (ENGases) with their natural ligands, we have employed NMR-based chemical shift perturbation (CSP) experiments[5]. CSPs are usually extracted from uniformly $^{2}H$,$^{15}N$-labeled proteins applying $^{1}H$,$^{15}N$ Transverse Relaxation Optimized Spectroscopy (TROSY) Heteronuclear Single Quantum Coherence (HSQC) spectroscopy. However, at a molecular weight of 97 kDa $^{1}H$,$^{15}N$ HSQC-TROSY spectra of EndoS2 suffered from incomplete deuterium-hydrogen back exchange of backbone $H^{N}$. The difficulty becomes evident when comparing $^{1}H$,$^{15}N$ HSQC-TROSY spectra of EndoS2 with those acquired from EndoBT-3987 glycosidase[6,7], the latter having a molecular weight of "only" 47 kDa (Supplementary Fig. 16a,b). We have previously shown that deuterium-hydrogen back exchange is crucial to extract reliable information from $^{1}H$,$^{15}N$ HSQC-TROSY spectra[8]. Yet, all attempts to develop an efficient unfolding-refolding protocol for complete deuterium-hydrogen back exchange of backbone $H^{N}$ in ENGases failed. Therefore, we turned to specific $^{13}C$ side chain methyl group labeling strategies[1,9,10], which enabled the acquisition of $^{1}H$,$^{13}C$ Heteronuclear Multiple Quantum Correlation (HMQC) spectra, also known as methyl-TROSY[11]. Such approach grants the extraction of precise CSPs upon, e.g., ligand binding and provides experimental access to binding thermodynamics and kinetics[1,5]. Knowledge of the corresponding on- and off-rate constants would provide a link to the conformational changes associated with substrate binding as observed by cryoEM and H/D exchange,[2] assisting the mechanistic understanding of ENGases.

NMR lineshape analysis has been used in numerous instances for the evaluation of chemical shift titration experiments, yielding quantitative descriptions of chemical exchange processes such as conformational equilibria or binding of ligands to receptor proteins[12]. A novel approach for analyzing CSP titration data from HSQC- and HMQC-type 2D NMR

spectra engages direct quantum mechanical simulation of a given pulse sequence, as it is implemented in the TITAN (TITration ANalysis) software package[13]. Here, we have used TITAN to analyze the kinetics of binding of EndoS2 to its substrate CT, Rituximab-Fc, the reaction product $CT_{n-1}$ and $Ca^{2+}$ ions. The interaction of the related EndoBT-3987 with HM was also studied and used as comparison.

The isotope labeling scheme selected for this study was [$U$-$^{15}N$,$^{2}H$] $MIL^{proS}V^{proS}$ [$^{13}C$,$^{1}H_3$]-methyl labelling (or simply MILV). Isotopically labeled ENGases were the catalytically inactive $EndoS2_{E186L}$ and $EndoBT_{D312A/E314L}$, and their corresponding wildtype EndoS2 and EndoBT species[2,6]. This labeling scheme rendered methyl-TROSY spectra with little to no signal overlapping and excellent signal-to-noise (Supplementary Fig. 16c,d). In addition, the selected methyl probes are uniformly distributed around the putative glycan binding pockets in both enzymes, granting the extraction of CSPs upon ligand titration (Fig. 6a; Supplementary Fig. 17a). $^{1}H$,$^{15}N$-TRACT experiments[14] were used to estimate rotational correlation times $\tau_c$, affording values of 45.0 ns and 21.8 ns for $EndoS2_{E186L}$ and $EndoBT_{D312A/E314}$, respectively, in good agreement with their corresponding molecular weights (Supplementary Fig. 22). Taken together with the good signal dispersion observed in NMR spectra, $\tau_c$ values evidence the presence of folded protein in solution enabling protein-ligand interaction studies.

At the present time no resonance assignment is available for ENGases. Therefore, we avoided any attempt to use CSPs for epitope mapping. Instead, we focused our efforts on the quantification of the overall thermodynamic and kinetic binding parameters for the interaction with selected ligands. For such goal it is enough to identify a subset NMR signals reporting for the binding event of interest. Here, methyl signals associated with methyl groups in the vicinity of the glycan binding site were identified according to the following two-step exclusion criteria: a) Signals must show CSPs between the wild-type species and the corresponding enzymatically

inactive mutants (*i.e.* EndoS2 *vs* EndoS2$_{E186L}$ and EndoBT-3987 *vs* EndoBT-3987$_{D312A/E314L}$). Such mutations were designed to render inactive enzymes while retaining the protein folding and protein dynamics associated with ligand recognition, as previously disclosed by crystallographic and H/D exchange MS studies[2,6]. Therefore, observed CSPs can be linked to changes localized in the vicinity of the carbohydrate binding pockets, even in the event of long-range effects[15,16]. b) Signals must exhibit CSPs after saturation with CT, CT$_{n-1}$ and Rituximab-Fc (for EndoS2$_{E186L}$), or after saturation with HM (for EndoBT-3987$_{D312A/E314L}$). A visual description of ligands used in this study can be found in Fig. 6C, Supplementary Figs. 17a and 20). Fulfilling these criteria grants the selection of CSPs associated to ligand recognition, irrespective of whether they originate from the presence of a ligand in the vicinity, or from long-range effects. Only such methyl signals were selected for lineshape analysis with TITAN. Detailed examples from EndoS2$_{E186L}$ and EndoBT$_{D312A/E314L}$ can be scrutinized in Fig. 6B and in Supplementary Figs. 17c, respectively. Following this methodology, we could safely identify 10 methyl signals reporting for the binding of EndoS2$_{E186L}$ to CT, CT$_{n-1}$ and Rituximab-Fc, and another 10 signals reporting for the interaction of EndoBT$_{D312A/E314L}$ with HM (signals 1-10 in Supplementary Fig. 23 and signals 11-20 in Supplementary Fig. 24, respectively). EndoS2 also possesses a calcium-binding pocket located in the β-sandwich (BS) domain (Fig. 6a). Signals exhibiting CSPs greater than 2 standard deviations upon titration with calcium chloride were selected as reporters for calcium binding.

**Extraction of kinetic and thermodynamic parameters from methyl-TROSY spectra**

Titration of catalytically dead ENGases with carbohydrates CT, CT$_{n-1}$ or Rituximab-Fc produced CSPs in the slow-exchange regime in the NMR time-scale, leading to varying peak intensities during titration (Supplementary Figs. 25-30). On the other hand, titration of EndoS2 with $CaCl_2$ produced CSPs in the fast-exchange regime, which translates into shifts in peak

positions (Supplementary Fig. 31). Methyl-TROSY based titrations in slow exchange were subjected to lineshape analysis with TITAN software package. For each titration point, the corresponding methyl-TROSY spectrum was imported into MATLAB and then analyzed with the TITAN algorithm, applying a two-state ligand binding model. Quantitative lineshape analysis yielded dissociation constants $K_D$ and on- and off-rate constants $k_{on}$ and $k_{off}$. A representative example of lineshape fitting can be found in Fig. 6D.

In the main text, we have described the kinetics of binding of EndoS2 to its substrate CT, Rituximab-Fc and the reaction product $CT_{n-1}$. Here, we focus on the effect of calcium in the interaction of EndoS2 with CT and Rituximab-Fc. Calcium binding produced similar CSPs in EndoS2 and $EndoS2_{E186L}$ species with virtually identical dissociation constants $K_D$, suggesting no influence of the mutation E186L on calcium recognition (Table 1, entries 6,7, Supplementary Fig. 32). It is worth noting however, that some signals showed significant CSPs during the titration of $EndoS2_{E186L}$ with CT, $CT_{n-1}$, Rituximab-Fc and $CaCl_2$. Such promiscuity in CSPs suggests the presence of alternative calcium-binding sites or a cross-talking mechanism between the GH and BS domains, as it has already been proposed from functional studies with chimeric ENGases.[2] Here, we did not attempt any further analysis on such signals. Instead, they will be the subject of a future study, whenever a spectrum assignment is available for EndoS2. Titrations of CT and Rituximab-Fc were repeated in the presence of saturating concentrations of $CaCl_2$, producing almost identical results as in its absence. Similarly, no significant differences were observed for the titration of $CaCl_2$ in the presence of saturating concentrations of CT. No interaction between $EndoS2_{D186L}$ and $CaCl_2$ was observed when the enzyme was pre-saturated with Rituximab-Fc. This observation is in good agreement with the cryoEM structure reported in this study, where no calcium ion is bound to EndoS2 in the complex EndoS2-Fc (Supplementary Table 8). It appears that calcium is not directly involved in the recognition of IgGs by EndoS2, although its exact role remains yet to be identified.

At this point, it is instructive to compare dissociation constants, on- and off-rate constants from ENGases with diverse specificities. EndoBT-3987 is a globular ENGase secreted by the gram-negative symbiont *Bacteroides thetaiotaomicron,* which displays high specificity for mammalian HM-type *N*-glycans. Titrations of $EndoBT_{D312A/E314L}$ with HM in the absence and presence of $CaCl_2$ afforded dissociation constant, on- and off-rate constants in the same order of magnitude as those previously disclosed for $EndoS2_{E186L}$ binding Rituximab-Fc. As expected, no affinity was observed for CT glycan, even at ~750 μM glycan concentrations. (Table 1, entry 6, lineshape fittings for the titration of HM can be scrutinized in Supplementary Figs. 33 and 34).

Our study was complemented with the extraction of the catalytic rate $k_{cat}$ and the Michaelis-Menten constant $K_M$ from the catalytic cleavage of complex-type carbohydrates from Rituximab-Fc by EndoS2. Kinetic parameters were approximated using the Michaelis-Menten equation (5) (steady-state kinetics), hypothesizing a nearly constant concentration of enzyme-substrate complex during the reaction course. This assumption was based on the two orders of magnitude $K_D$ difference observed for the binding of EndoS2 to Fc as compared to the reaction product $CT_{n-1}$ ($K_D$ = 3.1 μM *vs* $K_D$ = 150.7 μM, respectively). Concentration of enzymatically released carbohydrates was measured from $^1$H spectra. Background signals were readily suppressed by a perfect CPMG $T_2$-filter, which is a relaxation filter that avoids *J*-modulation[17] (Supplementary Fig. 18). Cleaved carbohydrate was quantified from the integral of the composite signal at 2.08–2.10 ppm, which reports for *N*-acetylglucosamine and *N*-acetylneuraminic acid moieties (Supplementary Fig. 19a). Reaction time-courses were measured to close-to-reaction completion for five Rituximab-Fc initial concentrations (Supplementary Fig. 19b). The reaction rate was calculated from the fitting of initial velocity rates ($\upsilon_0$) to the equation of Michaelis-Menten (5) (Fig. 6e). Under these conditions, a catalytic rate $k_{cat}$ of 5.9 $s^{-1}$ and a Michaelis-Menten constant $K_M$ of 26.7 μM were observed. It is

important to remark that $K_M$ resembles $K_D$ only when $k_{off}$ >> $k_{cat}$, according to Eq. 6. In this study this condition is not met ($k_{off}$ = 4.0 $s^{-1}$ ≈ $k_{cat}$ = 5.9 $s^{-1}$), indicating that the system is not in rapid equilibrium. Nevertheless, care has to be taken when combining results obtained from different EndoS2 species (wt and E186L), because non-identical protein-carbohydrate interactions can result in slightly different kinetic parameters. In conclusion, this study shows for the first time an overview of enzyme kinetics followed by EndoS2. We hope this information will be key for the future development of ENGases with improved activity and selectivity.

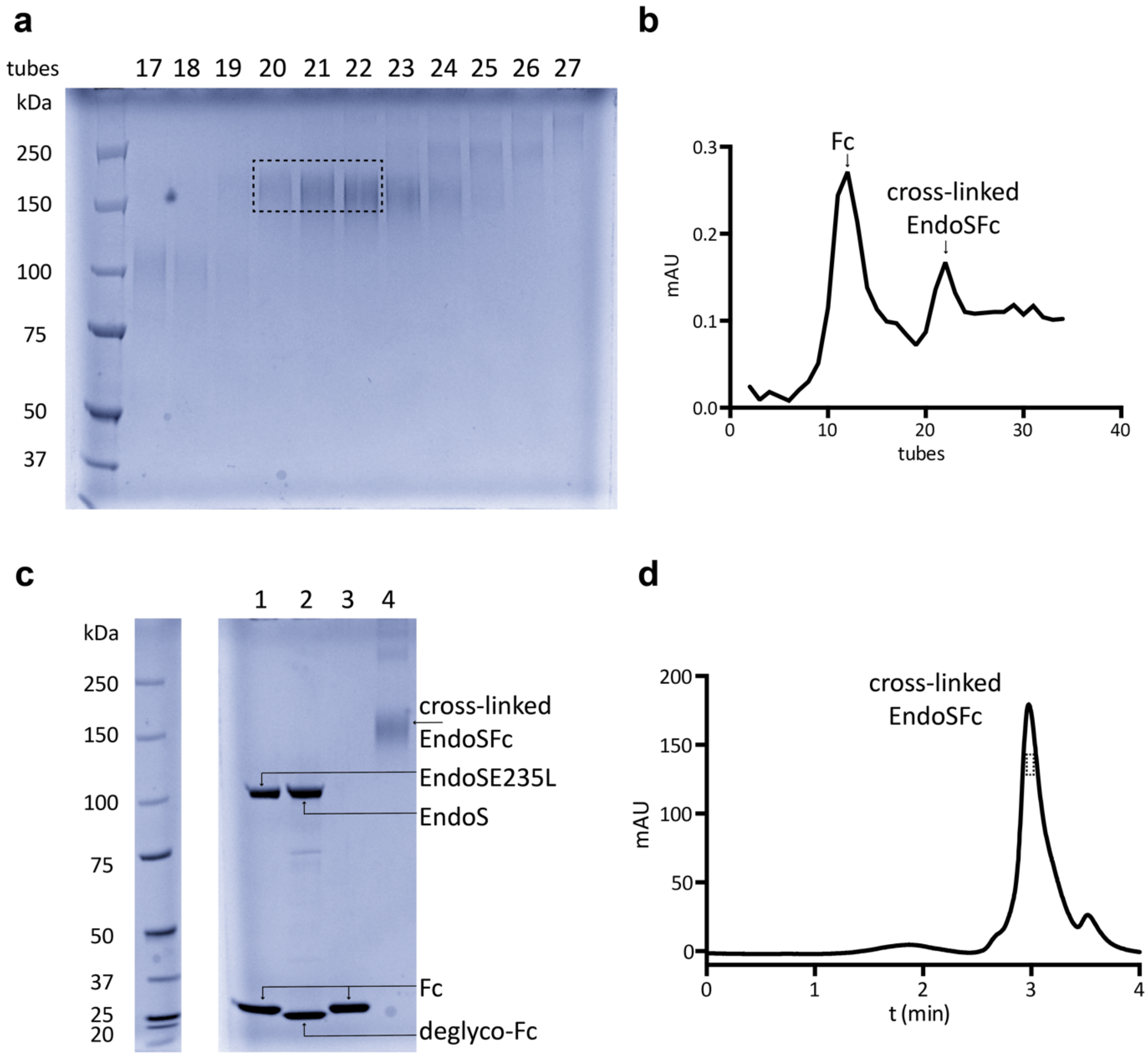


**Supplementary Figure 1 | Purification of the cross-linked EndoS$_{E235A}$-Fc complex. a** SDS-PAGE analysis and **b** protein absorbance of fractions obtained by GraFix centrifugation. **c** SDS-PAGE showing the fractions of the EndoS$_{E235A}$-Fc complex concentrated from GraFix centrifugation and the enzyme and substrate components. **d** Superdex 200 Increase 10/300 GL profile showing the purified cross-linked EndoS$_{E235A}$-Fc complex. This experiment was repeated six times independently with similar results.

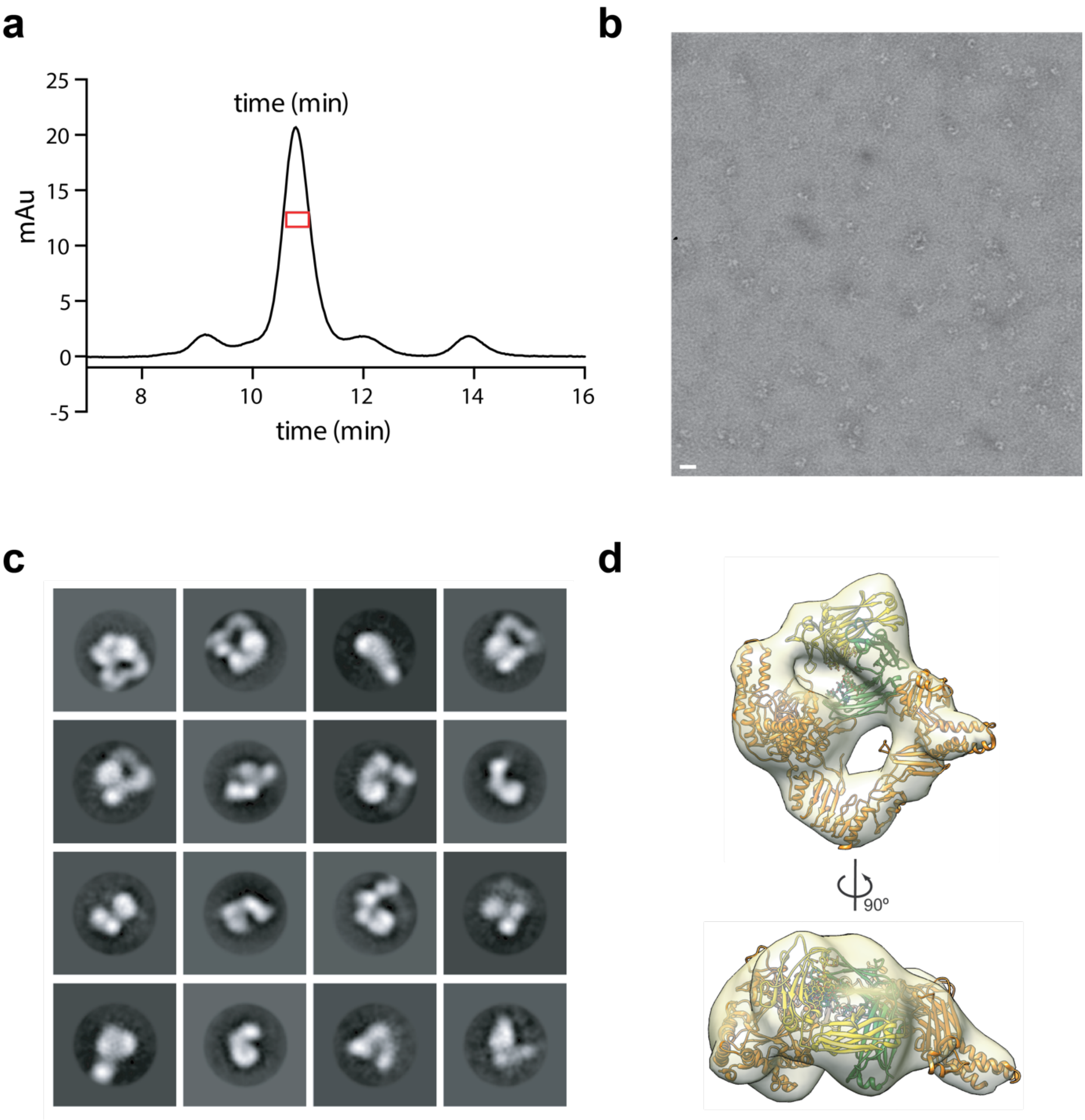


**Supplementary Figure 2 | The EndoS$_{E235A}$-Fc complex by NS. a** Superdex 200 Increase 10/300 GL profile showing the purified cross-linked EndoS$_{E235A}$-Fc complex. **b** Micrograph detail. **c** Representative 2D classes (scale bar, 100 Å). **d** 3D model reconstruction of EndoS$_{E235A}$-Fc complex. The EndoS$_{E235A}$ (PDB code 6EN3) and Fc (PDB code 1H3X) X-ray crystal structures are fitted into the model using Chimera. This experiment was repeated three times independently with similar results.

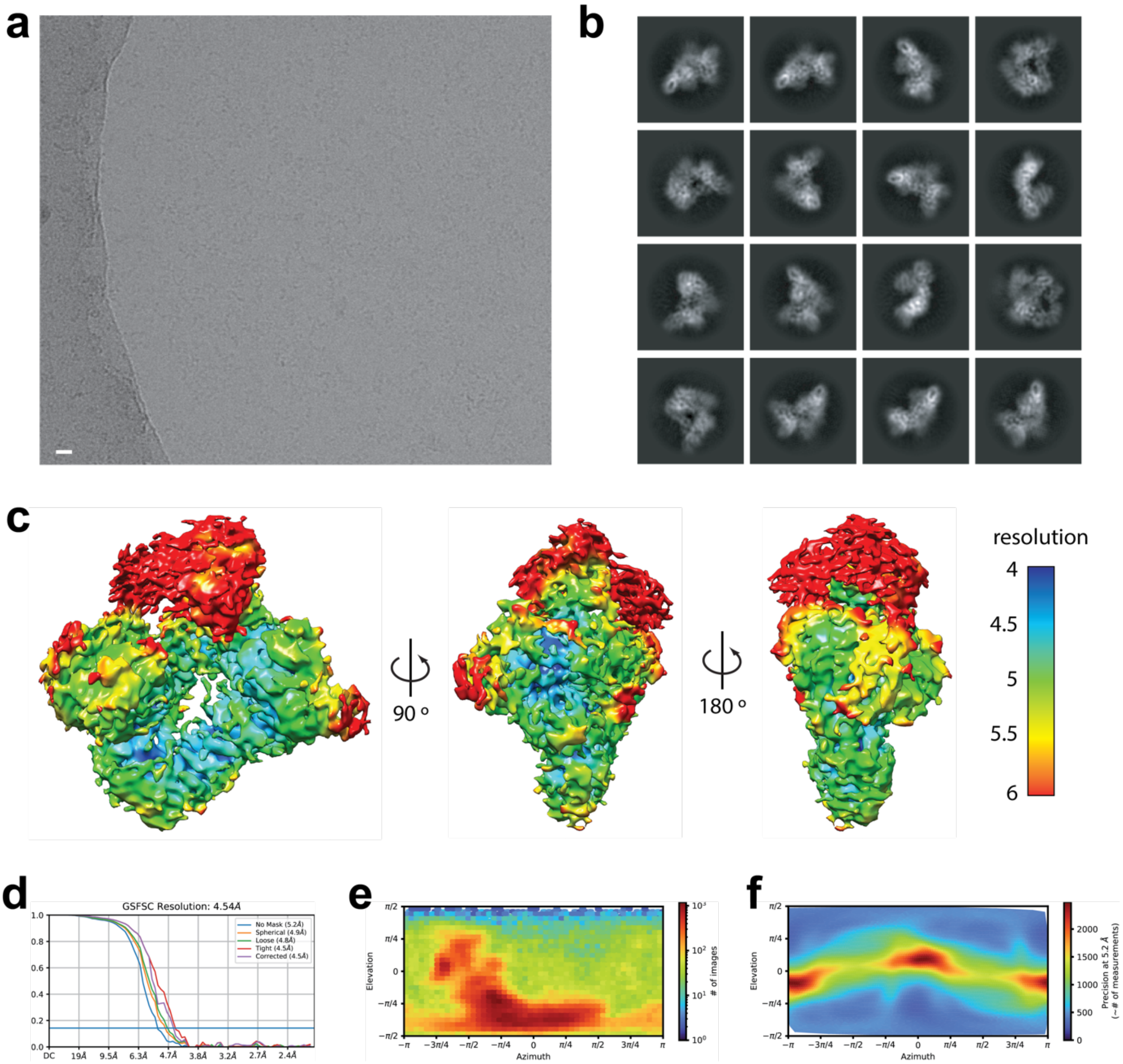


**Supplementary Figure 3 | The EndoS$_{E235A}$-Fc complex structure by cryoEM. a** Micrograph detail (scale bar, 100 Å). **b** Representative 2D classes. **c** Overall view of the single-particle reconstructions of EndoS$_{E235A}$-Fc complex using Cryosparc. **d** FSC, **e** direction distribution and **f** posterior precision plots of the EndoS$_{E235A}$-Fc complex.

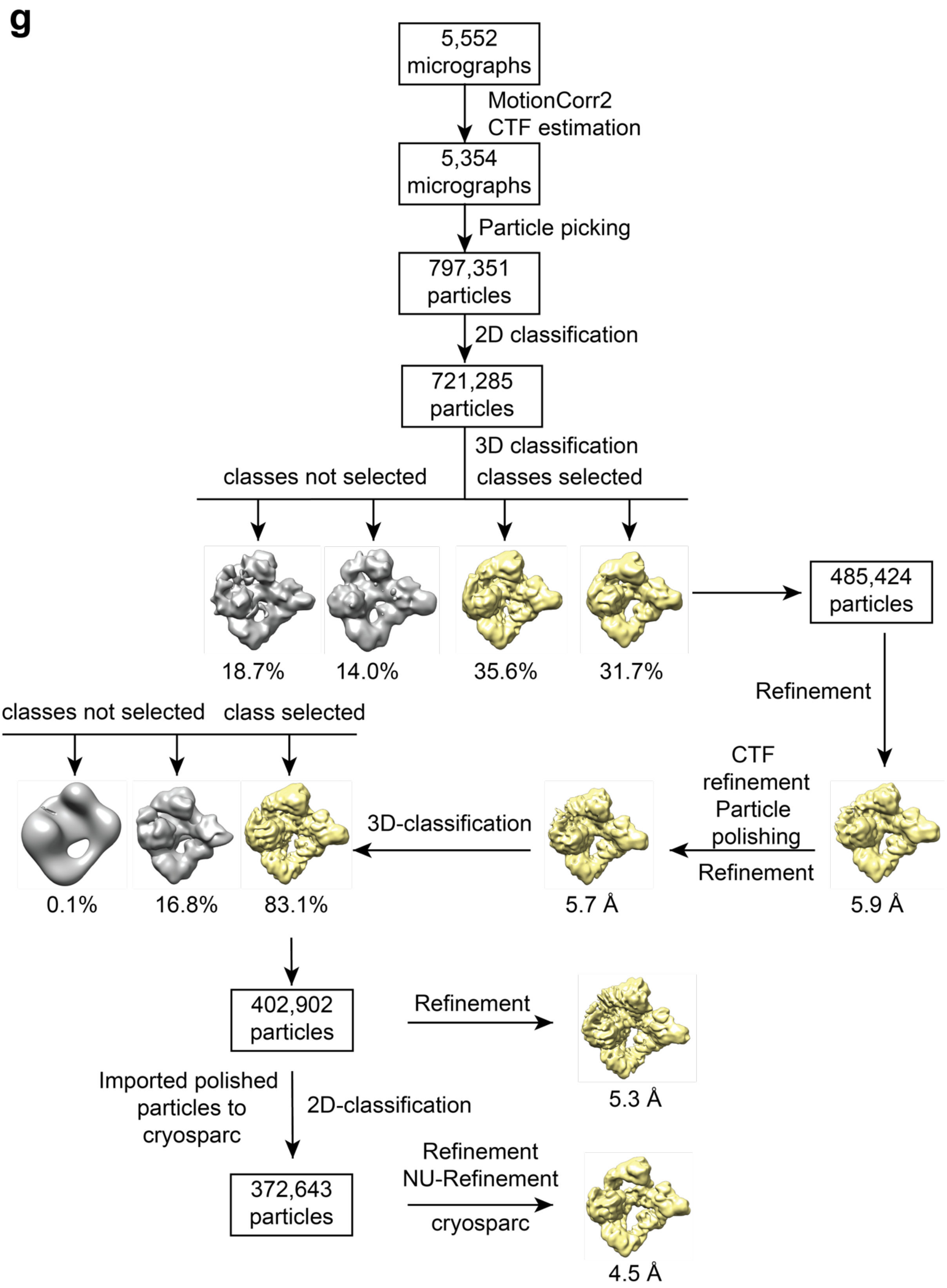


**Supplementary Figure 3 | continued. g** Flow chart for cyoEM data processing of EndoS$_{E235A}$-Fc. The data processing was performed using RELION unless stated otherwise.

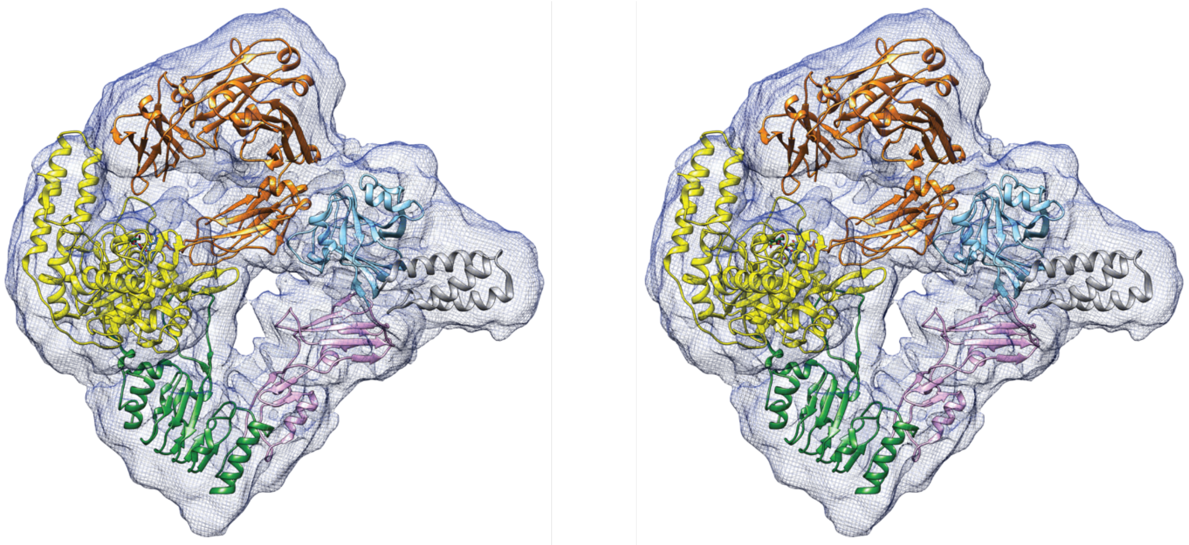

**Supplementary Figure 4 |** Stereo views of the $EndoS_{E235A}$-Fc complex models and cryosparc map.

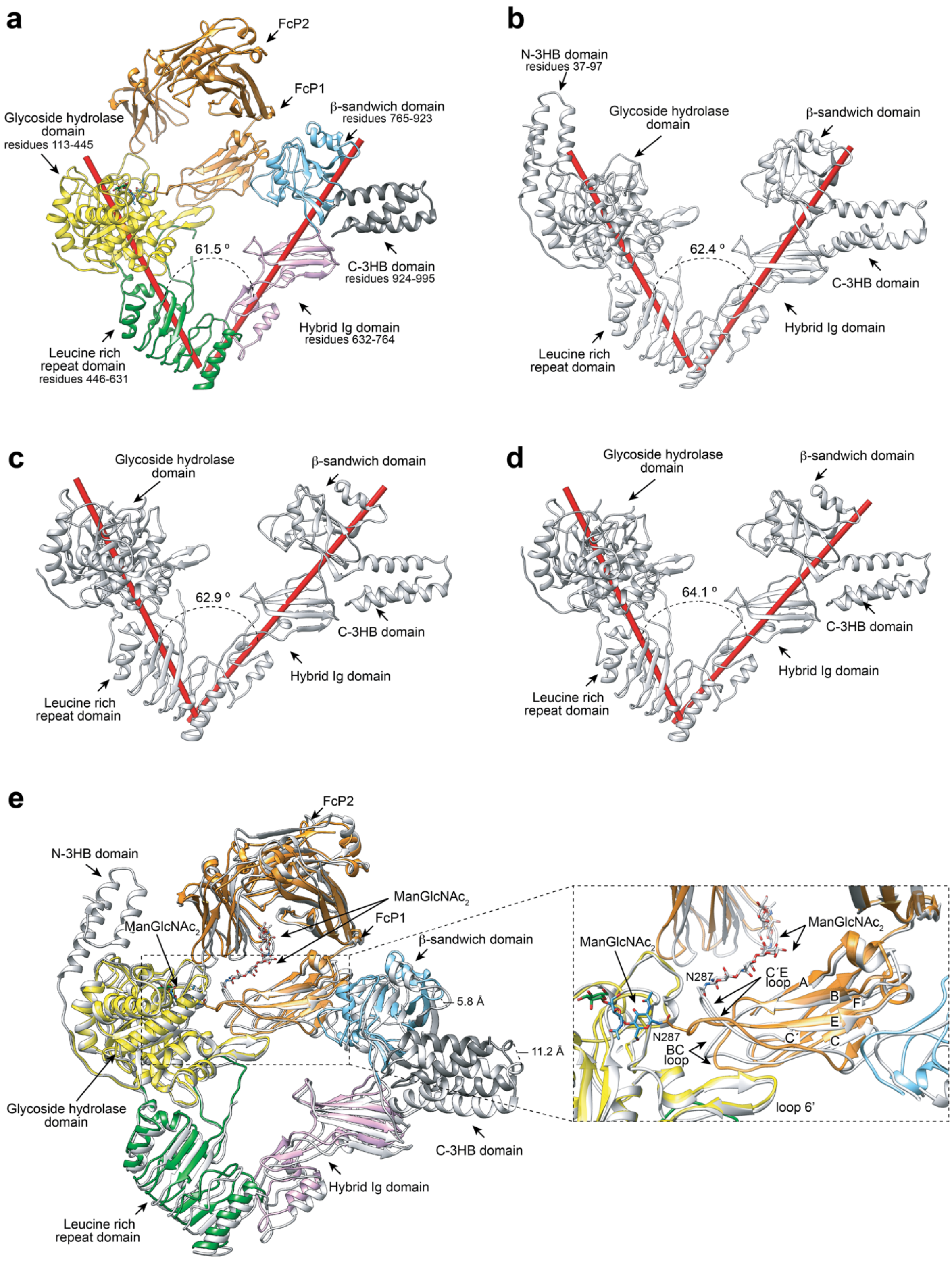


**Supplementary Figure 5 | Structural comparison of unliganded EndoS and EndoS$_{E235A}$-Fc complex structures.** cryoEM structure of EndoS$_{E235A}$-Fc complex (PDB code 8A64) (**a**) and X-ray crystal structures of EndoS$_{D233A/E235L}$-CT (PDB code 6EN3) (**b**), ΔN-3HB-EndoS (PDB code 4NUY) (**c**) and ΔN-3HB-EndoS$_{D233Q}$ (PDB code 4NUZ) (**d**). The red cylinders represent the axes passing through the mass center of the glycoside hydrolase and leucine rich repeat domains of EndoS (residues 111-605) and the hybrid Ig, the β-sandwich and C-3HB

domains of EndoS (residues 606-987). **e** Structural superposition of the X-ray crystal structures of $EndoS_{D233A/E235L}$-CT (PDB code 6EN3) and the Fc region of IgG1 (PDB code 1H3X) and the cryoEM structure of $EndoS_{E235A}$-Fc complex (PDB code 8A64).

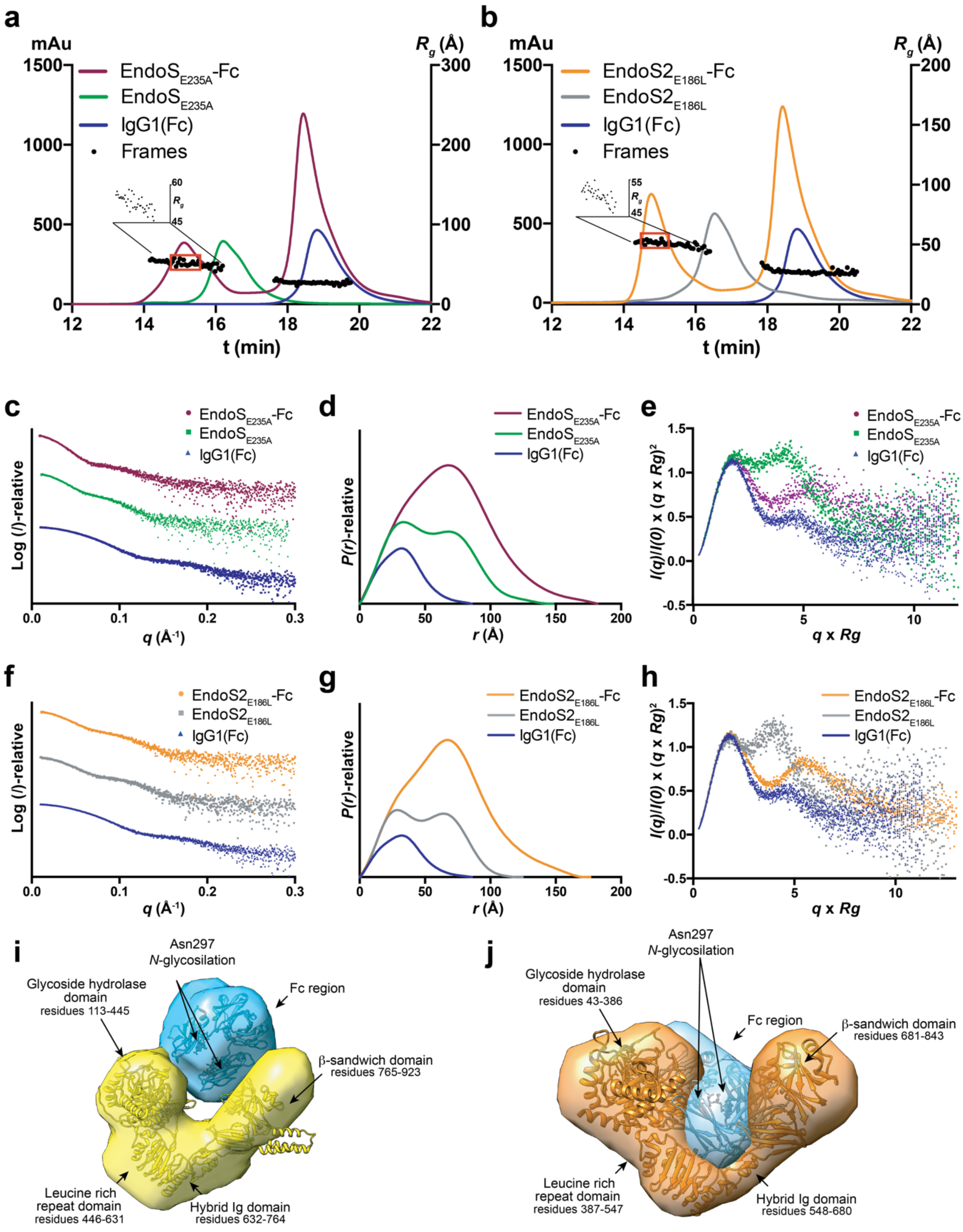


**Supplementary Figure 6 | SAXS analysis of the EndoS$_{E235A}$-Fc and EndoS2$_{E186L}$-Fc complexes. a** In-line SEC-SAXS profile of EndoS$_{E235A}$, IgG1(Fc) and EndoS$_{E235A}$-Fc complex. The frames used for data analysis of the complex are highlighted with a red square. **b** SEC-SAXS profile of EndoS2$_{E186L}$, IgG1(Fc) and EndoS2$_{E186L}$-Fc complex. The frames used for data analysis of the complex are highlighted with a red square. **c** SAXS scattering curve of EndoS$_{E235A}$, IgG1(Fc) and EndoS$_{E235A}$-Fc. **d** *P(r)* functions distributions of EndoS$_{E235A}$, IgG1(Fc) and EndoS$_{E235A}$-Fc complex**. e** Normalized Kratky plot EndoS$_{E235A}$, IgG1(Fc) and EndoS$_{E235A}$-Fc complex. **f** SAXS scattering curve of EndoS2$_{E186E}$, IgG1(Fc) and EndoS2$_{E186E}$-

Fc. **g** *P(r)* functions distributions of EndoS2$_{E186E}$, IgG1(Fc) and EndoS2$_{E186A}$-Fc complex**.** **h** Normalized Kratky plot EndoS2$_{E186A}$, IgG1(Fc) and EndoS2$_{E186A}$-Fc complex. **i** *Ab initio* modelling with MONSA of the envelope of EndoS$_{E235A}$-FC and superimposition of EndoS (yellow) and Fc region of IgGa (blue) X-ray crystal structures. **j** *Ab initio* modelling with MONSA of the envelope of EndoS2$_{E186A}$-FC and superimposition of EndoS2 (orange) and Fc region of IgGa (blue) X-ray crystal structures.

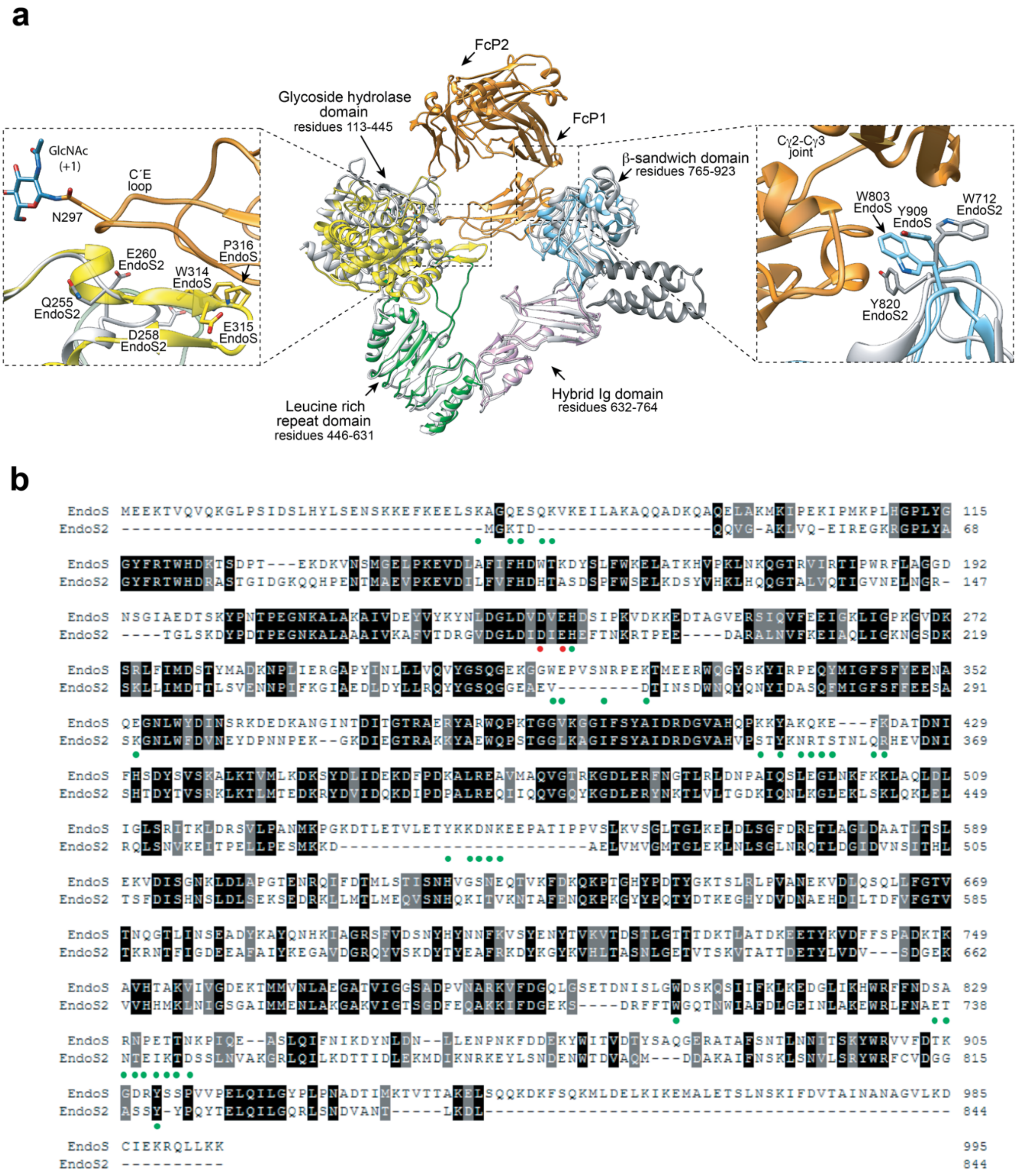


**Supplementary Figure 7 | Comparison of EndoS and EndoS2. a** Cartoon representation of EndoS (colored by domains, PDB code 6EN3) and EndoS2 (grey, PDB code 6MDS) X-ray crystal structures. **b** Structure-based amino acid sequence alignment of EndoS and EndoS2. The catalytic residues and the residues that interact with the Fc region are highlighted in red and green, respectively.

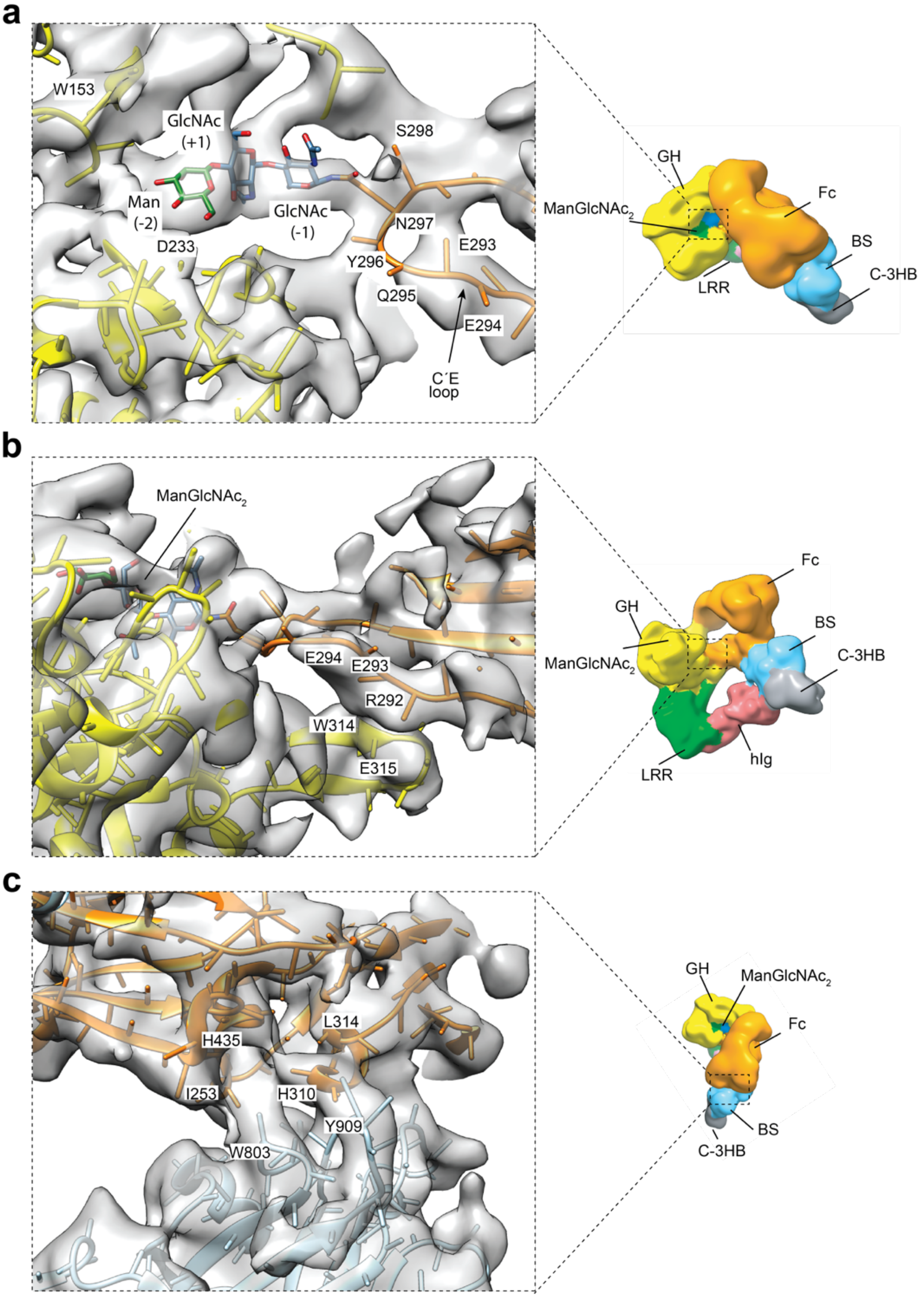


**Supplementary Figure 8 | Electron density map of the Fc binding site of EndoS.** (**a**,**b**) Two views of the electron density map interaction interface between the GH domain of EndoS and Fc region. Three residues of the CT *N*-glycan of N297 of IgG1 (ManGlcNAc$_2$) are modelled in one of the possible conformations found in the binding site of EndoS. (**c**) View of Cγ2-Cγ3 joint region of the Fc region interacting with the β-sandwich domain of EndoS.

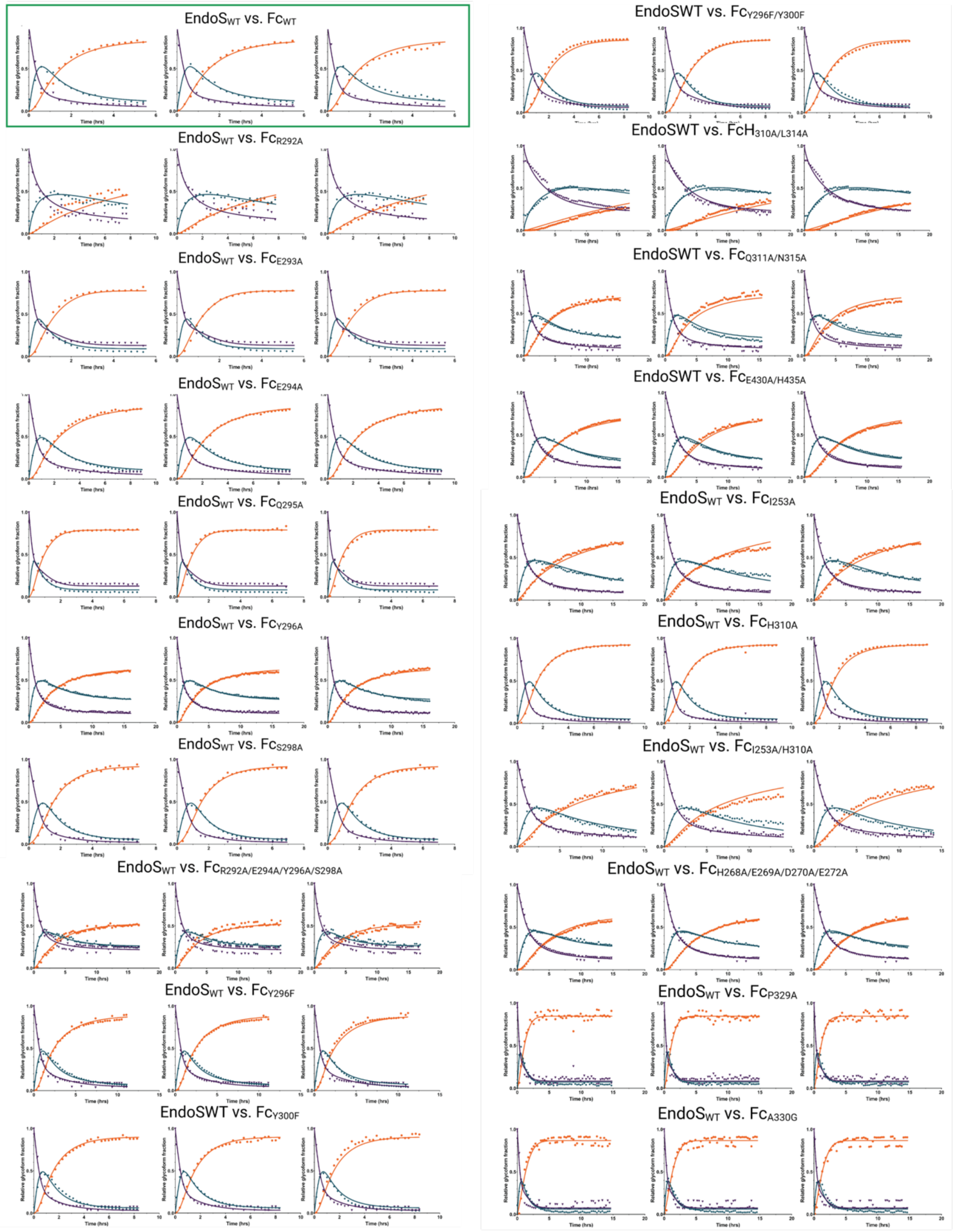


**Supplementary Figure 9 | Kinetic traces for the single hydrolytic activity assays of the EndoS and Fc region mutants.**

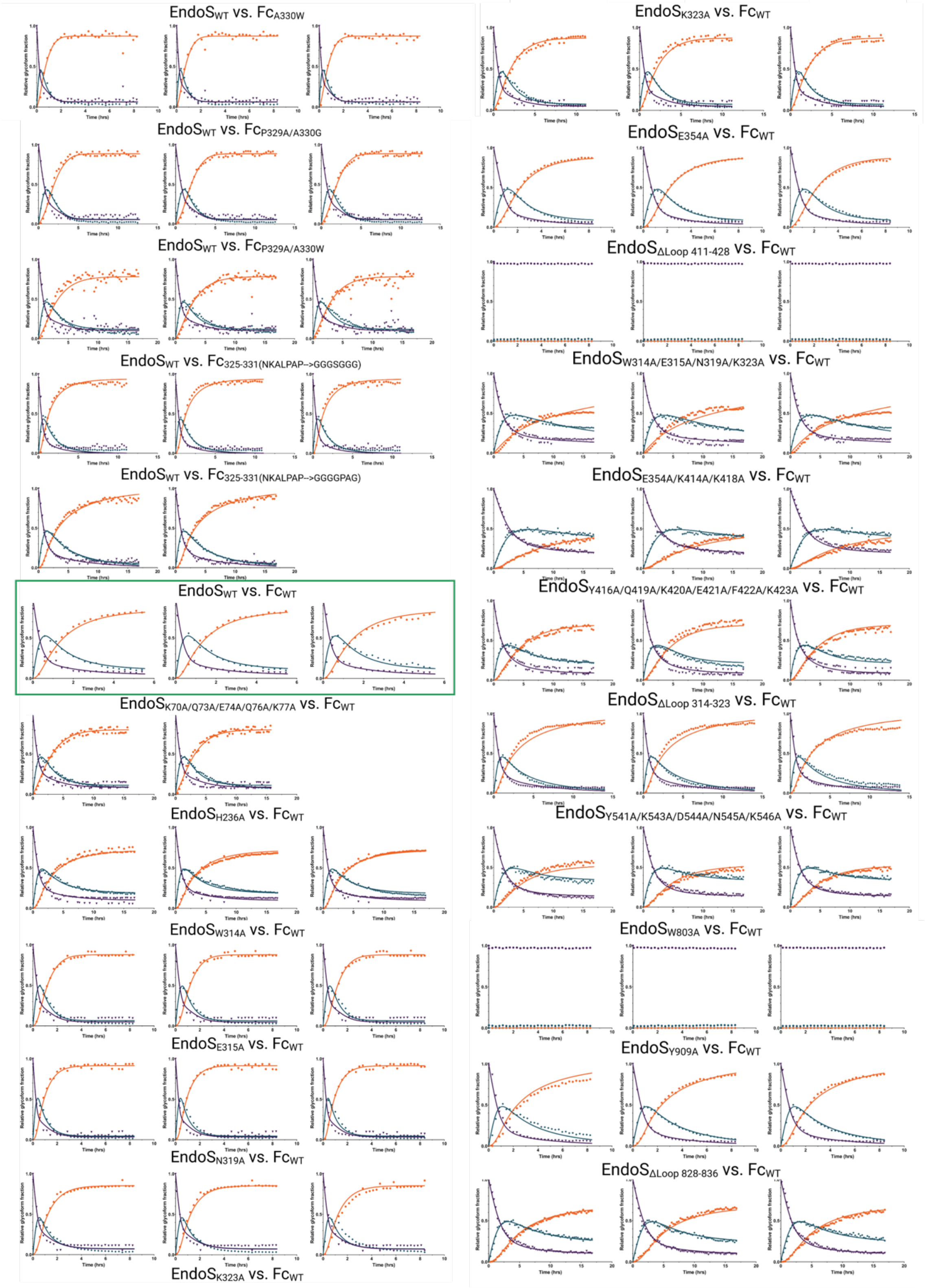


**Supplementary Figure 9 | continued.**

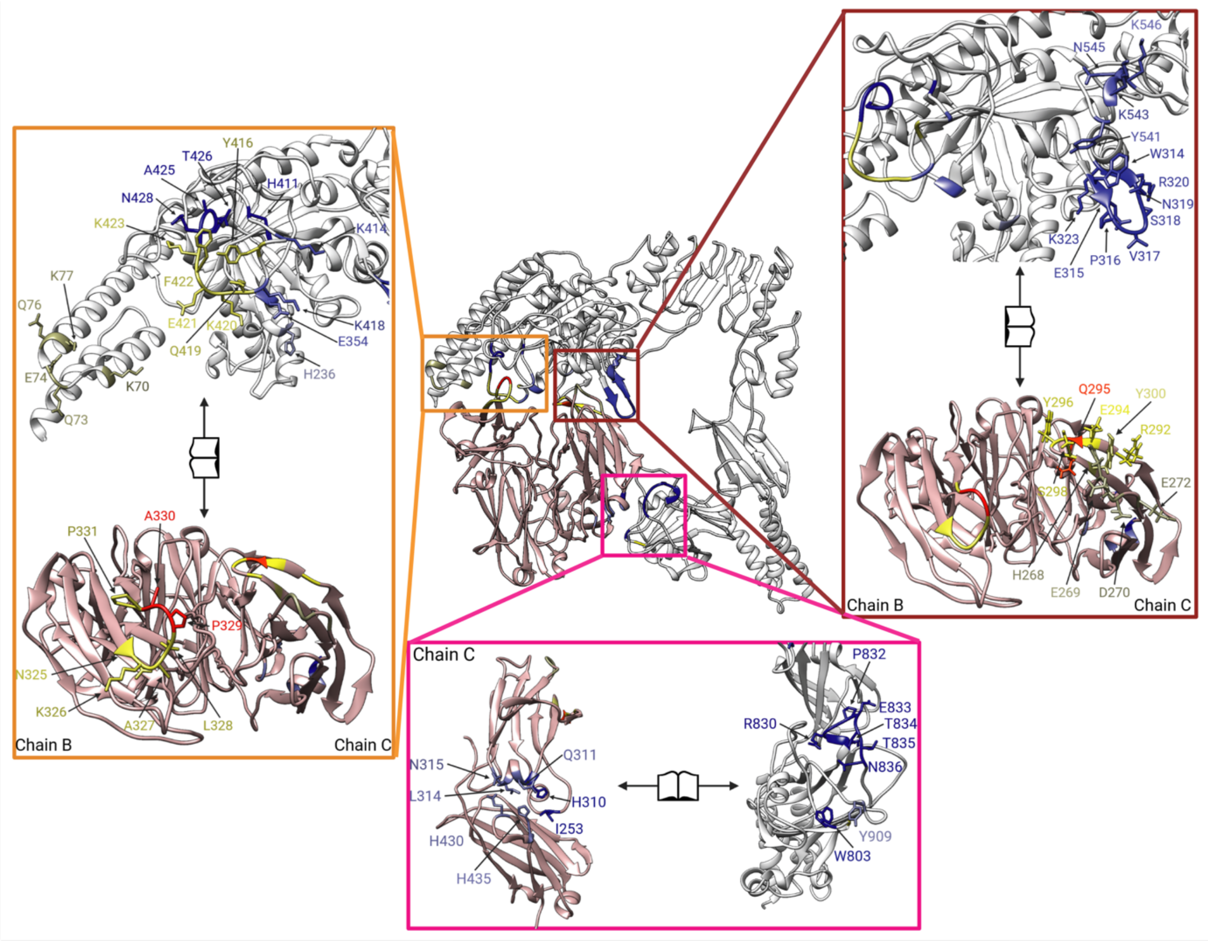


**Supplementary Figure 10 | Interaction interface between the EndoS and the Fc region of IgG1.** Close up panels showing the interface between (i) the N-3HB of EndoS and the FcP2 domain, (ii) the GH of EndoS and (iii) the FcP1 domains and the β-sandwich of EndoS and the FcP1 domains.

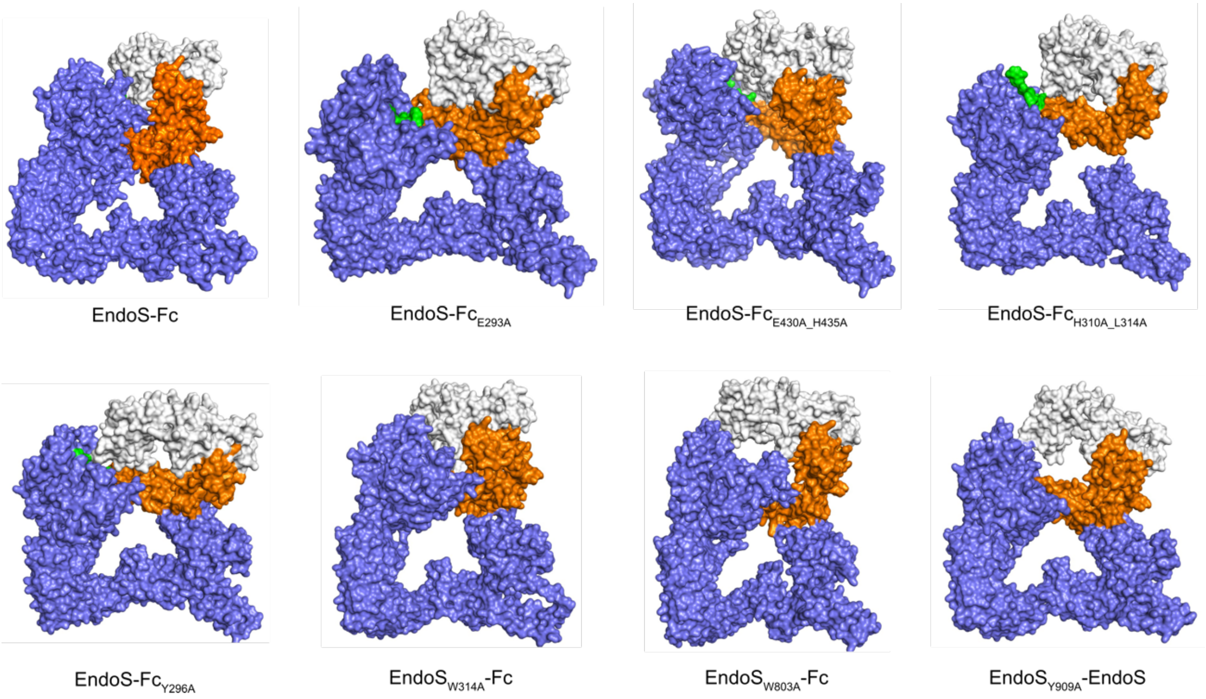


**Supplementary Figure 11 | Average structure derived from 1 µs MD simulations of the different complexes simulated in this work.** Only C$\alpha$ atoms of the proteins are considered for these calculations. The EndoS protein is shown as a purple surface and the Fc as white and orange surfaces and the sugar in green.

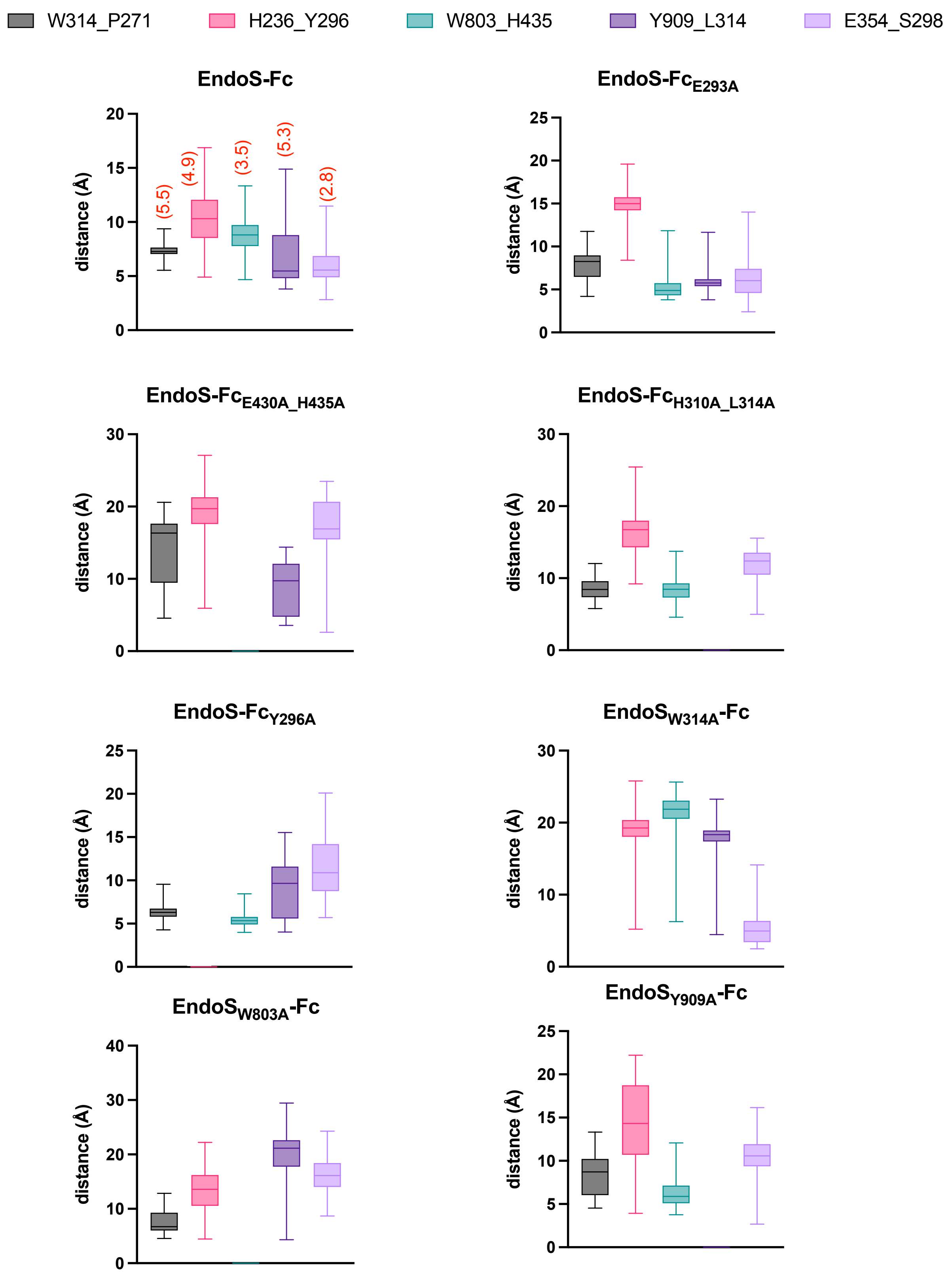


**Supplementary Figure 12 | Average distances derived from 1µs MD simulations between different residues of EndoS and Fc for the simulated complexes.** Box-and-whisker plots, showing the minimum, maximum, median, 1st quartile, and 3rd quartile bars, as well as the interquartile range, based on $n$ = 250 evenly distributed values obtained from the MD

trajectories. For aromatic residues and Pro, the center of the aromatic ring and the center of the ring are considered, respectively, for these calculations. For L314, S298 and E354 residues, the Cδ, the OH group, and the oxygens of the carboxylate are considered, respectively, for the calculations. Cut-off distance: 5.5 Å –π-π stating and CH-π –, and 3.5 Å –hydrogen bond–. Errors are given as SD. The numbers in red and in parentheses are the experimental values found in the cryo-EM structure. The dashed lines represent the distance values found in the cryo-EM structure of EndoS-Fc.

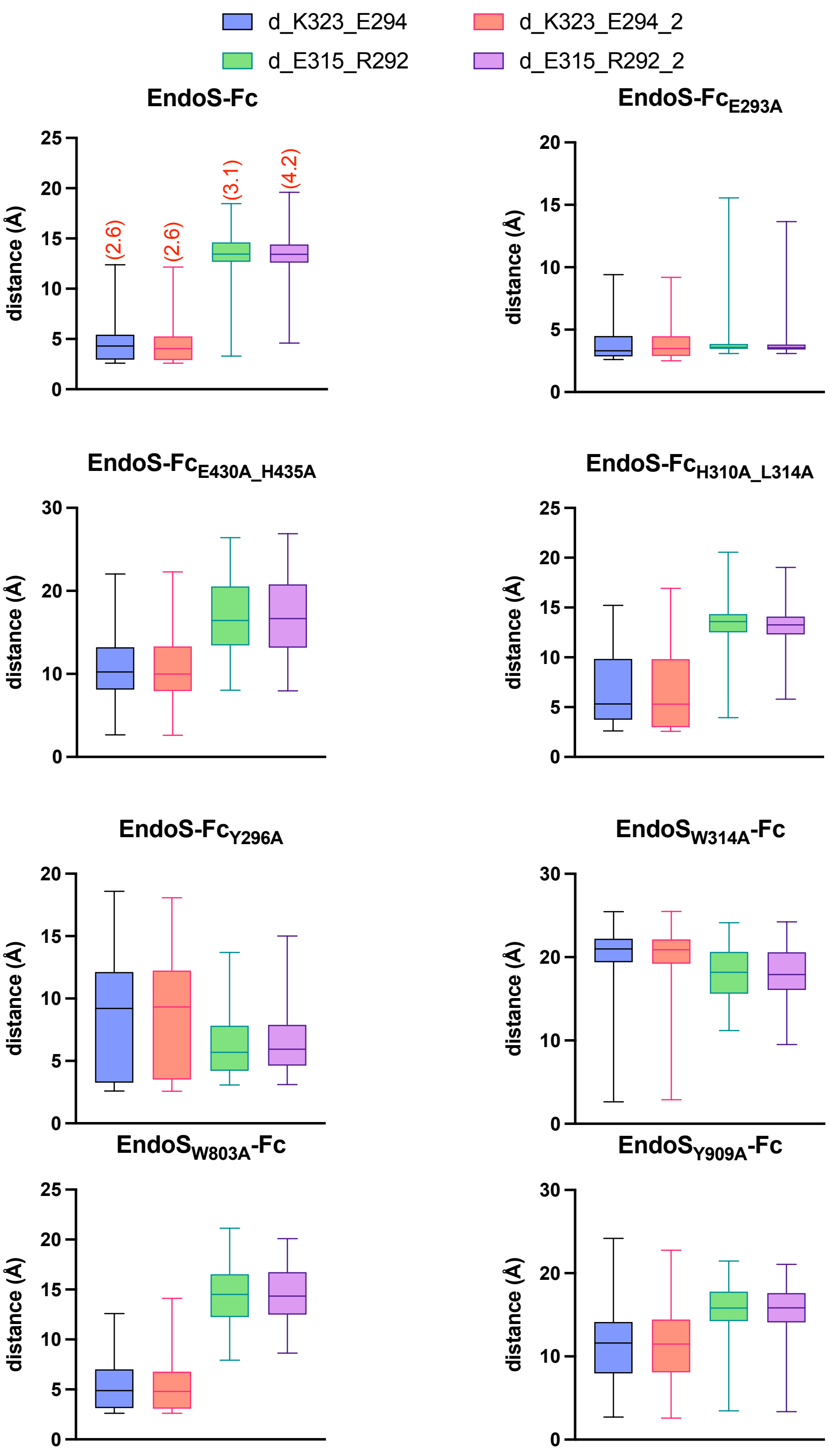


**Supplementary Figure 13 | Average distances derived from 1μs MD simulations for two salt-bridge interactions between EndoS and Fc for the simulated complexes.** Box-and-whisker plots, showing the minimum, maximum, median, 1st quartile, and 3rd quartile bars, as well as the interquartile range, based on $n$ = 250 evenly distributed values obtained from the MD trajectories. The side chain nitrogen of Lys and the carbon atom of the guanidino group of Arg, together with the oxygens of the carboxylate of Glu residues are considered for these calculations (cut-off distance 4.5 Å). Errors are given as SD. The numbers in red and in parentheses are the experimental values found in the cryoEM structure. The dashed lines represent the distance values found in the cryoEM structure of EndoS-Fc.

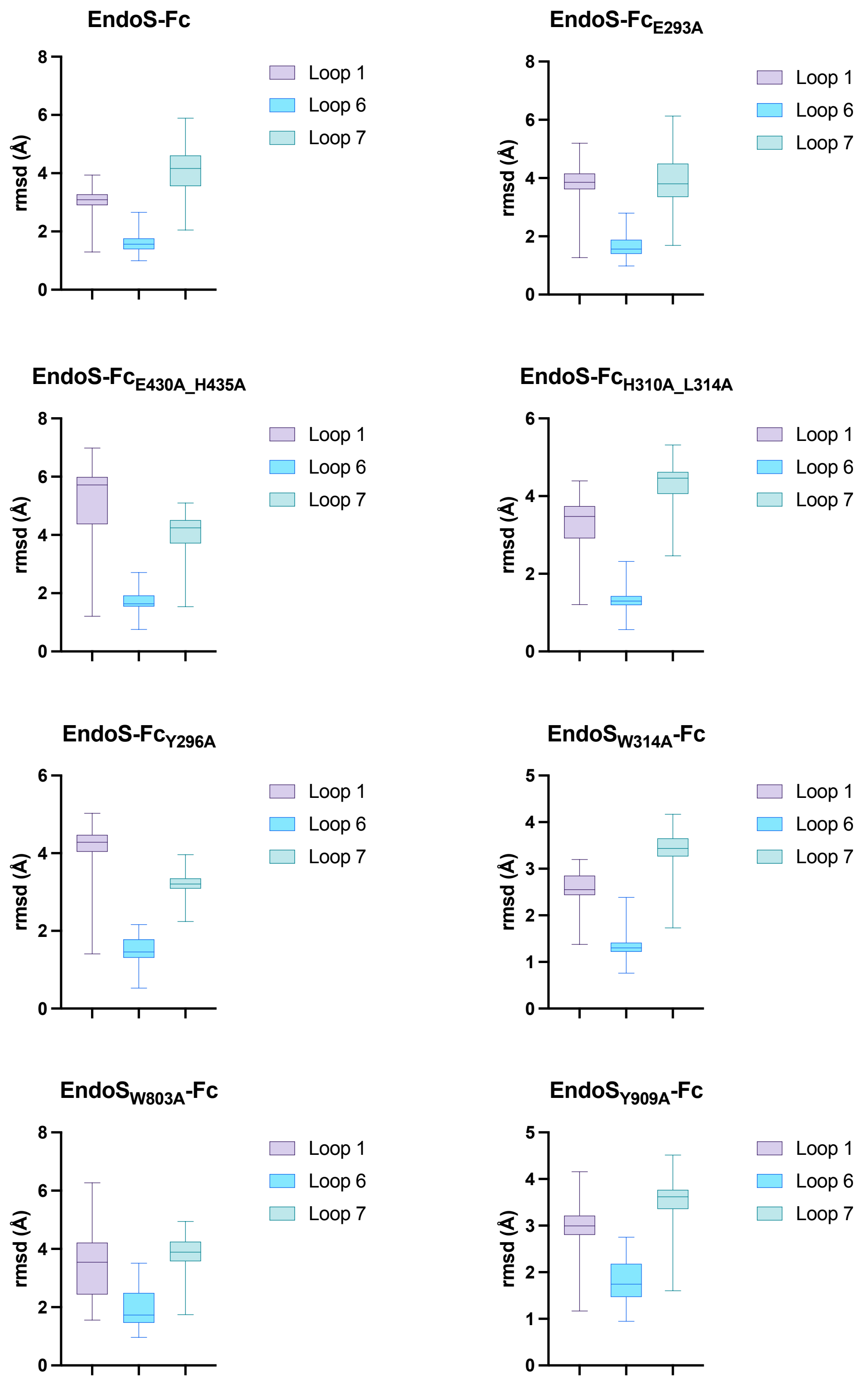


**Supplementary Figure 14 | Root-mean-square deviation (r.m.s.d.) value (±SD) of the peptide backbone of loops 1, 6 and 7 derived from 1 µs MD simulations for the simulated complexes is shown.** Box-and-whisker plots, showing the minimum, maximum, median, 1st quartile, and 3rd quartile bars, as well as the interquartile range, based on *n* = 250 evenly distributed values obtained from the MD trajectories.

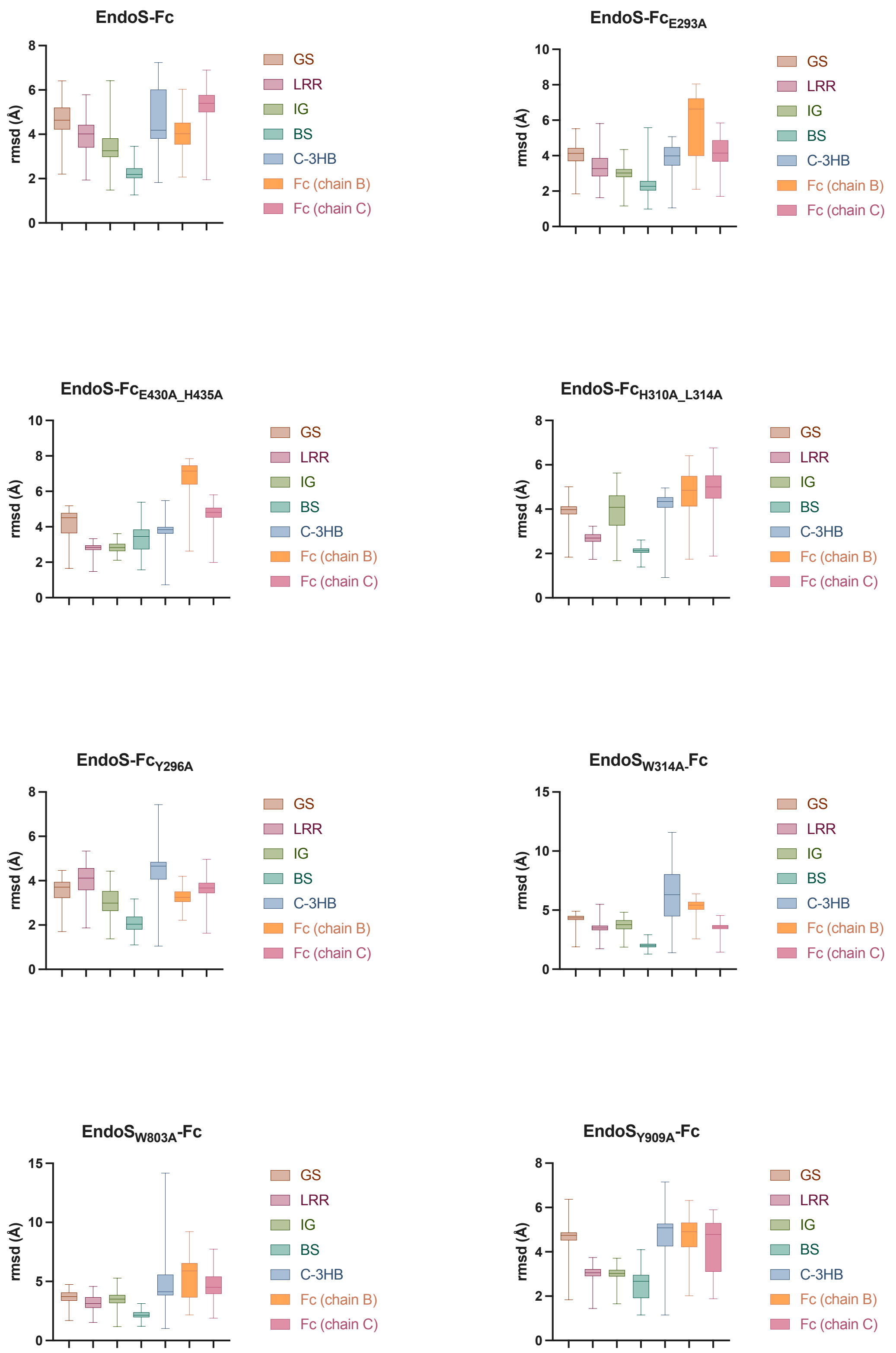


**Supplementary Figure 15 | r.m.s.d. value (±SD) of the peptide backbone of the different domains derived from 1 μs MD simulations for the simulated complexes is shown.** Box-and-whisker plots, showing the minimum, maximum, median, 1st quartile, and 3rd quartile bars, as well as the interquartile range, based on *n* = 250 evenly distributed values obtained.

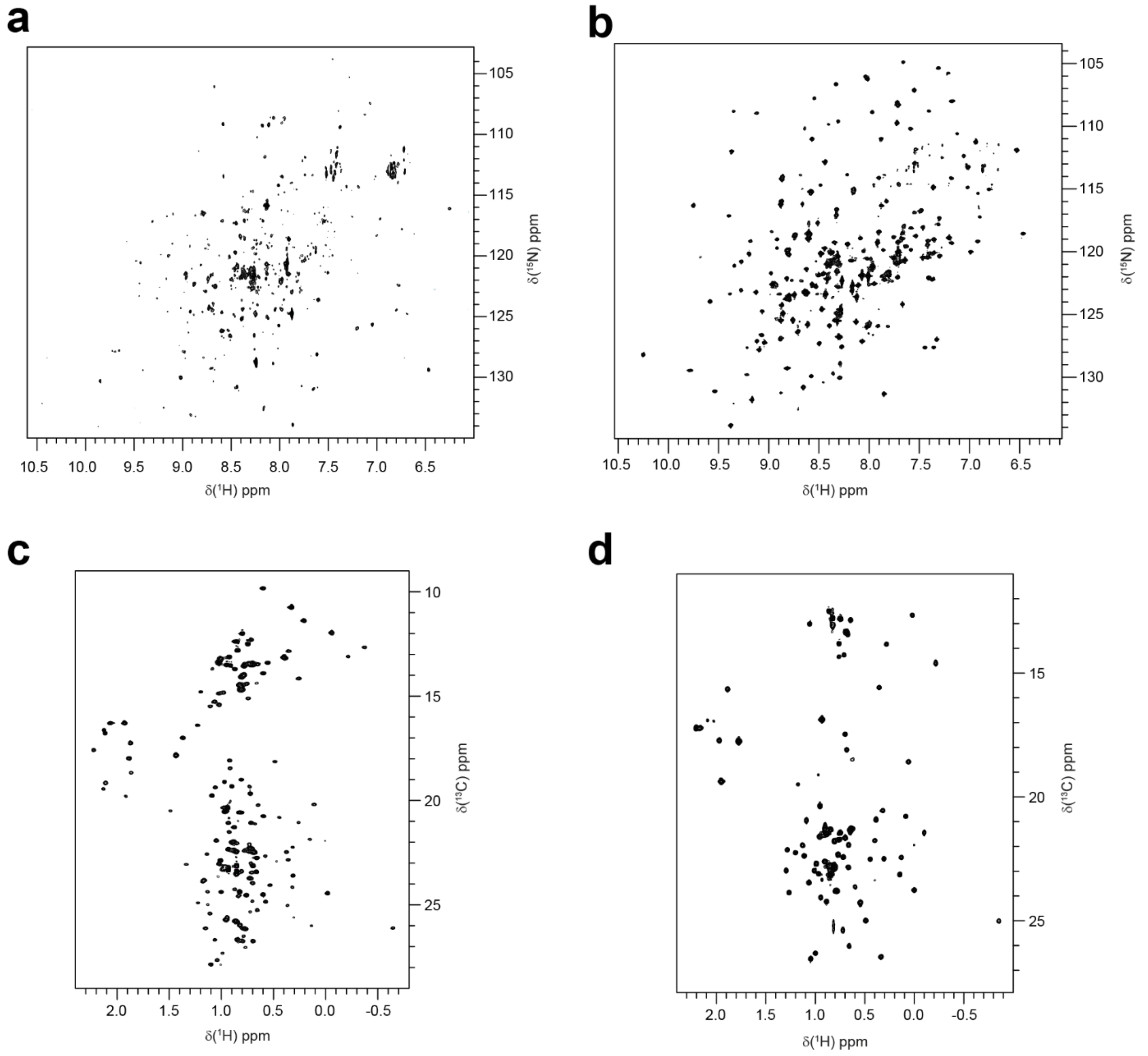


**Supplementary Figure 16 | Quality of NMR spectra of EndoS2$_{E186L}$ and EndoBT$_{D312A/E314L}$ samples.** $^{1}H$,$^{15}N$ HSQC-TROSY spectra of [$U$-$^{15}N$,$^{2}H$] MILV-[$^{13}C$,$^{1}H_3$]-methyl labeled sample of (**a**) EndoS2$_{E186L}$ (97 kDa) at 71.5 μM protein concentration, and (**b**) EndoBT$_{D312A/E314L}$ (47 kDa) at 74.4 μM protein concentration. Methyl-TROSY spectra of (**c**) EndoS2$_{E186L}$ and (**d**) EndoBT$_{D312A/E314L}$ at 51.5 μM and 100 μM protein concentrations are shown as comparison. All experiments were acquired at 298 K and 600 MHz.

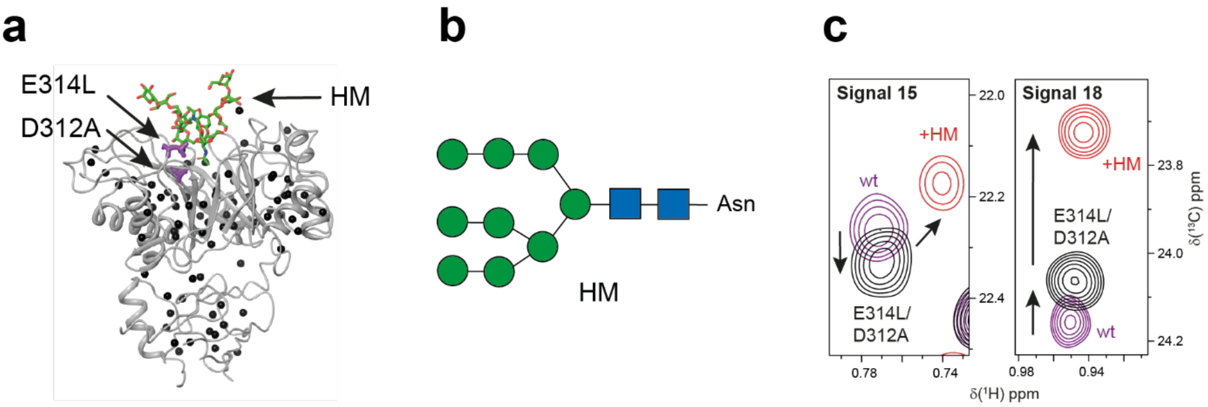


**Supplementary Figure 17 | NMR titration of EndoBT-3987$_{D312A/E314}$ with high mannose-type *N*-glycan HM**. **a** Crystal structure of EndoBT-3987$_{D312A/E314}$ bound to HM glycan (PDB 6TCV). Mutated amino acids D312A and D314L are colored in purple, and HM glycan is shown as green sticks. Isotopically labeled methyl groups are indicated as black spheres. **b** Cartoon representation of HM. **c** Superimposition of selected regions of methyl-TROSY spectra showing the CSPs upon mutagenesis or at saturation concentrations of HM. Signals from EndoBT-3987 are shown in violet, and from EndoBT-3987$_{D312A/E314L}$ in the absence (black) and in the presence of saturating concentrations of HM (red). Arrows indicate the extent of CSPs observed between the different protein states. All experiments were acquired at 600 MHz and 298 K.

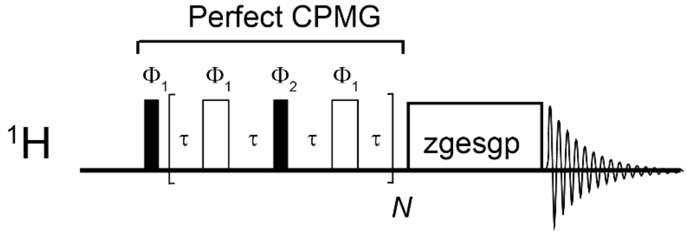


**Supplementary Figure 18 | Scheme showing the perfect CPMG building block**. The initial 90° pulse in the standard Bruker pulse sequence zgesgp was substituted by the sequence shown above. Echo time echo time $\tau$ was set to 0.3 ms and $N$ = 33. Phase cycling during this block was as follows: $\Phi_1$ = x; $\Phi_2$ = y.

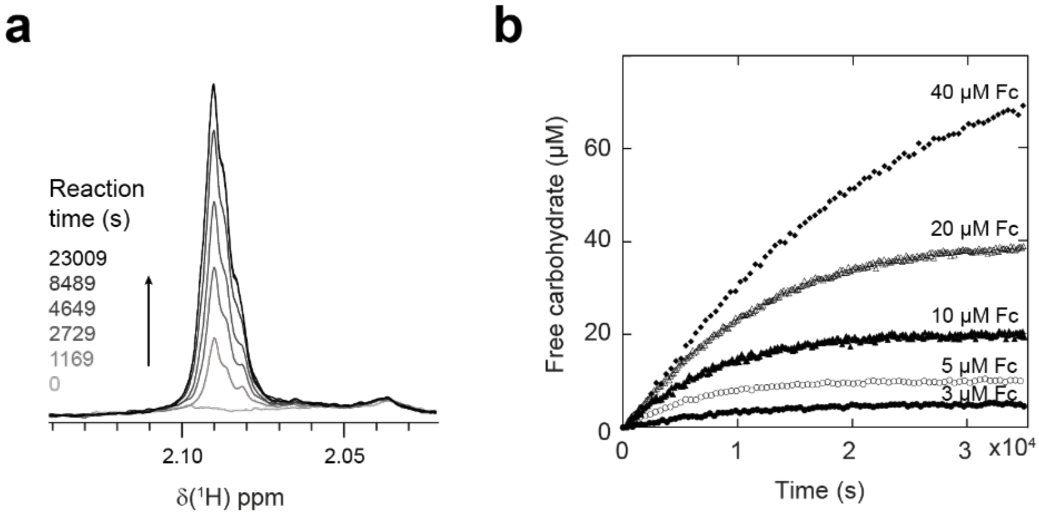


**Supplementary Figure 19 | Enzyme kinetics of EndoS2 and IgG1 Fc. a** The composite signal at 2.08-2.11 ppm was used to monitor the enzymatic cleavage of IgG1 Fc complex *N*-glycans as a function of time. Spectra corresponds to 40 µM Fc concentration. **b** Time courses of *N*-glycan cleaved from IgG1 Fc after the addition of EndoS2 at different IgG1 Fc starting concentrations. Note that two glycans are released per IgG1 Fc unit. Data at times longer than $3.5x10^4$ s were also included in the fit but are not shown for clarity. All experiments were acquired at 600 MHz and 298 K.

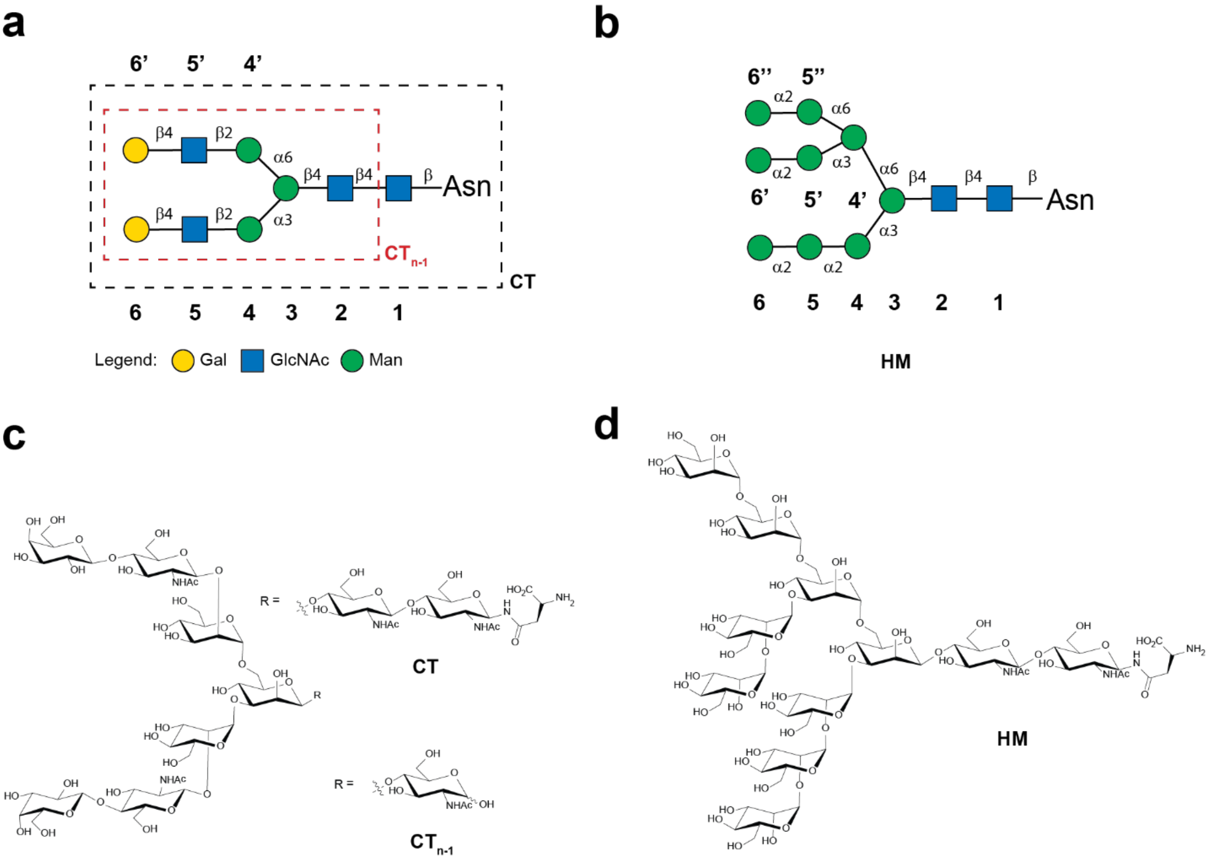


**Supplementary Figure 20 | Carbohydrates used for NMR titrations.** Cartoon representation of (**a**) complex-type *N*-glycan CT and its reaction product after treatment with EndoS2 $CT_{n-1}$, and of (**b**) high mannose-type *N*-glycan HM. Their corresponding chemical structures are shown in (**c**) for CT and $CT_{n-1}$, and in (**d**) for HM. Abbreviations in the legend correspond to: Gal = galactose, GalNAc = *N*-acetylgalactosamine, Man = mannose and Asn = asparagine.

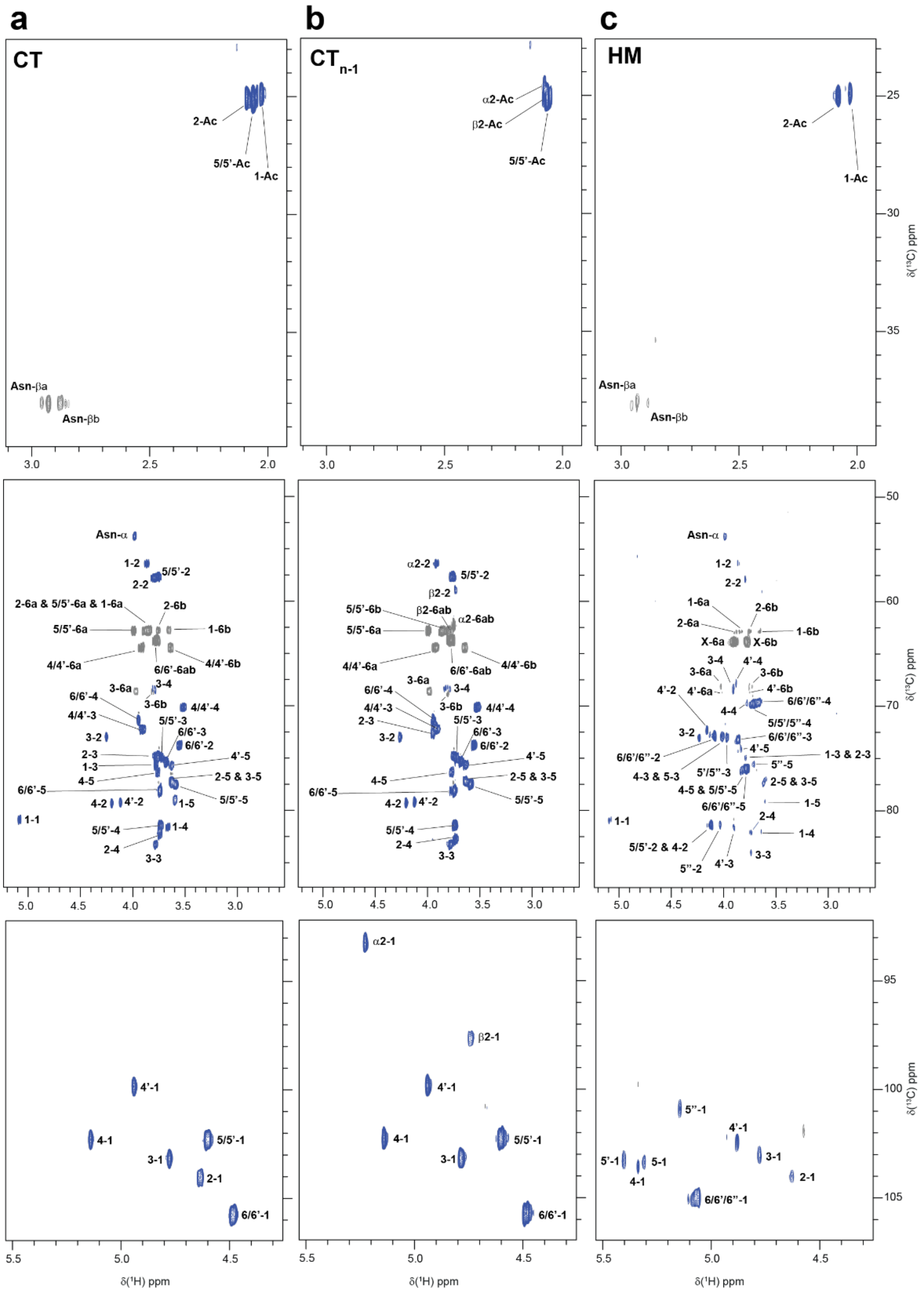


**Supplementary Figure 21 | $^{1}$H and $^{13}$C assignments of *N*-glycans used for NMR titrations.** Sections of $^{1}$H,$^{13}$C-HSQC spectra showing the assignment of (**a**) CT, (**b**) $CT_{n-1}$ and (**C**) HM. Spectra were acquired at 600 MHz and 310 K.

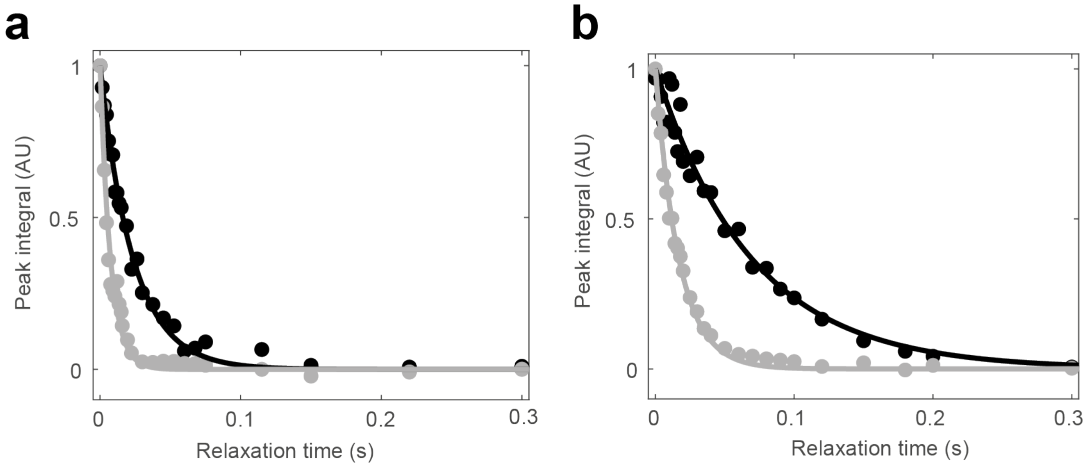


**Supplementary Figure 22 | $^{1}$H,$^{15}$N TRACT[14] was used to estimate protein rotational correlation times $\tau_c$, which indicates whether the protein sample is folded or unfolded in solution**. Shown are the to $^{15}$N $R_\alpha$ (black) and $R_\beta$ (grey) decay curves observed for (**a**) 71.5 µM of EndoS2$_{E186L}$ and (**b**) 74.4 µM of EndoBT$_{D312A/E314L}$ endoglycosidase mutants. Experiments were acquired at 600 MHz and 298 K.

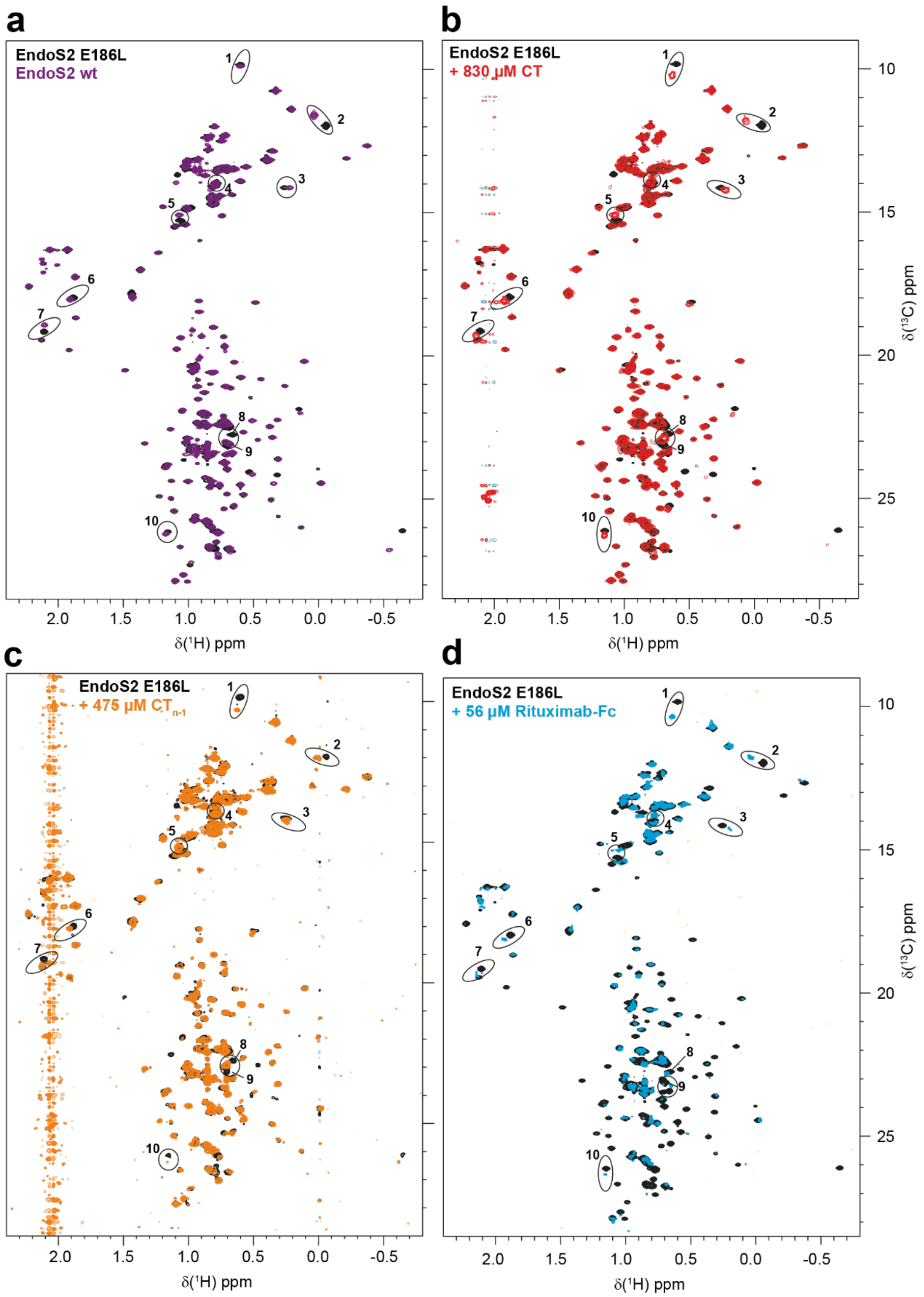


**Supplementary Figure 23 | CSPs observed for EndoS2 and EndoS2$_{E186L}$.** Superimposition of methyl-TROSY spectra corresponding to MILV-labeled (**a**) EndoS2$_{E186L}$ (black) and EndoS2 (violet), (**b**) EndoS2$_{E186L}$ in the absence (black) and in the presence of saturating concentrations of CT (red), (**c**) EndoS2$_{E186L}$ in the absence (black) and in the presence of close-to-saturation concentrations of CT$_{n-1}$ (orange) and (**d**) EndoS2$_{E186L}$ in the absence (black) and in the presence of saturating concentrations of Rituximab Fc (blue). Resonances highlighted in circles were used for lineshape analysis with TITAN. Spectra were acquired at 298 K and 600 MHz.

e

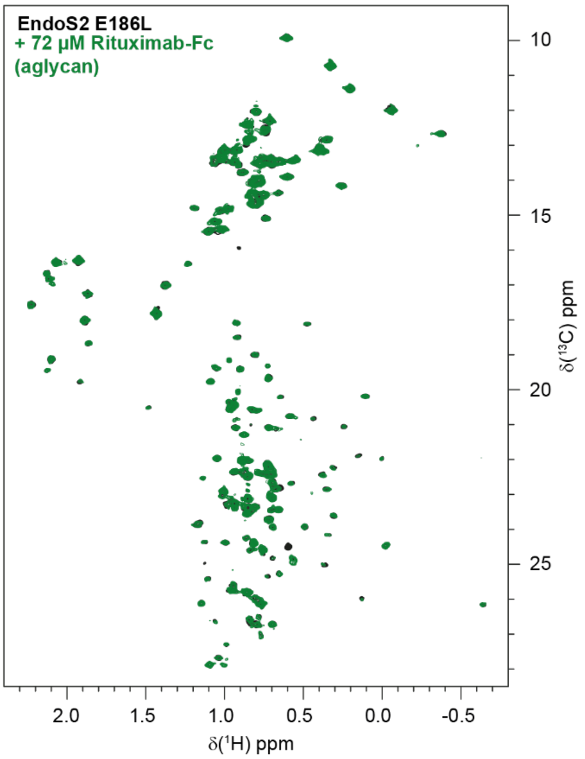


**Supplementary Figure 23 | continued. e** Superimposition of methyl-TROSY spectra corresponding to MILV-labeled EndoS2$_{E186L}$ in the absence (black) and in the presence of 72 μM of Rituximab-Fc aglycan (green). Spectra were acquired at 298 K and 600 MHz.

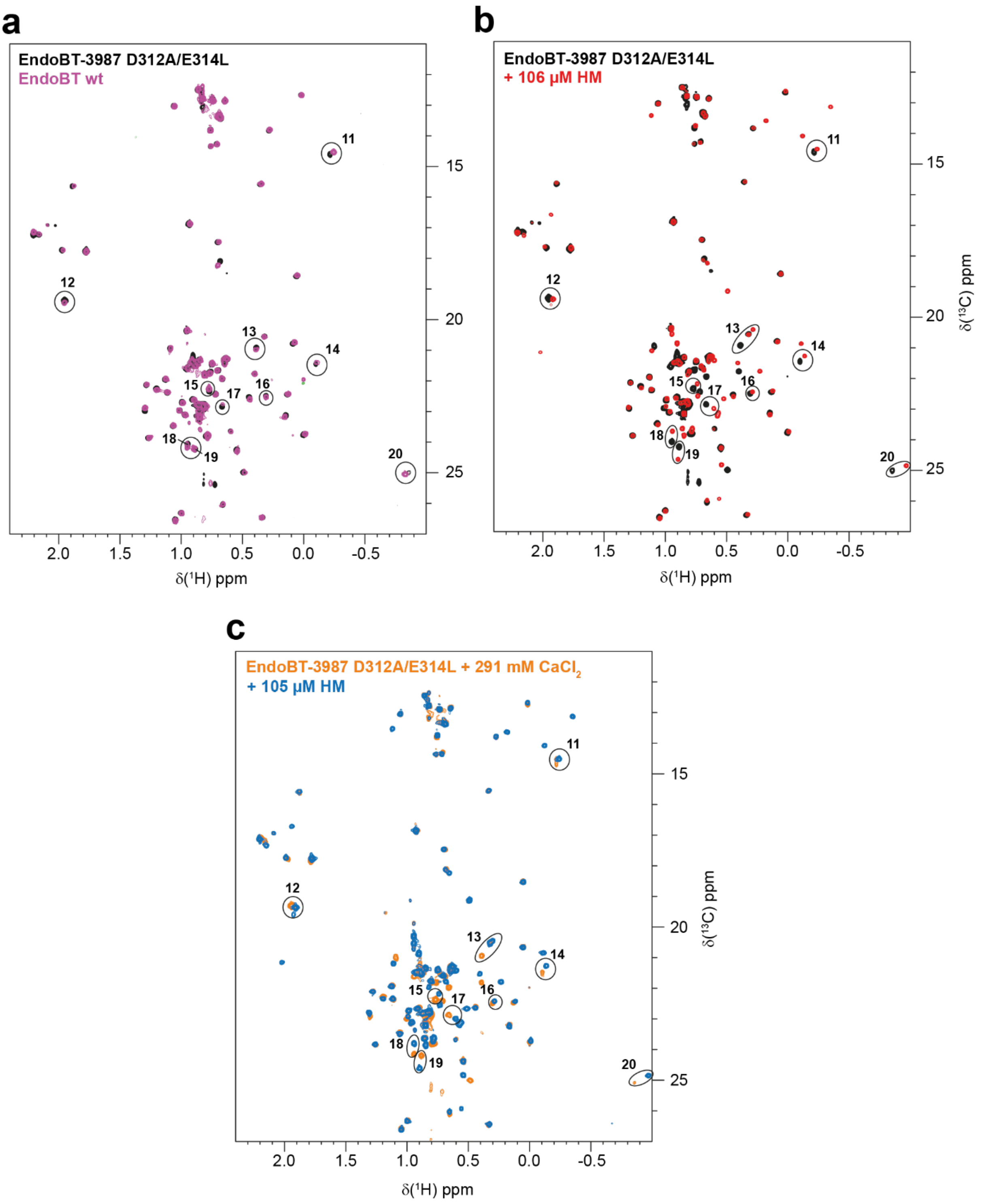


**Supplementary Figure 24 | CSPs observed for EndoBT-3987 and EndoBT-3987$_{D312A/E314}$.** **a** Superimposition of methyl-TROSY spectra of MILV-labeled EndoBT-3987 (purple) and EndoBT-3987$_{D312A/E314L}$ (black). **b** Superimposition of methyl-TROSY spectra of MILV-labeled and EndoBT-3987$_{D312A/E314L}$ in the absence (black) and in the presence (red) of saturating concentrations of HM. **c** Similar to (**b**) but under saturation concentrations of $CaCl_2$. Orange indicates no HM and blue saturation concentrations of HM. Resonances highlighted in circles were used for lineshape analysis with TITAN. Spectra were acquired at 298 K and 600 MHz.

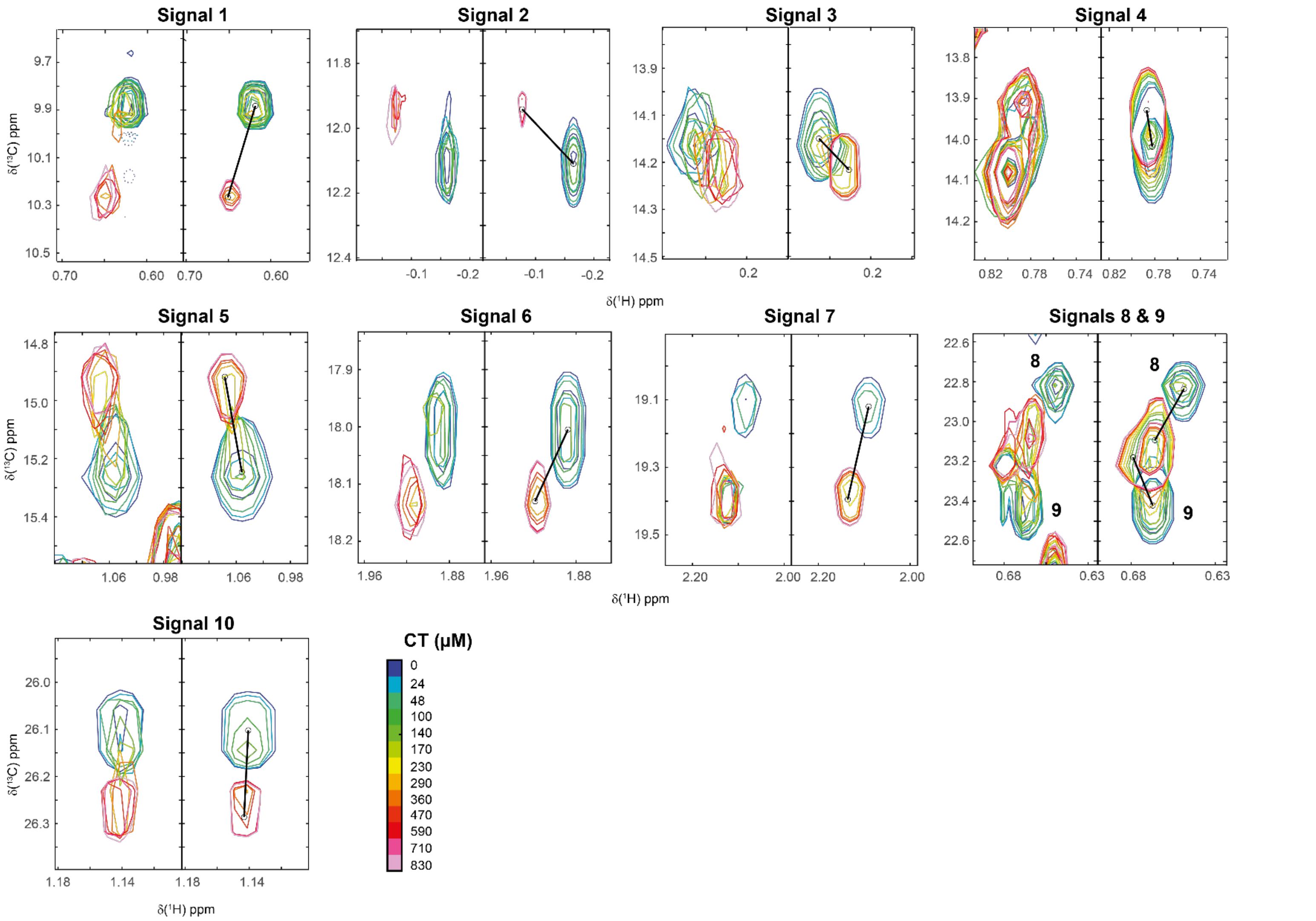


**Supplementary Figure 25 | Complete set of cross peaks used for the TITAN analysis of CT binding to MILV-labeled EndoS2$_{\mathbf{E186L}}$.** The left panel shows the sections from the methyl-TROSY experiment as measured. The right panel shows the simulated cross peaks using a simple two-states binding model.

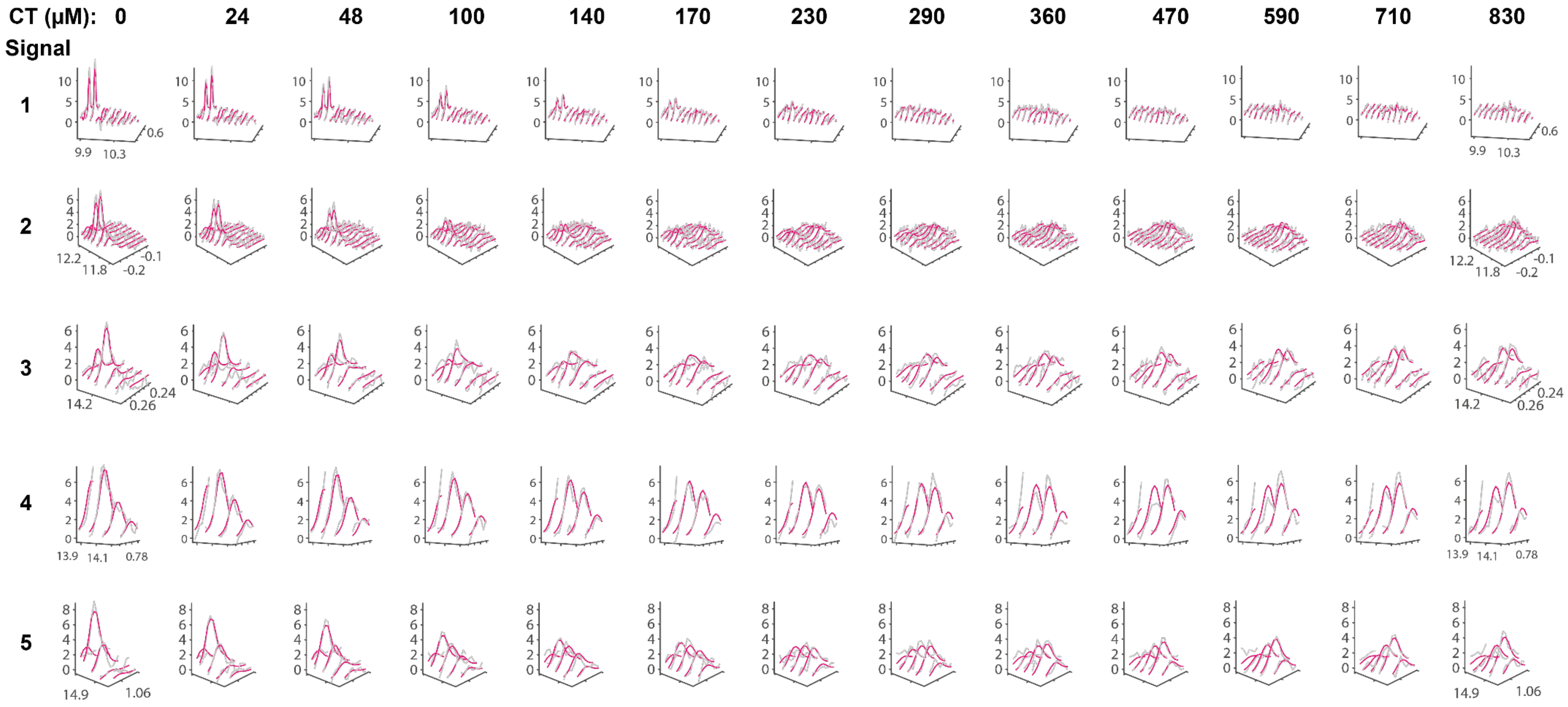


**Supplementary Figure 26 | 2D lineshape analysis of methyl-TROSY spectra of a MILV-labeled sample of EndoS2$_{E186L}$ at increasing CT concentrations.** Global lineshape analysis of a series of concentration dependent methyl-TROSY spectra using TITAN[13] yielded a fitted set of 10 signals. It should be noted that in principle this analysis does not depend on any assignments. Experimental and fitted cross peak are shown in grey and magenta, respectively, reflecting the good quality of the fit. TITAN analysis results in a dissociation constant $K_D$ of 106.9 ± 6.7 µM and a dissociation rate constant $k_{off}$ of 11.4 ± 1.5 $s^{-1}$.

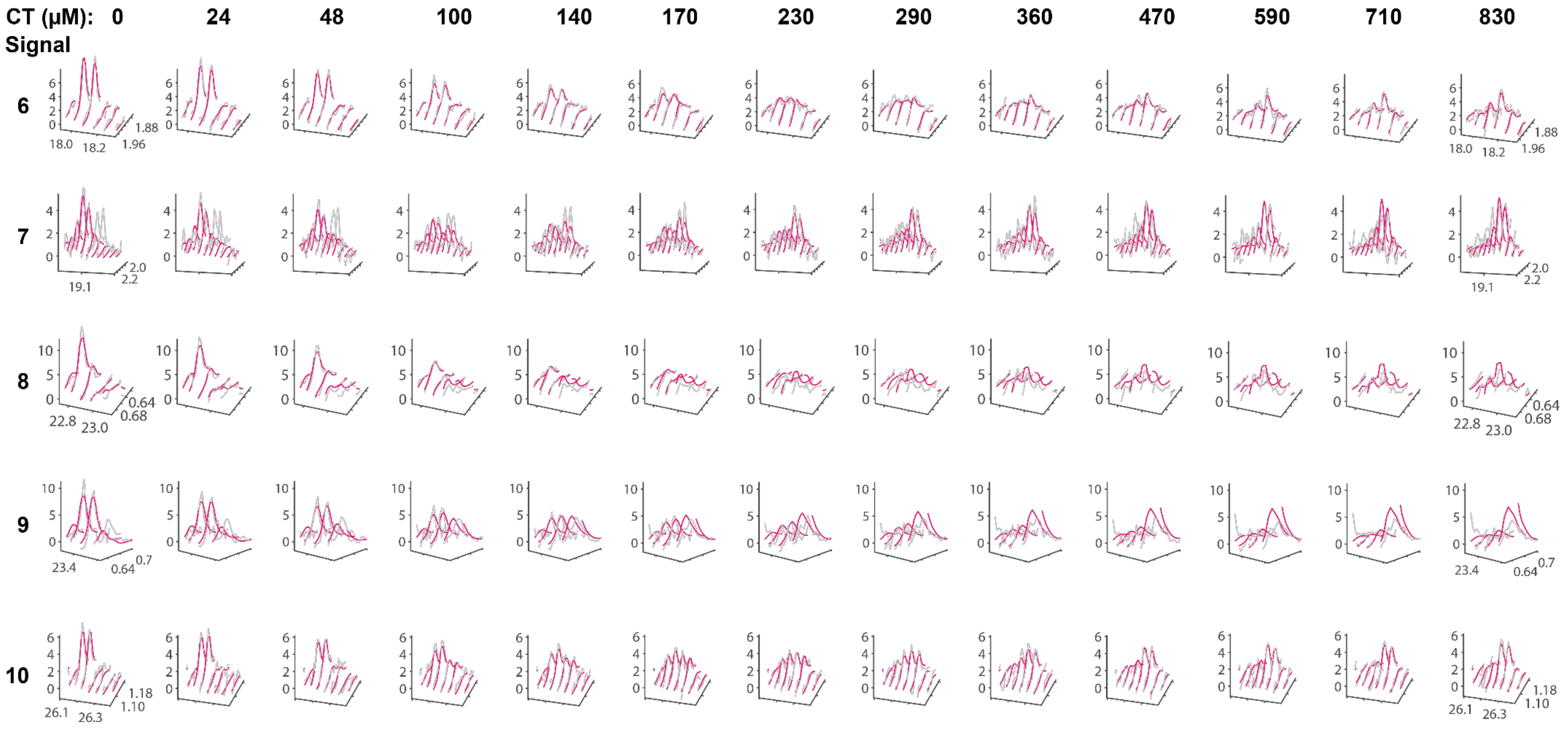


**Supplementary Figure 26 | continued.**

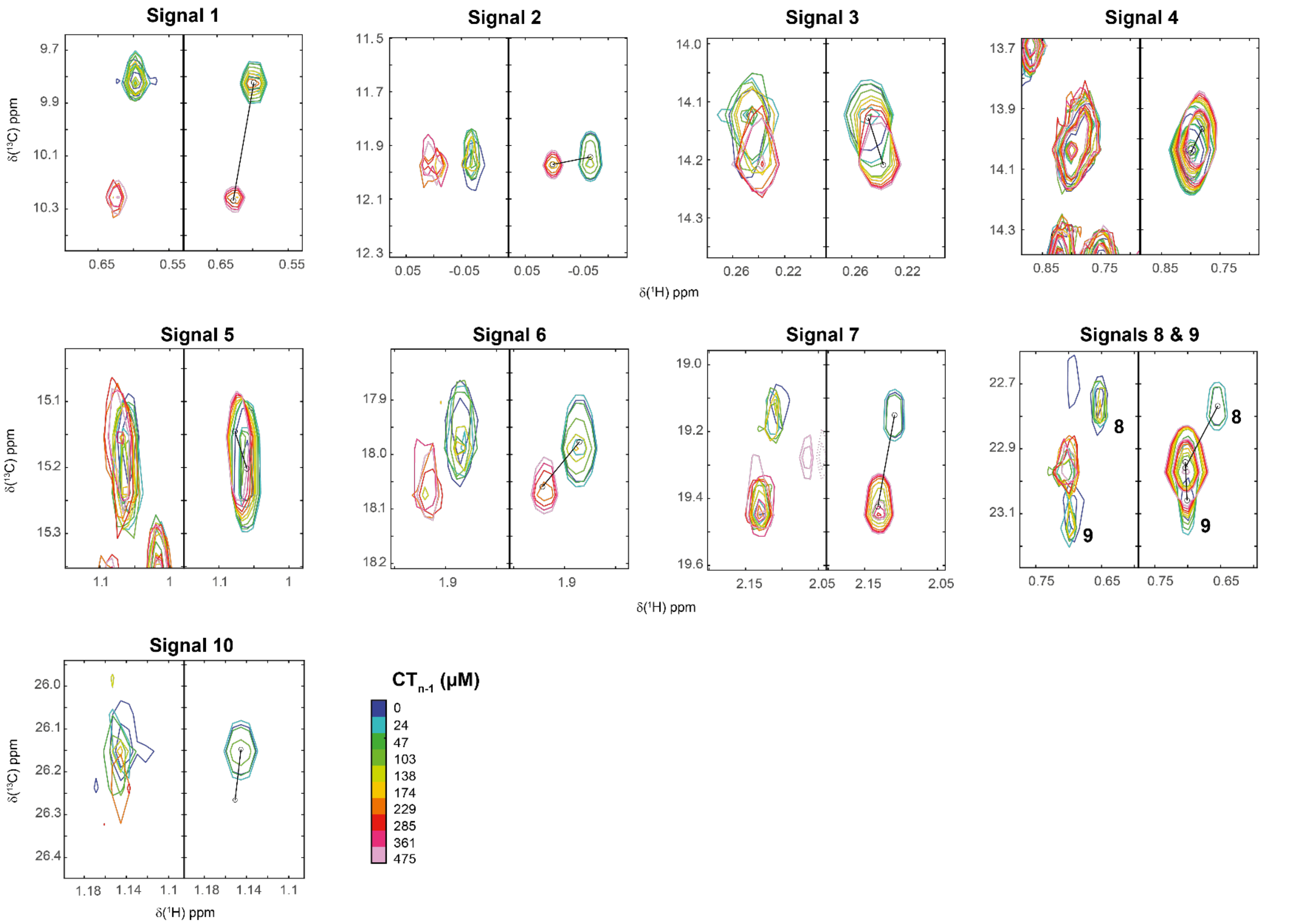


**Supplementary Figure 27 | Complete set of cross peaks used for the TITAN analysis of $CT_{n-1}$ binding to MILV-labeled $EndoS2_{E186L}$.** The left panel shows the sections from the methyl-TROSY experiment as measured. The right panel shows the simulated cross peaks using a simple two-states binding model.

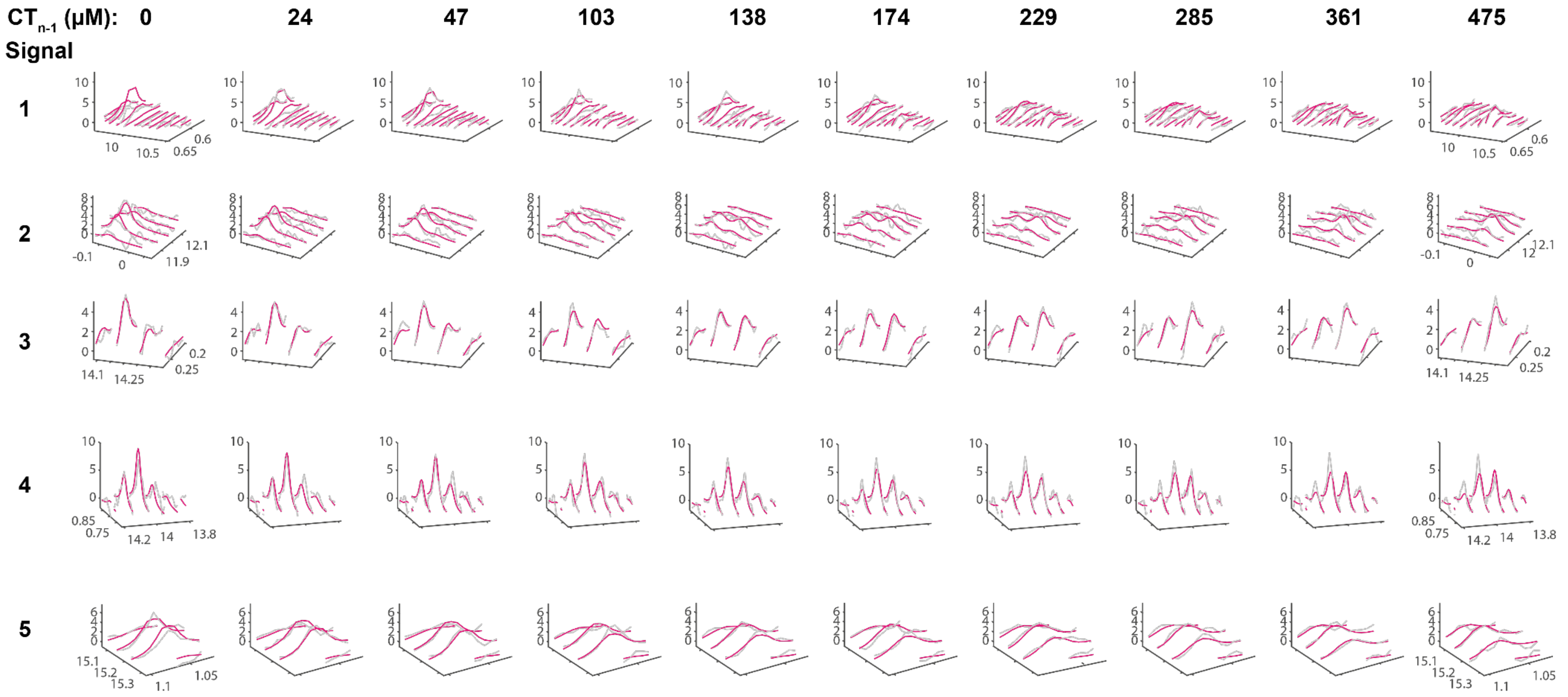


**Supplementary Figure 28 | 2D lineshape analysis of methyl-TROSY spectra of a MILV-labeled sample of EndoS2$_{E186L}$ at increasing CT$_{n-1}$ concentrations.** Global lineshape analysis of a series of concentration dependent methyl-TROSY spectra using TITAN[13] yielded a fitted set of 10 signals. It should be noted that in principle this analysis does not depend on any assignments. Experimental and fitted cross peak are shown in grey and magenta, respectively, reflecting the good quality of the fit. TITAN analysis results in a dissociation constant $K_D$ of 150.7 ± 18.0 μM and a dissociation rate constant $k_{off}$ of 9.4 ± 3.7 s$^{-1}$.

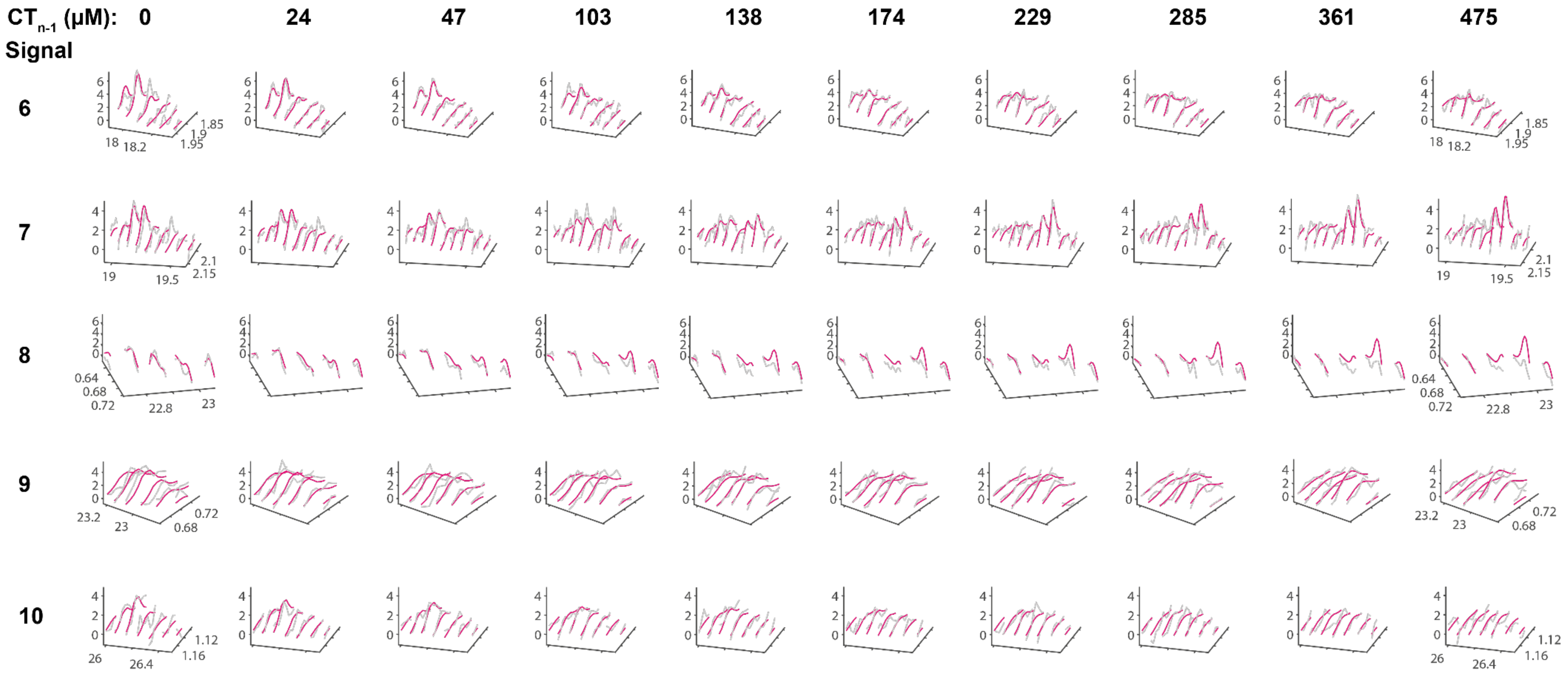


**Supplementary Figure 28 | continued.**

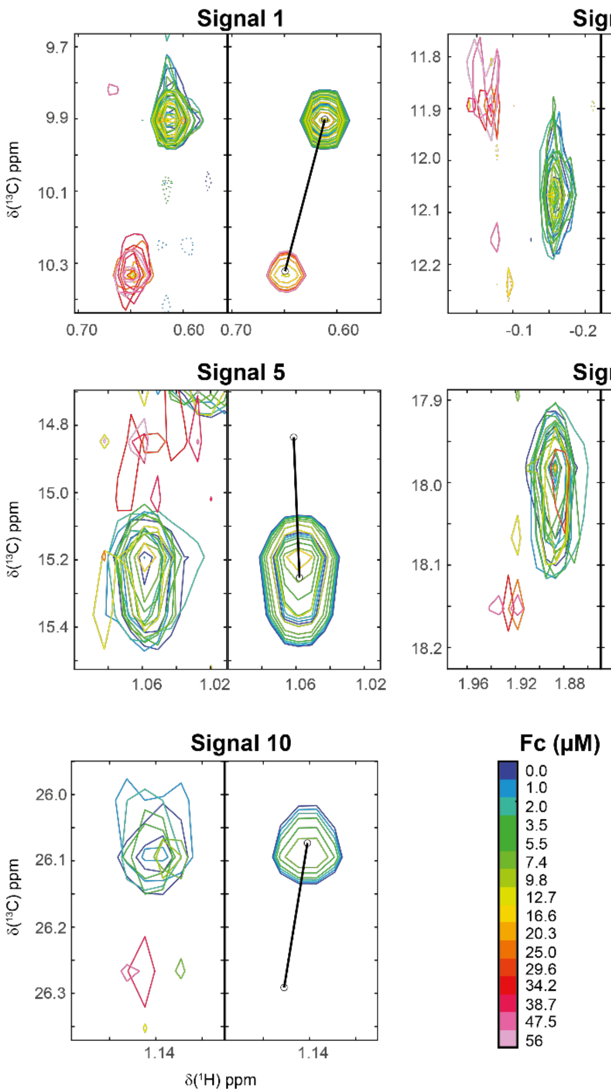


**Supplementary Figure 29 | Complete set of cross peaks used for the TITAN analysis of Rituximab-Fc binding to MILV-labeled EndoS2$_{\mathbf{E186L}}$.** The left panel shows the sections from the methyl-TROSY experiment as measured. The right panel shows the simulated cross peaks using a simple two-states binding model.

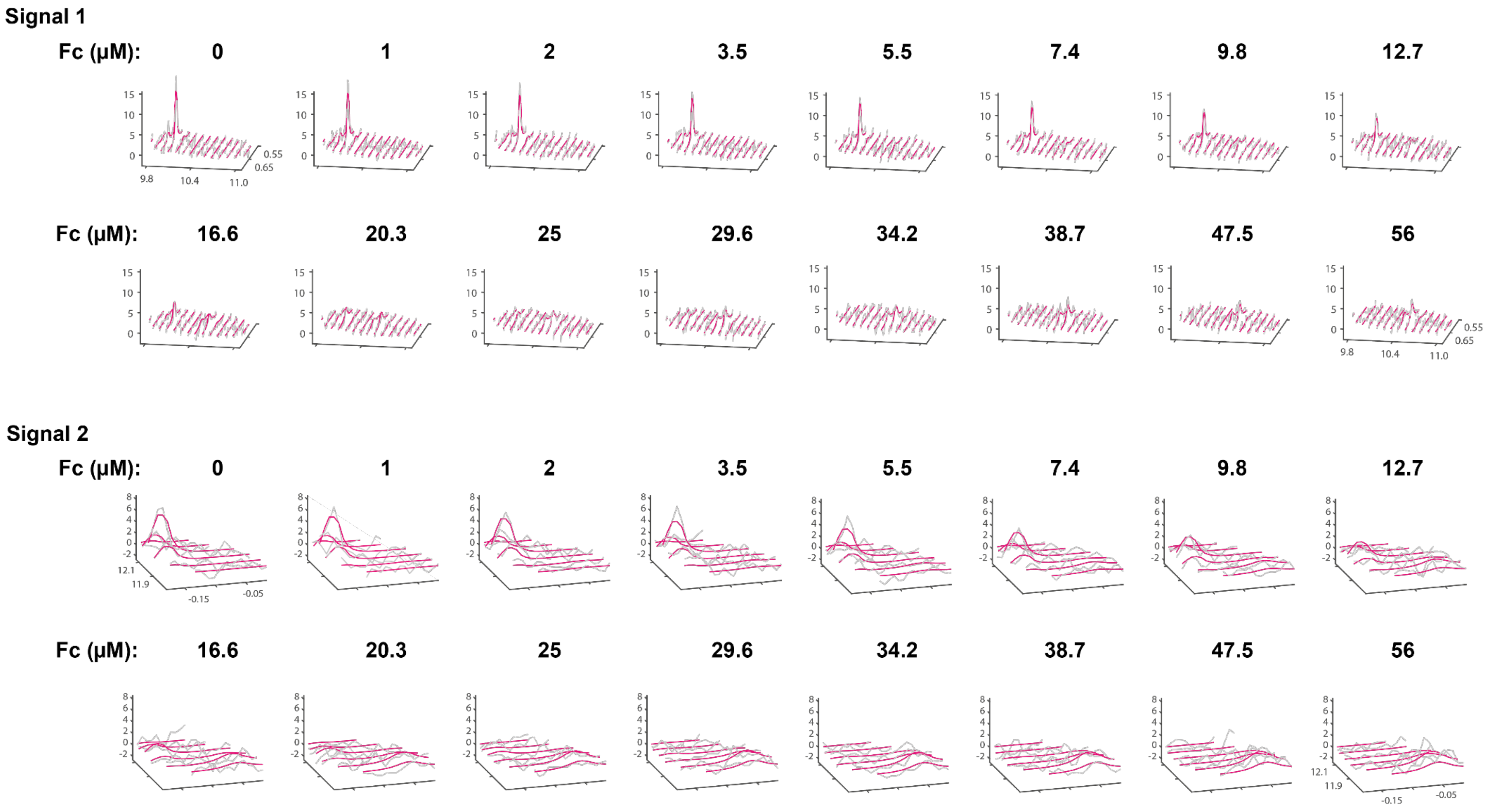


**Supplementary Figure 30 | 2D lineshape analysis of methyl-TROSY spectra of a MILV-labeled sample of EndoS2$_{E186L}$ at increasing Rituximab-Fc concentrations.** Global lineshape analysis of a series of concentration dependent methyl-TROSY spectra using TITAN[13] yielded a fitted set of 10 signals. It should be noted that in principle this analysis does not depend on any assignments. Experimental and fitted cross peak are shown in grey and magenta, respectively, reflecting the good quality of the fit. TITAN analysis results in a dissociation constant $K_D$ of 3.1 ± 0.6 µM and a dissociation rate constant $k_{off}$ of 4.0 ± 1.5 s$^{-1}$.

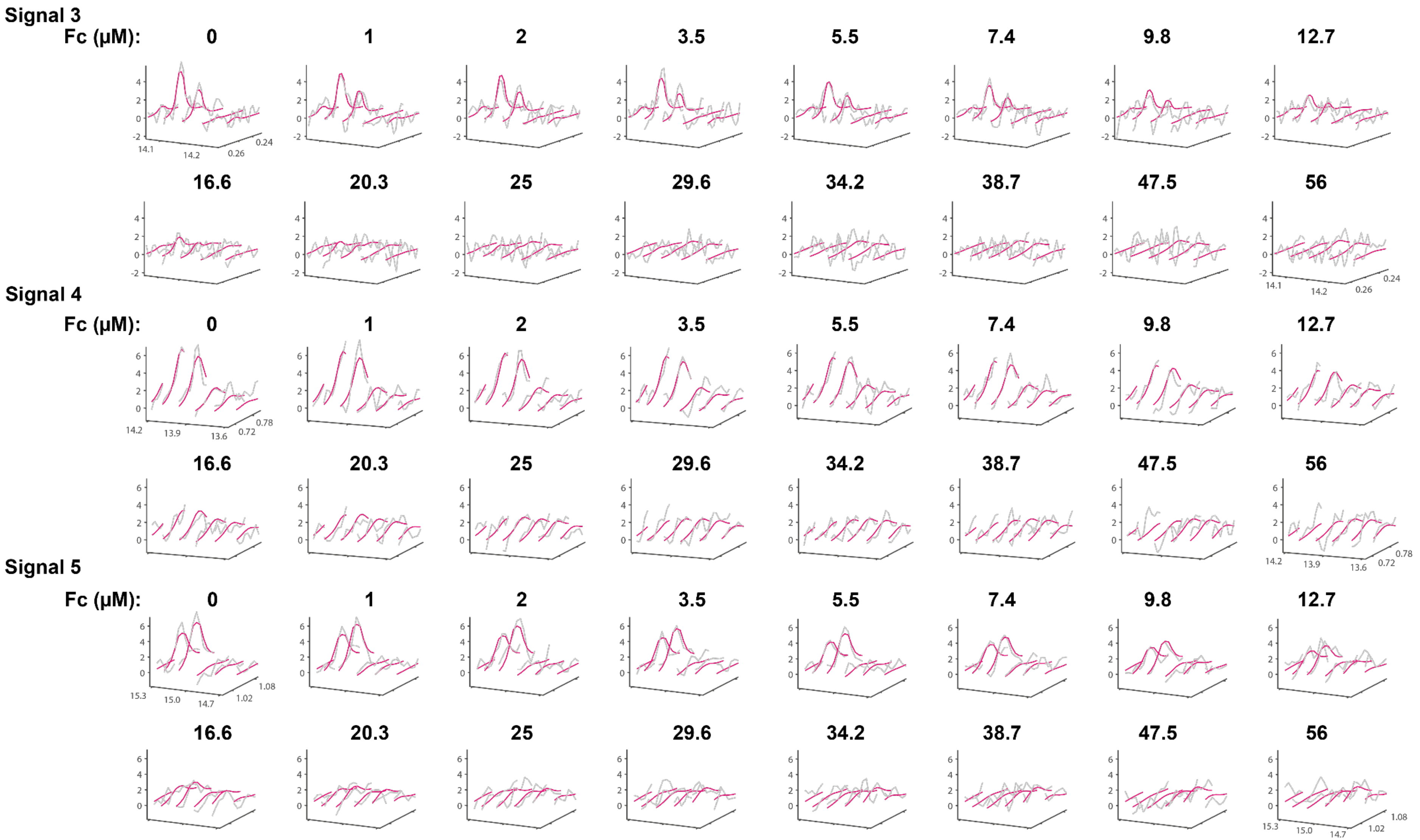


**Supplementary Figure 30 | continued.**

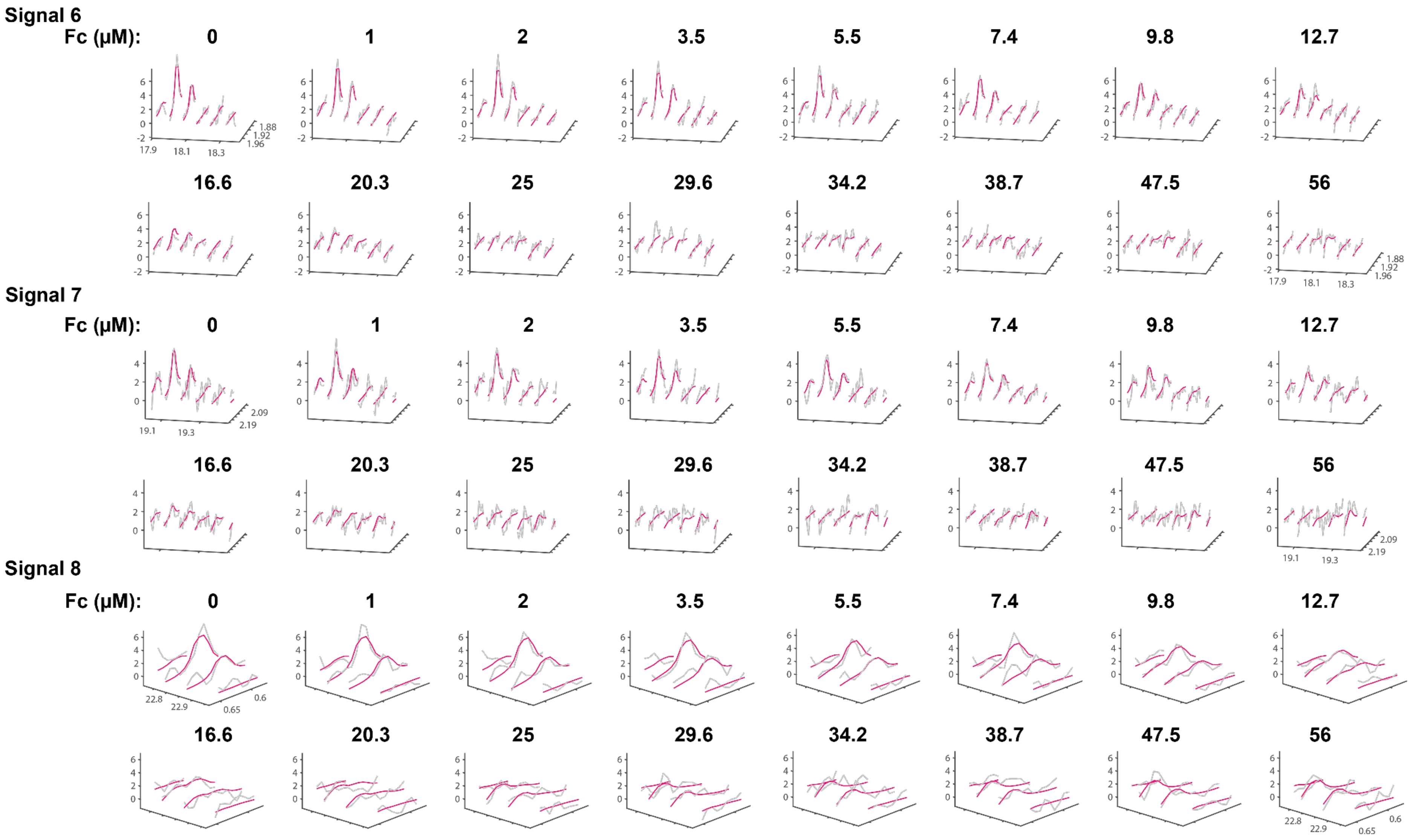


**Supplementary Figure 30 | continued.**

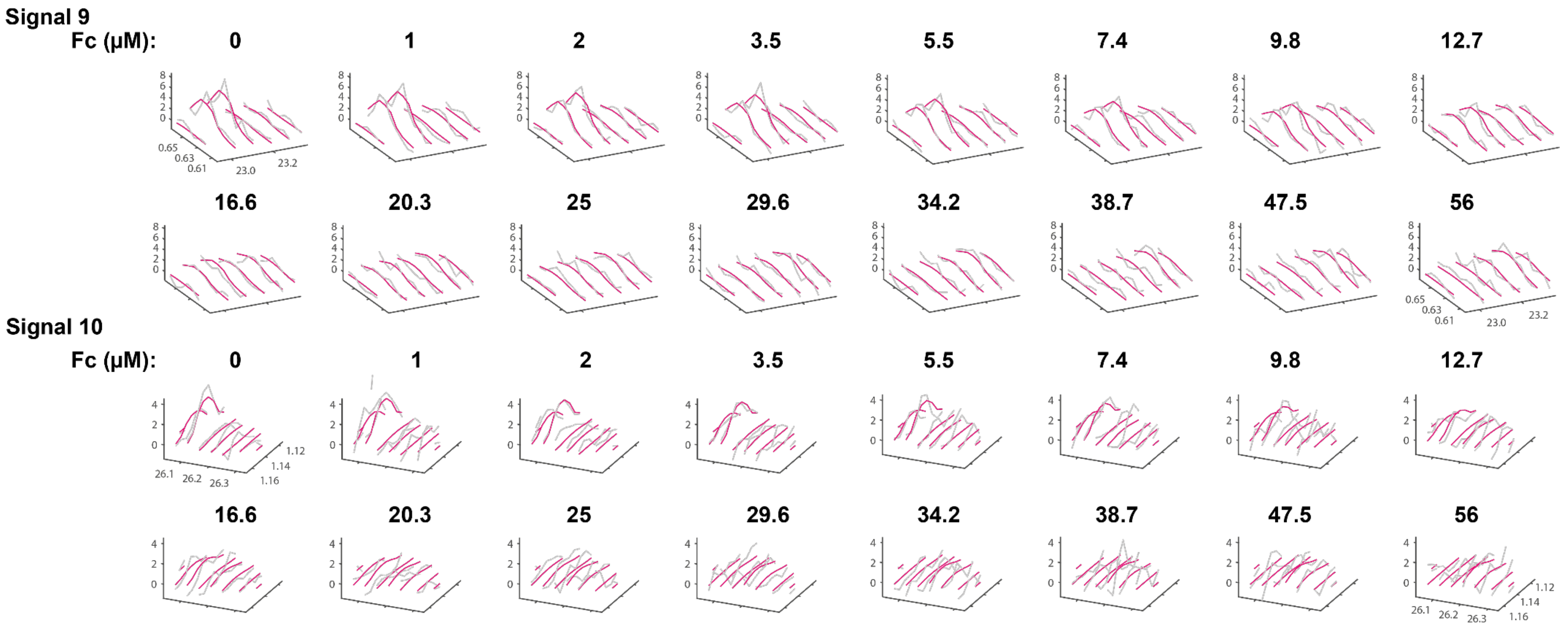


**Supplementary Figure 30 | continued.**

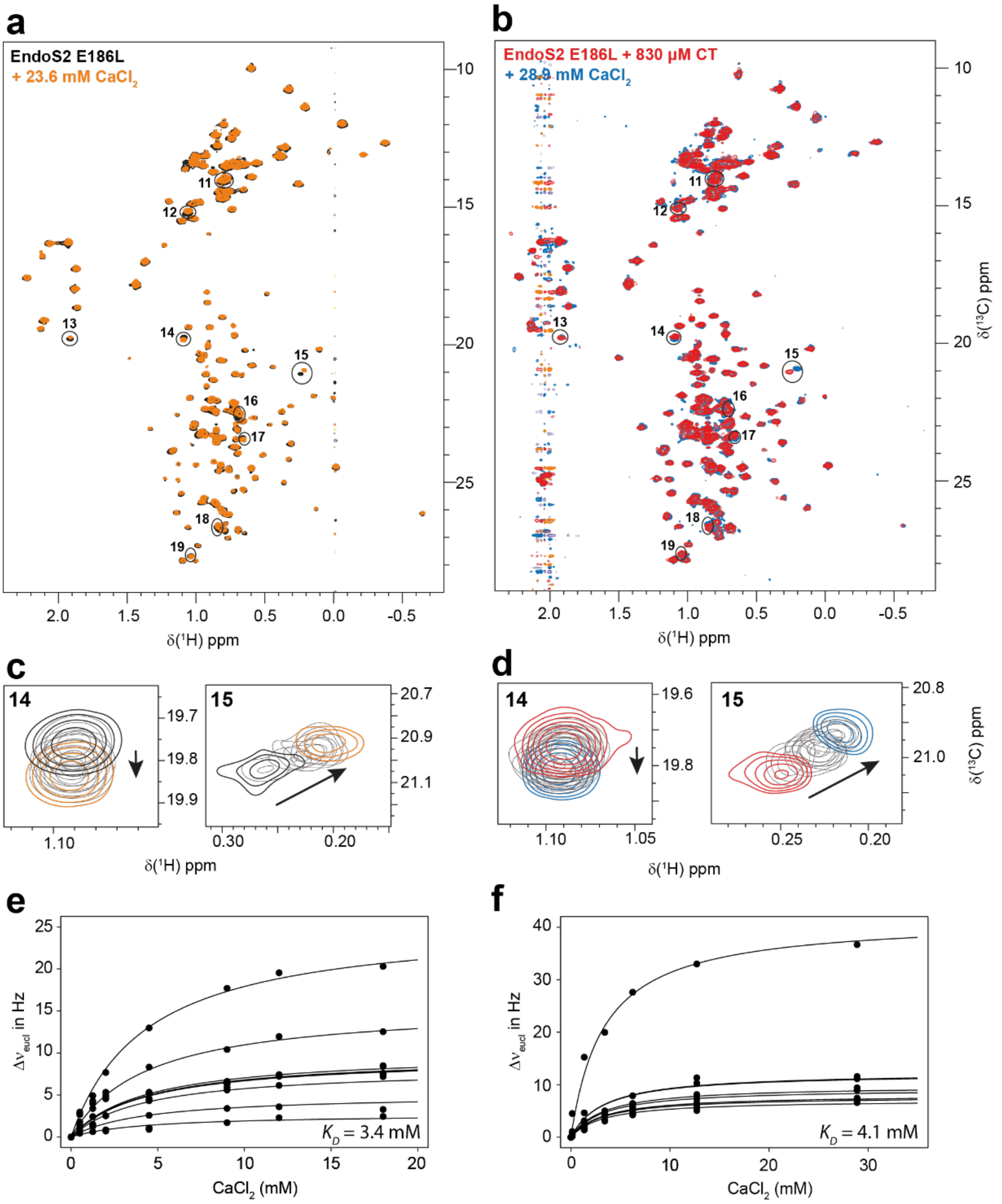


**Supplementary Figure 31 | Titration of EndoS2 and EndoS2$_{E186L}$ with CaCl$_2$.** Superimposition of methyl-TROSY spectra corresponding to the titration of MILV-labeled EndoS2$_{E186L}$ with CaCl$_2$ (**a**) in the absence and (**b**) in the presence of saturating concentrations of CT. Spectra acquired in the absence and in the presence of saturating concentrations of Ca$^{2+}$ are colored in (**a**) black and orange in, and (**b**) in red and blue, respectively. Resonances highlighted in circles were used to extract dissociation constants from CSPs. Exchange between calcium-free and calcium-bound protein forms are in fast exchange in the NMR time-scale, as can be seen for selected resonances in (**c**) the absence and (**d**) in the presence of saturating concentrations of CT. Dissociation constants $K_D$ were calculated from the fitting of CSPs to the law of mass action. Binding isotherms corresponding to the titration of calcium (**e**) in the absence and (**f**) under saturation concentrations of CT. Spectra were acquired at 298 K and 600 MHz.

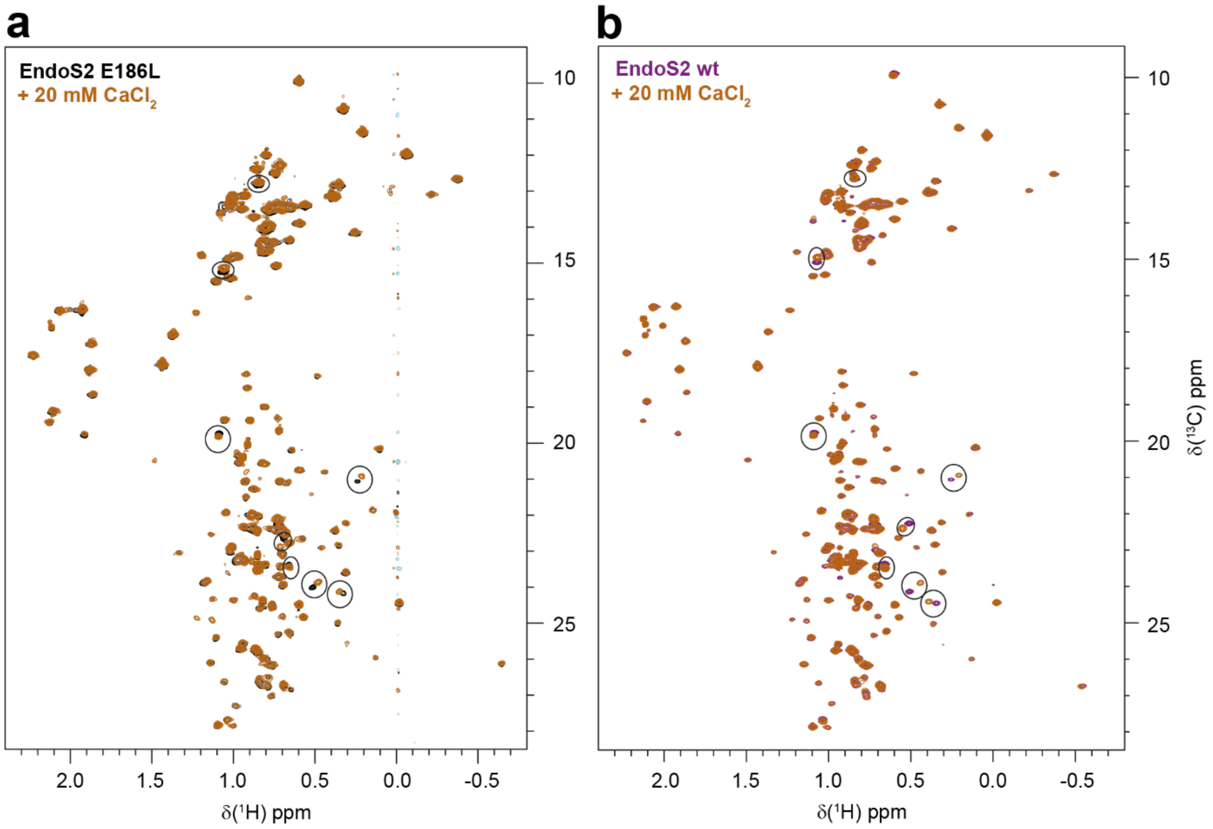


**Supplementary Figure 32 | Similar CSPs were observed for the binding of $Ca^{2+}$ to EndoS2$_{E186L}$ and to EndoS2.** Superimposition of methyl-TROSY spectra corresponding to MILV-labeled (**a**) EndoS2$_{E186L}$ in the absence (black) and in the presence of saturating concentrations of $CaCl_2$ (yellow), (**b**) and of EndoS2 in the absence (violet) and in the presence of saturating concentrations of $CaCl_2$ (yellow). Resonances highlighted in circles shift upon titration with $CaCl_2$. Spectra were acquired at 298 K and 600 MHz.

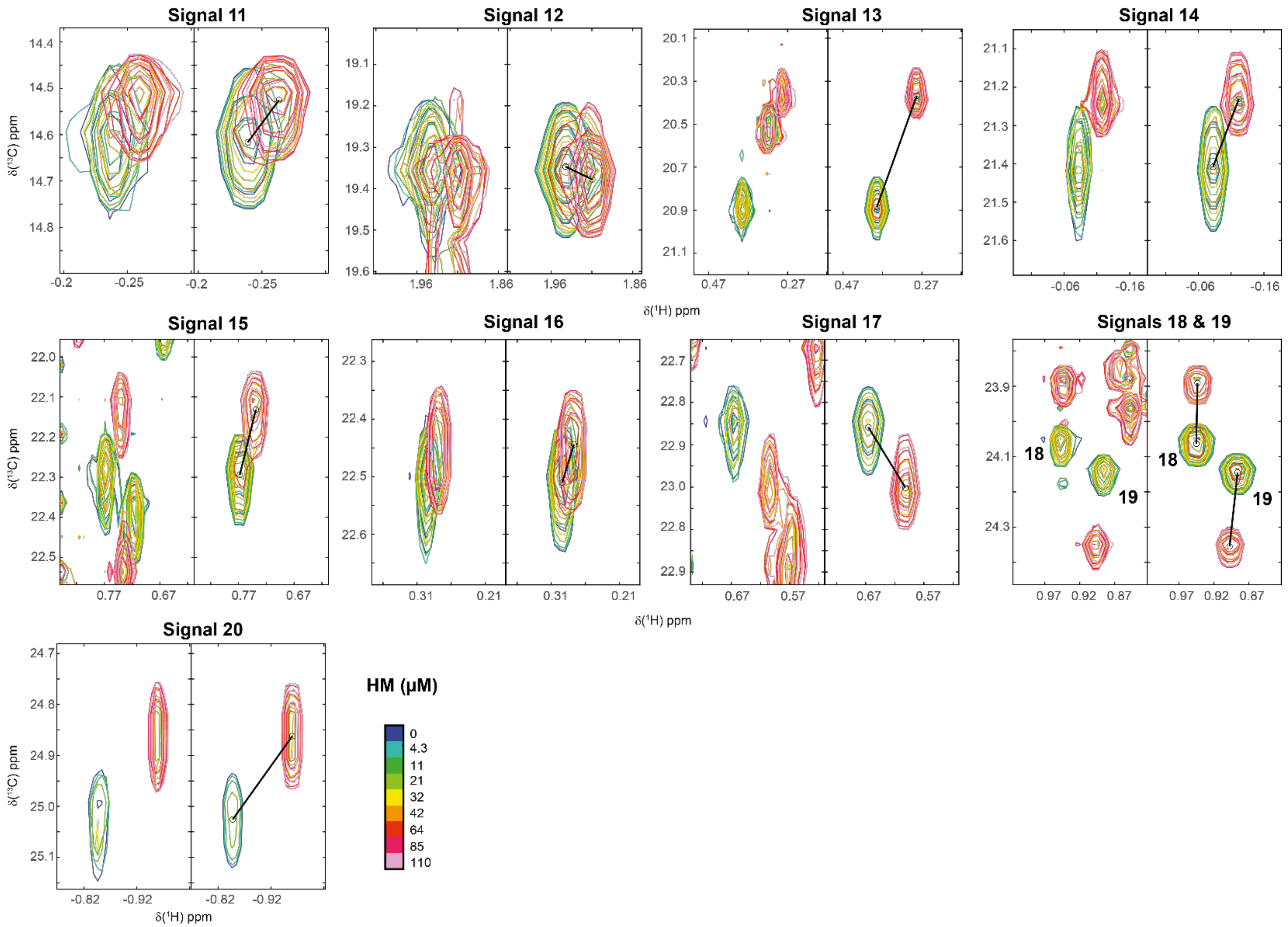


**Supplementary Figure 33 | Complete set of cross peaks used for the TITAN analysis of HM binding to MILV-labeled EndoBT$_{D312A/E314L}$ in the absence of $CaCl_2$.** The left panel shows the sections from the methyl-TROSY experiment as measured. The right panel shows the simulated cross peaks using a simple two-states binding model.

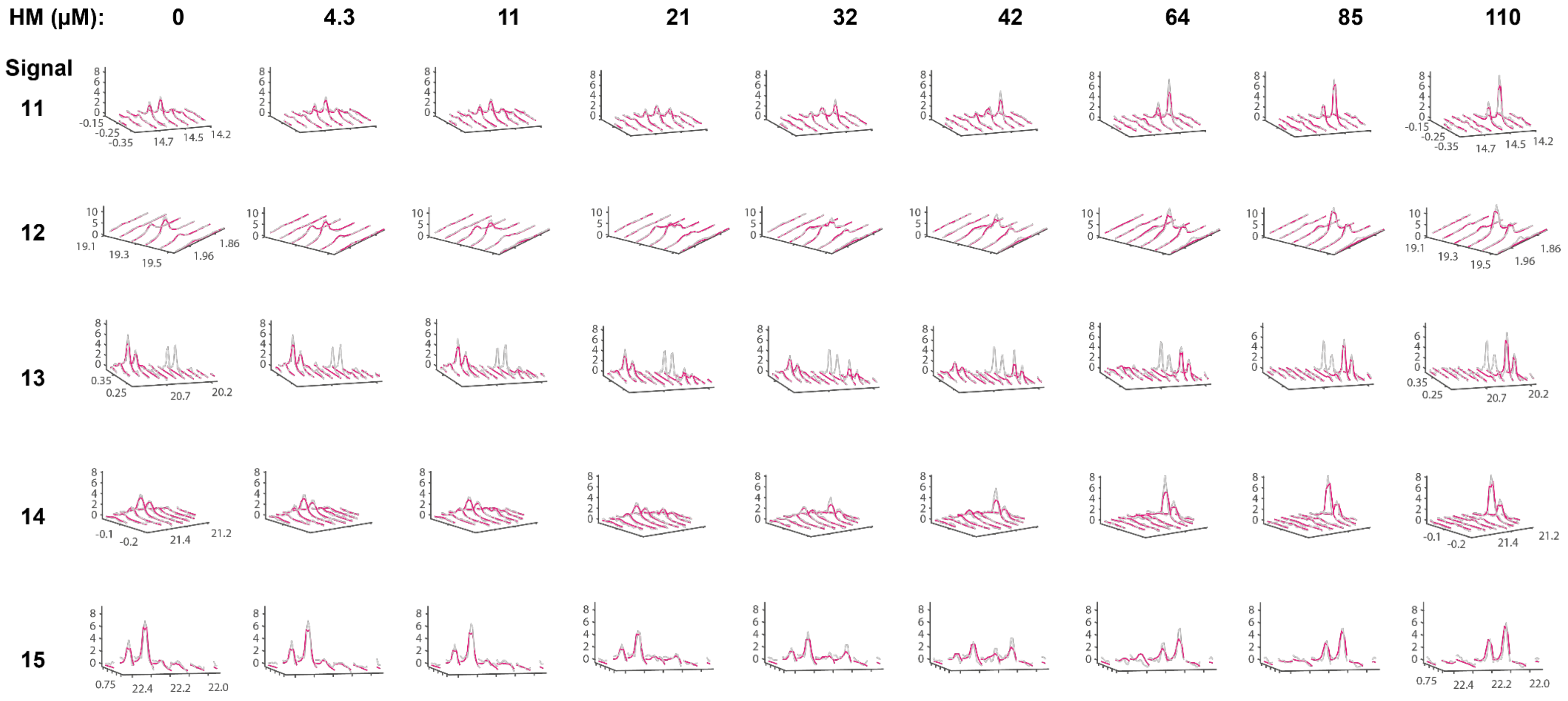


**Supplementary Figure 34 | 2D lineshape analysis of methyl-TROSY spectra of a MILV-labeled sample of EndoBT$_{D312A/E314L}$ at increasing HM concentrations in the absence of $CaCl_2$.** Global lineshape analysis of a series of concentration dependent methyl TROSY spectra using TITAN[13] yielded a fitted set of 10 signals. It should be noted that in principle this analysis does not depend on any assignments. Experimental and fitted cross peak are shown in grey and magenta, respectively, reflecting the good quality of the fit. TITAN analysis results in a dissociation constant $K_D$ of 1.7 ± 0.9 μM and a dissociation rate constant $k_{off}$ of 5.5 ± 1.4 $s^{-1}$.

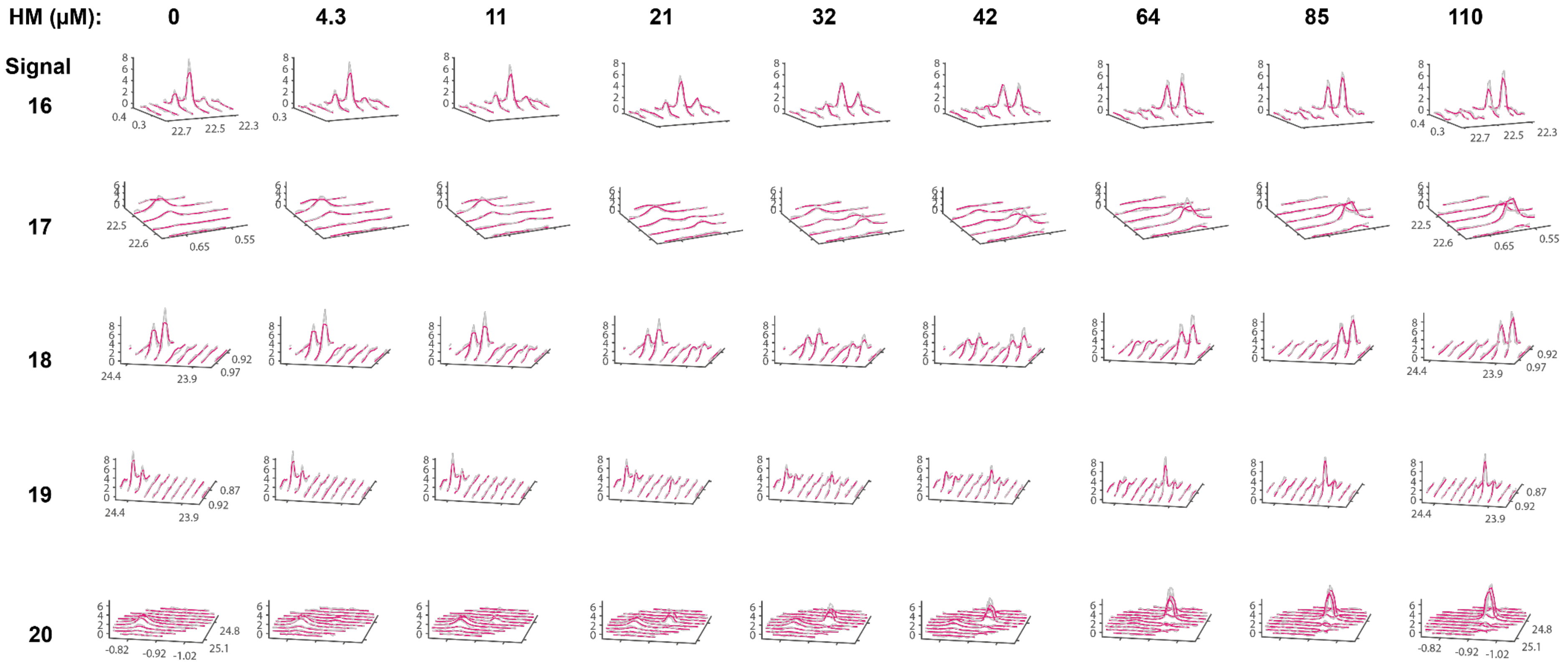


**Supplementary Figure 34 | continued.**

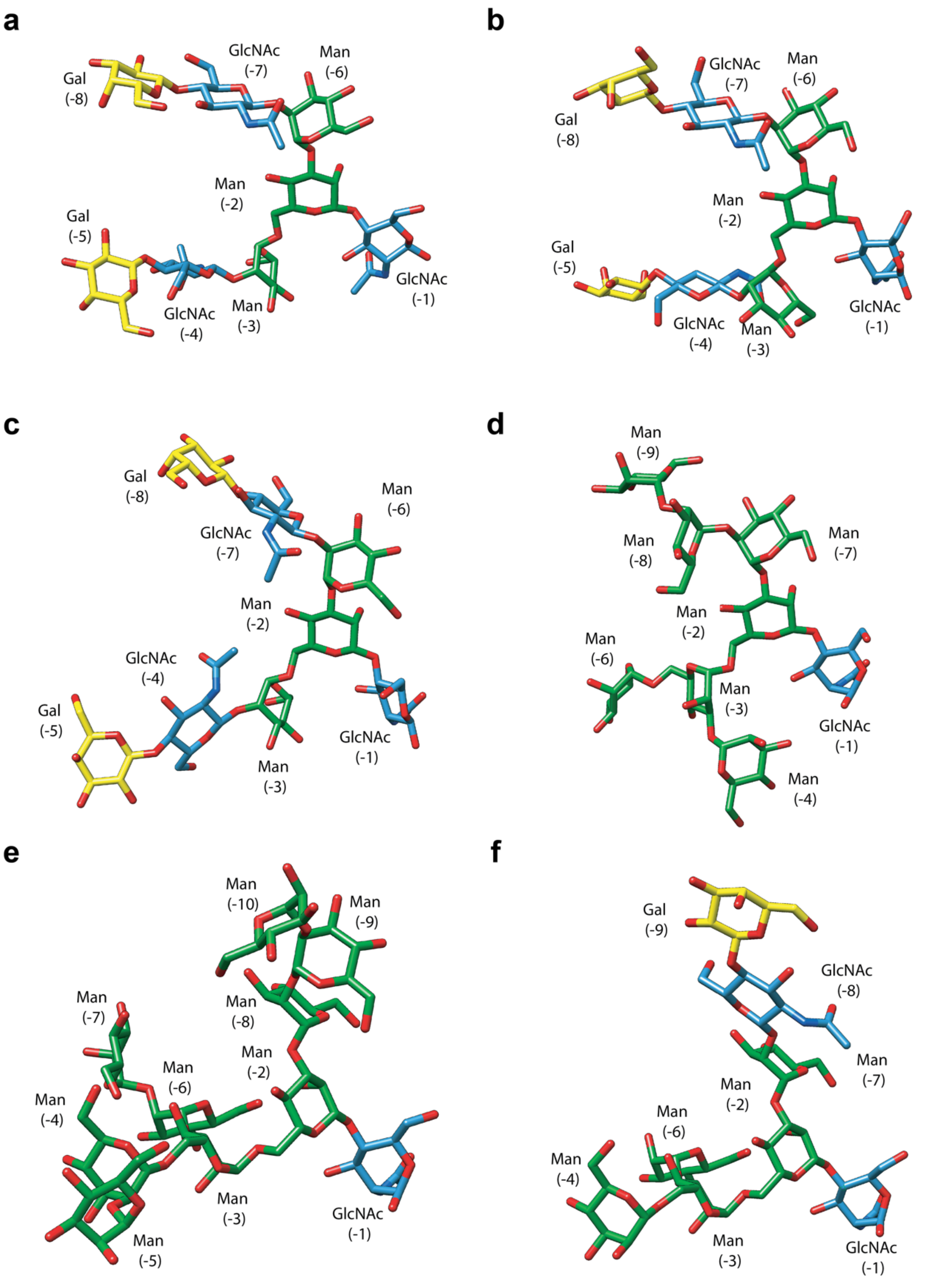


**Supplementary Fig. 35 | *N*-glycan conformations in the active site of ENGases.** **a** Distinct conformation of *N*-glycans found in the crystal structures of EndoS-CT (PDB code 6EN3), **b** EndoS2-CT (PDB code 6MDS), **c** EndoF3 (PDB code 1EOM, **d** EndoS2-HM (PDB code 6MDV), **e** EndoBT-3987-HM (PDB code 6T8L), **f** EndoBT-3987-Hy (PDB code 7NWF).

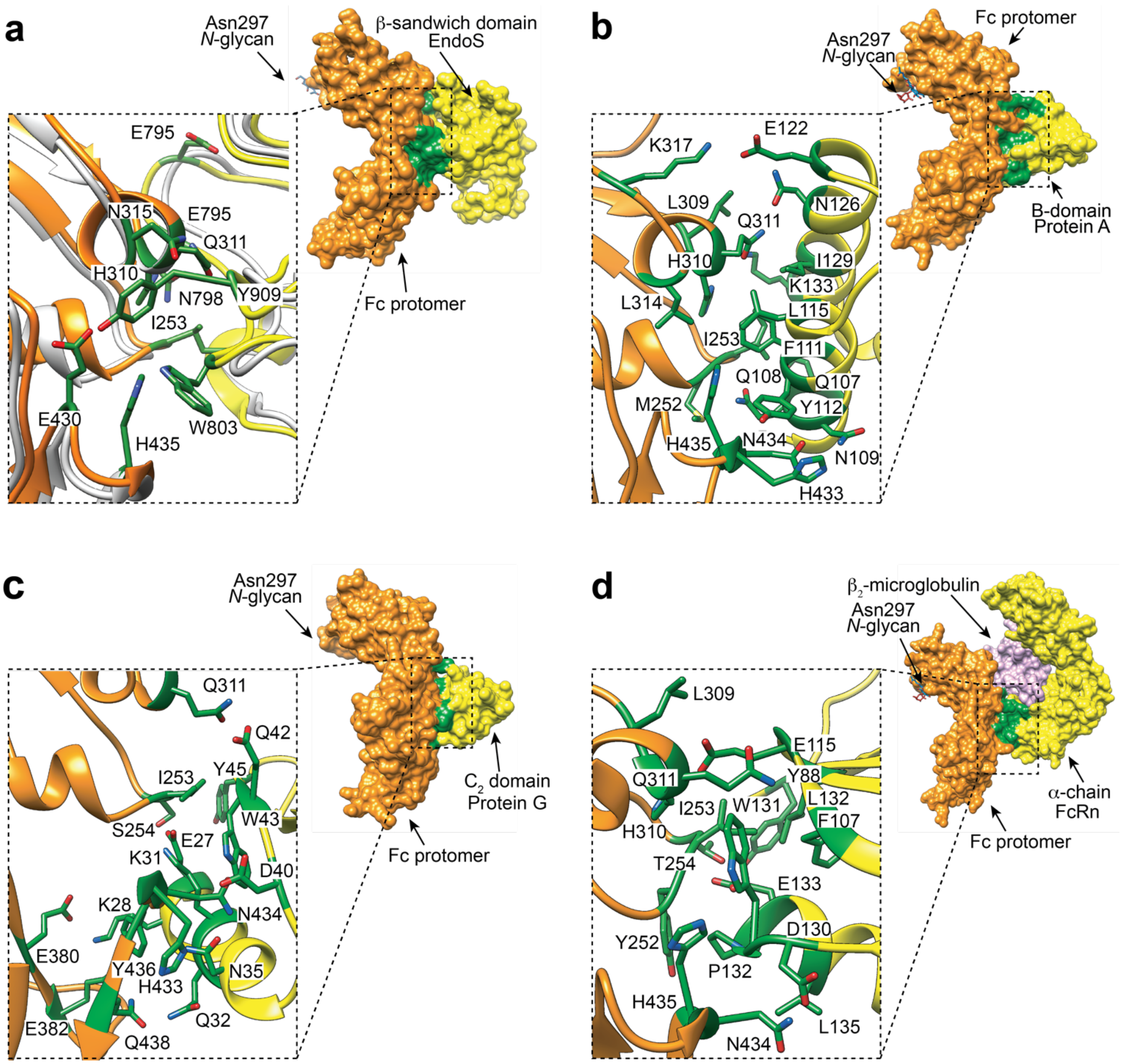


**Supplementary Fig. 36 | Structural comparison of the interaction interface of EndoS, Protein A, Protein G and FcRn and the Fc region of IgG1. a** cryoEM model of EndoS$_{E235A}$ in complex with IgG1 Fc (PDB code 8A64) colored in grey. In order to have insights on the possible location of the side chain residues that could be involved in the enzyme-substrate interactions, we locally compared/superimposed the high-resolution EndoS (PDB code 6EN3; yellow) and Fc region (PDB code 2DTS; orange) crystal structures. Therefore, we visualize potential side chain interactions between residues that are within interaction distance based on flexible fitting of the superimposed crystal structures. **b** X-ray crystal structure of B-domain from Protein A in complex with IgG1 Fc IgG1(PDB code 5UAY). **c** X-ray crystal structure of the C2 fragment of Protein G in complex with IgG1 Fc (PDB code 1FCC) **d** X-ray crystal structure of the ternary complex between FcRn, serum albumin and IgG1 Fc-YTE (PDB code 4N0U).

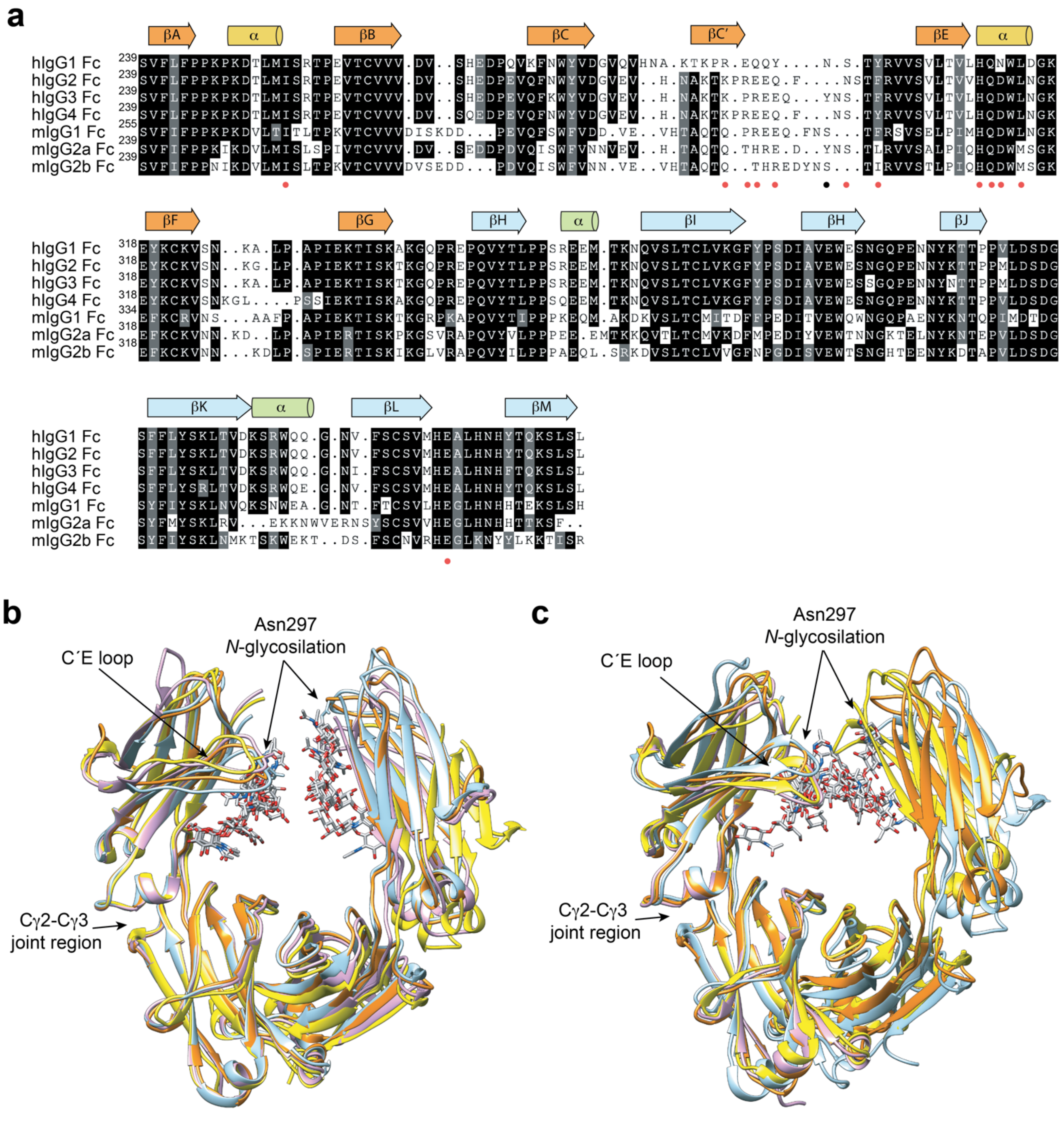


**Supplementary Fig. 37 | Structural comparison of human (hIgG1-4) and murine (mIgG1, mIgG2a and mIgG2b) IgG subclasses. a** Structure weighted sequence alignment of the human IgG1-4 and murine IgG1, IgG2a and IgG2b subclasses. Comparison of hIgG1 Fc[18] (Uniprot code, P01857; PDB code, 1H3X), hIgG2 Fc[19] (Uniprot code, P01859; PDB code, 4HAF), hIgG3 Fc (Uniprot code, P01860; PDB code, 6D58), hIgG4 Fc[20] (Uniprot code, P01861; PDB code, 5LG1), mIgG1[21] (Uniprot code, Q99LC4; Alphafold), mIgG2a[22] (Uniprot code P01863; PDB code 5VAA) and mIgG2b[23] (Uniprot code P01867; PDB code 2RGS). **b** Structural comparison of human IgG1 (orange), IgG2 (yellow), IgG3 (blue) and IgG4 (pink). **c** Structural comparison of human IgG1 (orange) and murine IgG1 (pink), IgG2a (blue) and IgG2b (yellow).

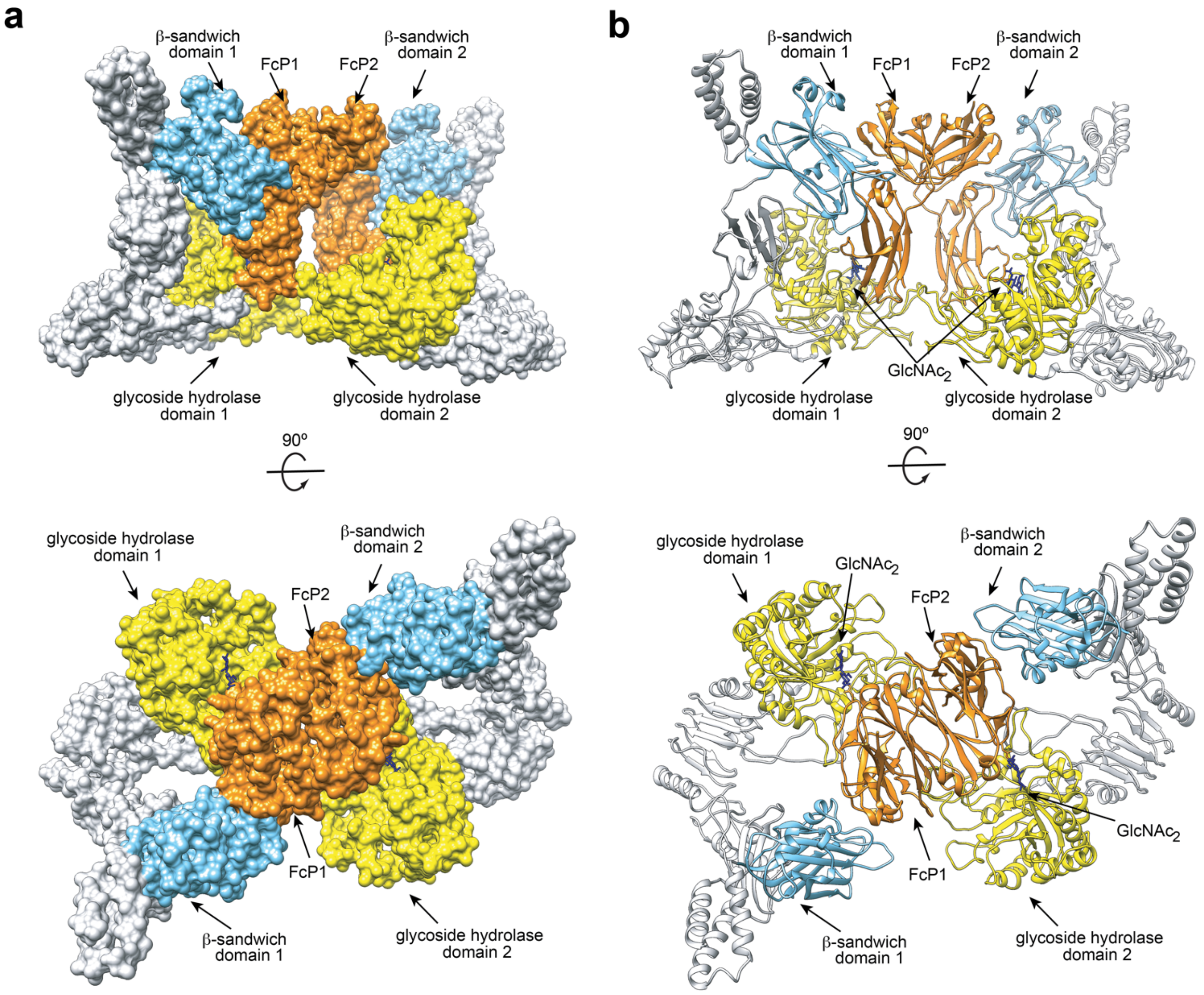


**Supplementary Fig. 38 | Model of the EndoS-Fc (2:1) complex. a,b** Two views of the surface (**a**) and cartoon representation (**b**) of the EndoS-Fc (2:1) based on our experimentally determined EndoS-Fc (1.1) cryoEM structure.

**Supplementary Videos.**

**Supplementary Video 1. Conformational motions of the EndoS$_{E235A}$-Fc complex. Component 1**

**Supplementary Video 2. Conformational motions of the EndoS$_{E235A}$-Fc complex. Component 2**

**Supplementary Video 3. Conformational motions of the EndoS$_{E235A}$-Fc complex. Component 3.**

## Supplementary References


1. Schütz, S. & Sprangers, R. Methyl TROSY spectroscopy: A versatile NMR approach to study challenging biological systems. *Prog. Nucl. Magn. Reson. Spectrosc.* **116**, 56–84 (2020).
2. Klontz, E. H. *et al.* Molecular basis of broad spectrum N-glycan specificity and processing of therapeutic IgG monoclonal antibodies by endoglycosidase S2. *ACS Cent. Sci.* **5**, 524–538 (2019).
3. Piiadov, V., Ares de Araújo, E., Oliveira Neto, M., Craievich, A. F. & Polikarpov, I. SAXSMoW 2.0: Online calculator of the molecular weight of proteins in dilute solution from experimental SAXS data measured on a relative scale. *Protein Sci.* **28**, 454–463 (2019).
4. Konarev, P. V, Volkov, V. V, Sokolova, A. V, Koch, M. H. J. & Svergun, D. I. PRIMUS: A Windows PC-based system for small-angle scattering data analysis. *J. Appl. Crystallogr.* **36**, 1277–1282 (2003).
5. Williamson, M. P. Using chemical shift perturbation to characterise ligand binding. *Prog. Nucl. Magn. Reson. Spectrosc.* **73**, 1–16 (2013).
6. Trastoy, B. *et al.* Structural basis of mammalian high-mannose N-glycan processing by human gut Bacteroides. *Nat. Commun.* **11**, 899 (2020).
7. Trastoy, B. *et al.* GH18 endo-β-N-acetylglucosaminidases use distinct mechanisms to process hybrid-type N-linked glycans. *J. Biol. Chem.* **297**, 101011 (2021).
8. Mallagaray, A. *et al.* A post-translational modification of human Norovirus capsid protein attenuates glycan binding. *Nat. Commun.* **10**, 1320 (2019).
9. Tugarinov, V. & Kay, L. E. An isotope labeling strategy for methyl TROSY spectroscopy. *J. Biomol. NMR* **28**, 165–172 (2004).
10. Tugarinov, V., Kanelis, V. & Kay, L. E. Isotope labeling strategies for the study of high-molecular-weight proteins by solution NMR spectroscopy. *Nat. Protoc.* **1**, 749–754 (2006).
11. Tugarinov, V., Hwang, P. M., Ollerenshaw, J. E. & Kay, L. E. Cross-correlated relaxation enhanced 1H[bond]13C NMR spectroscopy of methyl groups in very high molecular weight proteins and protein complexes. *J. Am. Chem. Soc.* **125**, 10420–10428 (2003).
12. Bain, A. D. Chemical exchange in NMR. *Prog. Nucl. Magn. Reson. Spectrosc.* **43**, 63–103 (2003).
13. Waudby, C. A., Ramos, A., Cabrita, L. D. & Christodoulou, J. Two-dimensional NMR lineshape analysis. *Sci. Rep.* **6**, 24826 (2016).
14. Lee, D., Hilty, C., Wider, G. & Wüthrich, K. Effective rotational correlation times of proteins from NMR relaxation interference. *J. Magn. Reson.* **178**, 72–76 (2006).
15. Naganathan, A. N. Modulation of allosteric coupling by mutations: from protein dynamics and packing to altered native ensembles and function. *Curr. Opin. Struct. Biol.* **54**, 1–9 (2019).
16. Rajasekaran, N., Suresh, S., Gopi, S., Raman, K. & Naganathan, A. N. A General Mechanism for the Propagation of Mutational Effects in Proteins. *Biochemistry* **56**, 294–305 (2017).
17. Parella, T. Towards perfect NMR: Spin-echo versus perfect-echo building blocks. *Magn. Reson. Chem.* **57**, 13–29 (2019).
18. Krapp, S., Mimura, Y., Jefferis, R., Huber, R. & Sondermann, P. Structural analysis of human IgG-Fc glycoforms reveals a correlation between glycosylation and structural integrity. *J. Mol. Biol.* **325**, 979–989 (2003).
19. Teplyakov, A., Zhao, Y., Malia, T. J., Obmolova, G. & Gilliland, G. L. IgG2 Fc structure and the dynamic features of the IgG CH2-CH3 interface. *Mol. Immunol.* **56**, 131–139 (2013).
20. Davies, A. M., Rispens, T., Ooijevaar-de Heer, P., Aalberse, R. C. & Sutton, B. J. Room temperature structure of human IgG4-Fc from crystals analysed in situ. *Mol. Immunol.* **81**, 85–91 (2017).
21. Jumper, J. *et al.* Highly accurate protein structure prediction with AlphaFold. *Nature* **596**, 583–589 (2021).

22. Labrijn, A. F. *et al.* Efficient generation of bispecific murine antibodies for pre-clinical investigations in syngeneic rodent models. *Sci. Rep.* **7**, 2476 (2017).
23. Kolenko, P. *et al.* New insights into intra- and intermolecular interactions of immunoglobulins: crystal structure of mouse IgG2b-Fc at 2.1-A resolution. *Immunology* **126**, 378–385 (2009).
