# Supplementary material for: Mechanism of antibody-specific deglycosylation and immune evasion by Streptococcal IgG-specific endoglycosidases
Source: Nat Commun. 2023 Mar 27;14:1705. doi: 10.1038/s41467-023-37215-3 (PMC10042849; doi:10.1038/s41467-023-37215-3)
Supplement: Supplementary file 2 — Description of Additional Supplementary Files [file 41467_2023_37215_MOESM2_ESM.docx]

**Description of Additional Supplementary Files**

**File Name: Supplementary Movie 1
Description:** Conformational motions of the EndoSE235A-Fc complex. Component 1.

**File Name: Supplementary Movie 2
Description:** Conformational motions of the EndoSE235A-Fc complex. Component 2.

**File Name: Supplementary Movie 3**

**Description:** Conformational motions of the EndoSE235A-Fc complex. Component 3.
