# Supplementary material for: Mechanism of antibody-specific deglycosylation and immune evasion by Streptococcal IgG-specific endoglycosidases
Source: Nat Commun. 2023 Mar 27;14:1705. doi: 10.1038/s41467-023-37215-3 (PMC10042849; doi:10.1038/s41467-023-37215-3)
Supplement: Supplementary file 6 — Reporting Summary [file 41467_2023_37215_MOESM6_ESM.pdf]

nature portfolio

Corresponding author(s): Beatriz Trastoy
Alvaro Mallagaray
Eric J. Sundberg
Marcelo E. Guerin

Last updated by author(s): 02/27/2023

Reporting Summary

Nature Portfolio wishes to improve the reproducibility of the work that we publish. This form provides structure for consistency and transparency in reporting. For further information on Nature Portfolio policies, see our Editorial Policies and the Editorial Policy Checklist.

Statistics

For all statistical analyses, confirm that the following items are present in the figure legend, table legend, main text, or Methods section.

n/a	Confirmed	
[ ]	[x]	The exact sample size (n) for each experimental group/condition, given as a discrete number and unit of measurement
[ ]	[x]	A statement on whether measurements were taken from distinct samples or whether the same sample was measured repeatedly
[ ]	[x]	The statistical test(s) used AND whether they are one- or two-sided<br>*Only common tests should be described solely by name; describe more complex techniques in the Methods section.*
[x]	[ ]	A description of all covariates tested
[x]	[ ]	A description of any assumptions or corrections, such as tests of normality and adjustment for multiple comparisons
[ ]	[x]	A full description of the statistical parameters including central tendency (e.g. means) or other basic estimates (e.g. regression coefficient) AND variation (e.g. standard deviation) or associated estimates of uncertainty (e.g. confidence intervals)
[ ]	[x]	For null hypothesis testing, the test statistic (e.g. F, t, r) with confidence intervals, effect sizes, degrees of freedom and P value noted<br>*Give P values as exact values whenever suitable.*
[x]	[ ]	For Bayesian analysis, information on the choice of priors and Markov chain Monte Carlo settings
[x]	[ ]	For hierarchical and complex designs, identification of the appropriate level for tests and full reporting of outcomes
[x]	[ ]	Estimates of effect sizes (e.g. Cohen's d, Pearson's r), indicating how they were calculated

Our web collection on statistics for biologists contains articles on many of the points above.

Software and code

Policy information about availability of computer code

Data collection	Agilent 1290 Infinity II LC System equipped with a 50 mm PLRP-S column from Agilent with 1000 Å pore size. The LC system is attached to an Agilent 6560 Ion Mobility (IM) quadrupole- time of flight (Q-TOF) mass spectrometer (Agilent, Santa Clara, CA)
Data analysis	cryosparc v3.2.0<br>relion 3.1.2<br>phenix-1.18.2-3874<br>coot 0.8.9.2<br>UCSF Chimera 1.14<br>USCF ChimeraX 1.3<br>pymol V_2.5<br>TITAN v1.6-12-g9041<br>Matlab R2019b<br>CCPNMR Analysis v2.4.2.<br>TopSpin 4.0.6

For manuscripts utilizing custom algorithms or software that are central to the research but not yet described in published literature, software must be made available to editors and reviewers. We strongly encourage code deposition in a community repository (e.g. GitHub). See the Nature Portfolio guidelines for submitting code & software for further information.

Data

Policy information about availability of data

All manuscripts must include a data availability statement. This statement should provide the following information, where applicable:

- Accession codes, unique identifiers, or web links for publicly available datasets
- A description of any restrictions on data availability
- For clinical datasets or third party data, please ensure that the statement adheres to our policy

The atomic coordinates of EndoSE235A-Fc have been deposited in the Protein Data Bank (PDB) under the accession code 8A64 [http://doi.org/10.2210/pdb8A64/pdb], and the cryoEM map are deposited in the Electron Microscopy Data Bank (EMDB) under the accession code EMD-15205 [https://www.ebi.ac.uk/pdbe/entry/emdb/EMD-15205. Previously published PDB structures used in this study are available under the accession codes: 6MDS [http://doi.org/10.2210/pdb6MDS/pdb], 4NUY [http://doi.org/10.2210/pdb4NUY/pdb], 2DTS [http://doi.org/10.2210/pdb2DTS/pdb], 1H3X [http://doi.org/10.2210/pdb1H3X/pdb] and 6EN3 [http://doi.org/10.2210/pdb6EN3/pdb]. All other data are available from the corresponding authors upon request. Source data are provided with this paper.

Human research participants

Policy information about studies involving human research participants and Sex and Gender in Research.

Reporting on sex and gender	This is not relevant for this study
Population characteristics	This is not relevant for this study
Recruitment	This is not relevant for this study
Ethics oversight	This is not relevant for this study

Note that full information on the approval of the study protocol must also be provided in the manuscript.

Field-specific reporting

Please select the one below that is the best fit for your research. If you are not sure, read the appropriate sections before making your selection.

☒ Life sciences ☐ Behavioural & social sciences ☐ Ecological, evolutionary & environmental sciences

For a reference copy of the document with all sections, see nature.com/documents/nr-reporting-summary-flat.pdf

Life sciences study design

All studies must disclose on these points even when the disclosure is negative.

Sample size	We performed the experiments in duplicates or triplicates. We have included this information in the relevant figure legends.
Data exclusions	No data excluded from the activity and binding experiments
Replication	We performed the experiments in duplicates or triplicates. All the activity and binding experiments were reproducible
Randomization	This is not relevant for this study because we did not need to prevent bias in our activity and binding experiments
Blinding	This is not relevant for this study because we did not need to prevent bias in our activity and binding experiments

Reporting for specific materials, systems and methods

We require information from authors about some types of materials, experimental systems and methods used in many studies. Here, indicate whether each material, system or method listed is relevant to your study. If you are not sure if a list item applies to your research, read the appropriate section before selecting a response.

Materials & experimental systems

n/a	Involved in the study
☐	☒ Antibodies
☐	☒ Eukaryotic cell lines
☒	☐ Palaeontology and archaeology
☒	☐ Animals and other organisms
☒	☐ Clinical data
☒	☐ Dual use research of concern

Methods

n/a	Involved in the study
☒	☐ ChIP-seq
☒	☐ Flow cytometry
☒	☐ MRI-based neuroimaging

Antibodies

Antibodies used	Rituximab (RITUXAN, Genentech) was kindly provided courtesy of the University of Maryland Greenebaum Comprehensive Cancer Center. No catalogue number.
Validation	We did not need to validate the antibody because our experiments are focus on the Fc region.

Eukaryotic cell lines

Policy information about cell lines and Sex and Gender in Research

Cell line source(s)	HEK293T (ATTC, cat. No.: CRL-3216) and Expi293 (ThermoFisher Scientific, cat.No.: A14527) cells
Authentication	None of the cell used were authenticated
Mycoplasma contamination	No
Commonly misidentified lines (See ICLAC register)	N/A
